# Tools and techniques for computational reproducibility

**DOI:** 10.1186/s13742-016-0135-4

**Published:** 2016-07-11

**Authors:** Stephen R. Piccolo, Michael B. Frampton

**Affiliations:** 1 Department of Biology, Brigham Young University, Provo, UT 84602 USA; 2 Department of Computer Science, Brigham Young University, Provo, UT USA

**Keywords:** Computational reproducibility, Practice of science, Literate programming, Virtualization, Software containers, Software frameworks

## Abstract

When reporting research findings, scientists document the steps they followed so that others can verify and build upon the research. When those steps have been described in sufficient detail that others can retrace the steps and obtain similar results, the research is said to be reproducible. Computers play a vital role in many research disciplines and present both opportunities and challenges for reproducibility. Computers can be programmed to execute analysis tasks, and those programs can be repeated and shared with others. The deterministic nature of most computer programs means that the same analysis tasks, applied to the same data, will often produce the same outputs. However, in practice, computational findings often cannot be reproduced because of complexities in how software is packaged, installed, and executed—and because of limitations associated with how scientists document analysis steps. Many tools and techniques are available to help overcome these challenges; here we describe seven such strategies. With a broad scientific audience in mind, we describe the strengths and limitations of each approach, as well as the circumstances under which each might be applied. No single strategy is sufficient for every scenario; thus we emphasize that it is often useful to combine approaches.

**Electronic supplementary material:**

The online version of this article (doi:10.1186/s13742-016-0135-4) contains supplementary material, which is available to authorized users.

## Background

When reporting research, scientists document the steps they followed to obtain their results. If the description is comprehensive enough that they and others can repeat the procedures and obtain semantically consistent results, the findings are considered to be “reproducible” [1–6]. Reproducible research forms the basic building blocks of science, insofar as it allows researchers to verify and build on each other’s work with confidence.

Computers play an increasingly important role in many scientific disciplines [7–10]. For example, in the United Kingdom, 92 % of academic scientists use some type of software in their research, and 69 % of scientists say their research is feasible only with software tools [11]. Thus efforts to increase scientific reproducibility should consider the ubiquity of computers in research.

Computers present both opportunities and challenges for scientific reproducibility. On one hand, the deterministic nature of most computer programs means that identical results can be obtained from many computational analyses applied to the same input data [12]. Accordingly, computational research can be held to a high reproducibility standard. On the other hand, even when no technical barrier prevents reproducibility, scientists often cannot reproduce computational findings because of complexities in how software is packaged, installed, and executed—and because of limitations associated with how scientists document these steps [13]. This problem is acute in many disciplines, including genomics, signal processing, and ecological modeling [14–16], which have large data sets and rapidly evolving computational tools. However, the same problem can affect any scientific discipline requiring computers for research. Seemingly minor differences in computational approaches can have major influences on analytical outputs [12, 17–22], and the effects of these differences may exceed those resulting from experimental factors [23].

Journal editors, funding agencies, governmental institutions, and individual scientists have increasingly made calls for the scientific community to embrace practices to support computational reproducibility [24–31]. This movement has been motivated, in part, by scientists’ failed efforts to reproduce previously published analyses. For example, Ioannidis et al. evaluated 18 published research studies that used computational methods to evaluate gene expression data, but they were able to reproduce only two of those studies [32]. In many cases, the culprit was a failure to share the study’s data; however, incomplete descriptions of software-based analyses were also common. Nekrutenko and Taylor examined 50 papers that analyzed next-generation sequencing data and observed that fewer than half provided any details about software versions or parameters [33]. Recreating analyses that lack such details can require hundreds of hours of effort [34] and may be impossible, even after consulting the original authors. Failure to reproduce research may also lead to careerist effects, including retractions [35].

Noting such concerns, some journals have emphasized the value of placing computer code in open access repositories. It is most useful when scientists provide direct access to an archived version of the code via a uniform resource locator (URL). For example, Zenodo.org and figshare.com provide permanent digital object identifiers (DOI) that can link to software code (and other digital objects) used in publications. In addition, some journals have extended requirements for “Methods” sections, now asking researchers to provide detailed descriptions of 1) how to install software and its dependencies, and 2) what parameters and data preprocessing steps are used in analyses [10, 24]. A 2012 Institute of Medicine report emphasized that, in addition to computer code and research data, “fully specified computational procedures” should be made available to the scientific community [25]. The report’s authors elaborated that such procedures should include “all of the steps of computational analysis”, and that “all aspects of the analysis need to be transparently reported” [25]. Such policies represent important progress. However, it is ultimately the responsibility of individual scientists to ensure that others can verify and build upon their analyses.

Describing a computational analysis sufficiently—such that others can re-execute, validate, and refine it—requires more than simply stating what software was used, what commands were executed, and where to find the source code [13, 27, 36–38]. Software is executed within the context of an operating system (for example, Windows, Mac OS, or Linux), which enables the software to interface with computer hardware. In addition, most software relies on a hierarchy of software dependencies, which perform complementary functions and must be installed alongside the main software tool. One version of a given software tool or dependency may behave differently or have a different interface than another version of the same software. In addition, most analytical software offers a range of parameters (or settings) that the user can specify. If any of these variables differs from those used by the original experimenter, the software may not execute properly or analytical outputs may differ considerably from those observed by the original experimenter.

Scientists can use various tools and techniques to overcome these challenges and to increase the likelihood that their computational analyses will be reproducible. These techniques range in complexity from simple (e.g., providing written documentation) to advanced (e.g., providing a virtual environment that includes an operating system and all the software necessary to execute the analysis). This review describes seven strategies across this spectrum. We describe many of the strengths and limitations of each approach, as well as the circumstances under which each might be applied. No single strategy will be sufficient for every scenario; therefore, in many cases, it will be most practical to combine multiple approaches. This review focuses primarily on the computational aspects of reproducibility. The related topics of empirical reproducibility, statistical reproducibility, data sharing, and education about reproducibility have been described elsewhere [39–46]. We believe that with greater awareness and understanding of computational reproducibility techniques, scientists—including those with limited computational experience—will be more apt to perform computational research in a reproducible manner.

## Narrative descriptions are a simple but valuable way to support computational reproducibility

The most fundamental strategy for enabling others to reproduce a computational analysis is to provide a detailed, written description of the process. For example, when reporting computational results in a research article, authors customarily provide a narrative that describes the software they used and the analytical steps they followed. Such narratives can be invaluable in enabling others to evaluate the scientific approach and to reproduce the findings. In many situations—for example, when software execution requires user interaction or when proprietary software is used—narratives are the only feasible option for documenting such steps. However, even when a computational analysis uses open-source software and can be fully automated, narratives help others understand how to re-execute an analysis.

Although most articles about research that uses computational methods provide some type of narrative, these descriptions often lack sufficient detail to enable others to retrace those steps [32, 33]. Narrative descriptions should indicate the operating system(s), software dependencies, and analytical software that were used, and how to obtain them. In addition, narratives should indicate the exact software versions used, the order in which they were executed, and all non-default parameters that were specified. Such descriptions should account for the fact that computer configurations can differ vastly, even for computers with the same operating system. Because it can be difficult for scientists to remember such details after the fact, it is best to record this information throughout the research process, rather than at the time of manuscript preparation [8].

The following sections describe techniques for automating computational analyses. These techniques can diminish the need for scientists to write narratives. However, because it is often impractical to automate all computational steps, we expect that, for the foreseeable future, narratives will play a vital role in enabling computational reproducibility.

## Custom scripts and code can automate research analysis

**Fig. 1:**
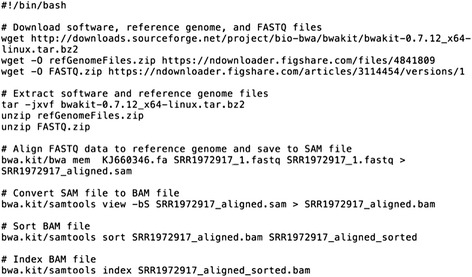
Example of a command line script. This script can be used to align DNA sequence data to a reference genome. First, it downloads the software and data files necessary for the analysis. Then, it extracts (“unzips”) these files, and aligns the data to a reference genome for Ebola virus. Finally, it converts, sorts, and indexes the aligned data. See Additional file 1 for an executable version of this script

Scientific software can often be executed in an automated manner via text-based commands. Using such commands—via a command-line interface—scientists can indicate the software program(s) to be executed and which parameter(s) should be used. When multiple commands must be executed, they can be compiled into scripts specifying the order in which the commands should be executed (Fig. 1; Additional file 1). In many cases, scripts also include commands for installing and configuring software. Such scripts serve as valuable documentation not only for individuals who wish to re-execute the analysis, but also for the researcher who performed the original analysis [47]. In these cases, no amount of narrative is an adequate substitute for providing the actual commands that were used.

**Fig. 2:**
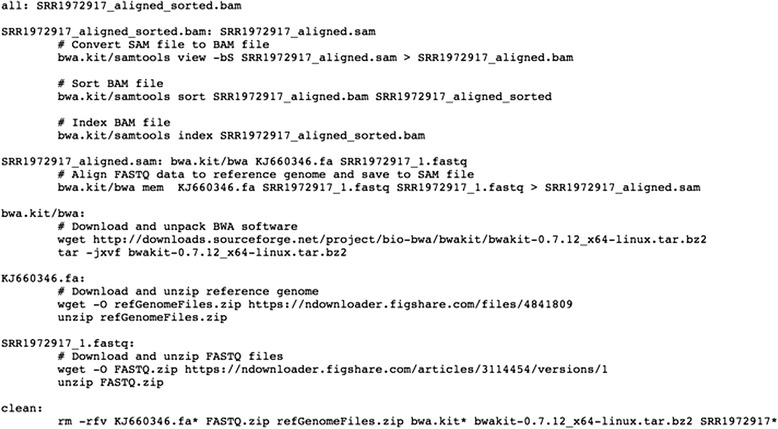
Example of a Make file. This file performs the same function as the command line script shown in Fig. 1, except that it is formatted for the Make utility. Accordingly, it is structured so that specific tasks must be executed before other tasks, in a hierarchical manner. See Additional file 2 for an executable version of this file

When writing command-line scripts, it is essential to explicitly document any software dependencies and input data that are required for each step in the analysis. The Make utility [48, 49] provides one way to specify such requirements [36]. Before any command is executed, Make verifies that each documented dependency is available. Accordingly, researchers can use Make files (scripts) to specify a full hierarchy of operating system components and dependent software that must be present to perform the analysis (Fig. 2; Additional file 2). In addition, Make can automatically identify any commands that can be executed in parallel, potentially reducing the amount of time required for the analysis. Although Make was originally designed for UNIX-based operating systems (such as Mac OS or Linux), similar utilities have since been developed for Windows operating systems [50]. Table 1 lists various utilities that can be used to automate software execution.

**Table 1:** Utilities that can be used to automate software execution

• GNU Make and Make for Windows: tools for building software from source files and for ensuring that the software’s dependencies are met.
• Snakemake [109]: an extension of Make that provides a more flexible syntax and makes it easier to execute tasks in parallel.
• BPipe [110]: a tool that provides a flexible syntax for users to specify commands to be executed; it maintains an audit trail of all commands that have been executed.
• GNU Parallel [111]: a tool for executing commands in parallel across one or more computers.
• Makeflow [112]: a tool that can execute commands simultaneously on various types of computer architectures, including computer clusters and cloud environments.
• SCONS [113]: an alternative to GNU Make that enables users to customize the process of building and executing software using scripts written in the Python programming language.
• CMAKE.org: a tool that enables users to execute Make scripts more easily on multiple operating systems.

As well as creating scripts to execute existing software, many researchers also create new software by writing computer code in a programming language such as Python, C++, Java, or R. Such code may perform relatively simple tasks, such as reformatting data files or invoking third-party software. In other cases, computer code may constitute a manuscript’s key intellectual contribution.

Whether analysis steps are encoded in scripts or as computer code, scientists can support reproducibility by publishing these artifacts alongside research papers. By doing so, authors enable readers to evaluate the analytical approach in full detail and to extend the analysis more readily [51]. Although scripts and code may be included alongside a manuscript as supplementary material, a better alternative is to store them in a public repository with a permanent URL. It is often also useful to store code in a version control system (VCS) [8, 9, 47], and to share it via Web-based services like GitHub.com or Bitbucket.org [52]. With such a VCS repository, scientists can track the different versions of scripts and code that have been developed throughout the evolution of the research project. In addition, outside observers can see the full version history, contribute revisions to the code, and reuse the code for their own purposes [53]. When submitting a manuscript, the authors may “tag” a specific version of the repository that was used for the final analysis described in the manuscript.

## Software frameworks enable easier handling of software dependencies

Virtually all computer scripts and code relies on external software dependencies and operating system components. For example, suppose a research study required a scientist to apply Student’s *t*-test. Rather than write code to implement this statistical test, the scientist would likely find an existing software library that implements the test and then invoke that library from their code. Much time can be saved with this approach, and a wide range of software libraries are freely available. However, software libraries change frequently; invoking the wrong version of a library may result in an error or an unexpected output. Thus, to enable others to reproduce an analysis, it is critical to indicate which dependencies (and versions thereof) must be installed.

One way to address this challenge is to build on a pre-existing software framework, which makes it easier to access software libraries that are commonly used to perform specific types of analysis task. Typically, such frameworks also make it easier to download and install software dependencies, and to ensure that the versions of software libraries and their dependencies are compatible with each other. For example, Bioconductor [54], created for the R statistical programming language [55], is a popular framework that contains hundreds of software packages for analyzing biological data. The Bioconductor framework facilitates versioning, documenting, and distributing code. Once a software library has been incorporated into Bioconductor, other researchers can find, download, install, and configure it on most operating systems with relative ease. In addition, Bioconductor installs software dependencies automatically. These features ease the process of performing an analysis, and can help with reproducibility. Various software frameworks exist for other scientific disciplines [56–61]. General purpose tools for managing software dependencies also exist, for example, Apache Ivy [62] and Puppet [50].

To best support reproducibility, software frameworks should make it easy for scientists to download and install previous versions of a software tool, as well as previous versions of dependencies. Such a design enables other scientists to reproduce analyses that were conducted with previous versions of a software framework. In the case of Bioconductor, considerable extra work may be required to install specific versions of Bioconductor software and their dependencies. To overcome these limitations, scientists may use a software container or virtual machine to package together the specific versions they used in an analysis. Alternatively, they might use third-party solutions such as the aRchive project [63].

## Literate programming combines narratives with code

Although narratives, scripts, and computer code individually support reproducibility, there is additional value in combining these entities. Even though a researcher may provide computer code alongside a research paper, other scientists may have difficulty interpreting how the code accomplishes specific tasks. A longstanding way to address this problem is via code comments: human-readable annotations interspersed throughout computer code. However, code comments and other types of documentation often become outdated as code evolves throughout the analysis process [64]. One way to overcome this problem is to use a technique called literate programming [65]. In this approach, the scientist writes a narrative of the scientific analysis and intermingles code directly within the narrative. As the code is executed, a document is generated that includes the code, narratives, and any outputs (e.g., figures, tables) of the code. Accordingly, literate programming helps ensure that readers understand exactly how a particular research result was obtained. In addition, this approach motivates the scientist to keep the target audience in mind when performing a computational analysis, rather than simply to write code that a computer can parse [65]. Consequently, by reducing barriers of understanding among scientists, literate programming can help to engender greater trust in computational findings.

One popular literate programming tool is Jupyter [66]. Using Jupyter.org’s Web-based interface, scientists can create interactive “notebooks” that combine code, data, mathematical equations, plots, and rich media [67]. Originally known as IPython, and previously designed exclusively for the Python programming language, Jupyter now makes it possible to execute code in many different programming languages. Such functionality may be important to scientists who prefer to combine the strengths of different programming languages.

knitr [68] has also gained considerable popularity as a literate programming tool. It is written in the R programming language, and thus can be integrated seamlessly with the array of statistical and plotting tools available in that environment. However, like Jupyter, knitr can execute code written in multiple programming languages. Commonly, knitr is applied to documents that have been authored using RStudio [69], an open-source tool with advanced editing and package management features.

**Fig. 3:**
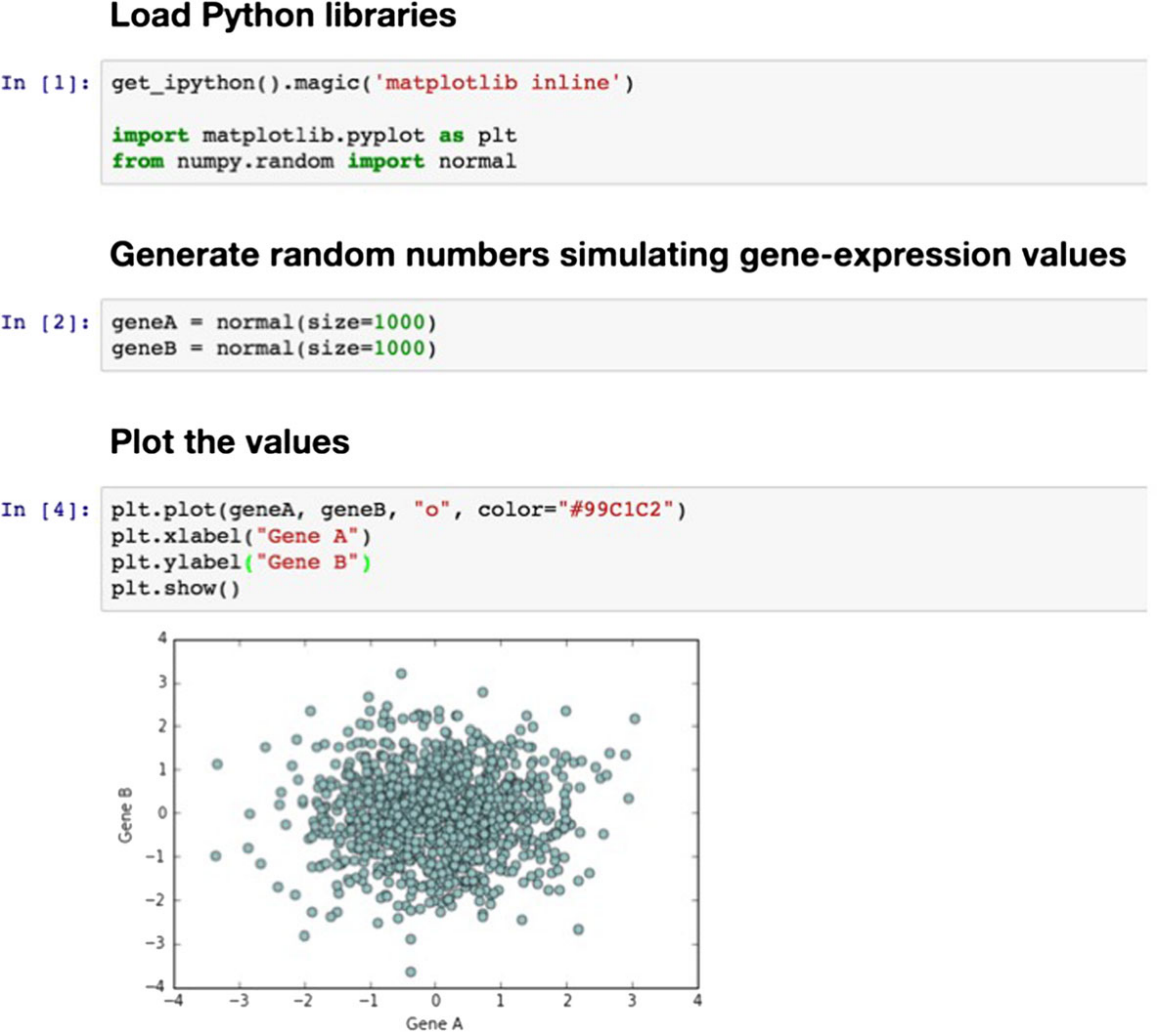
Example of a Jupyter notebook. This example contains code (in the Python programming language) for generating random numbers and plotting them in a graph within a Jupyter notebook. Importantly, the code and output object (graph) are contained within the same document. See Additional file 3 for an executable version of the notebook

**Fig. 4:**
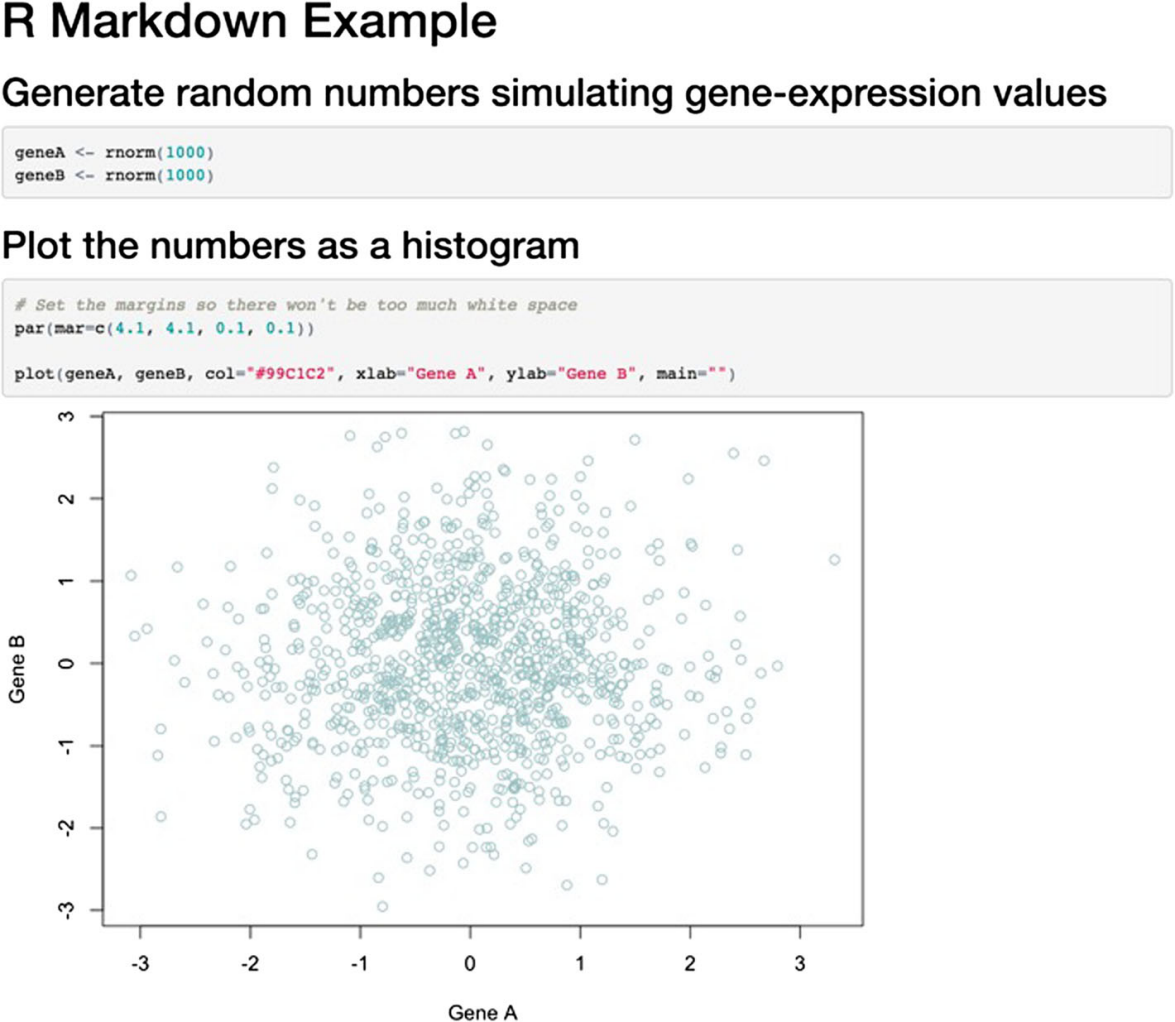
Example of a document created using knitr. This example contains code (in the R language) for generating random numbers and plotting them on a graph. The knitr tool was used to generate the document, which combines the code and the output object (figure). See Additional file 4 for an executable version of this document

Jupyter notebooks and knitr reports can be saved in various output formats, including hypertext markup language (HTML) and portable document format (PDF; see examples in Figs. 3 and 4; Additional files 3 and 4). Increasingly, scientists include such documents as supplementary materials to journal manuscripts, enabling others to repeat analysis steps and recreate manuscript figures [70–73].

Scientists typically use literate programming tools for data analysis tasks that can be executed interactively, in a modest amount of time (e.g., minutes or hours). However, it is possible to execute Jupyter or knitr at the command line; thus longer running tasks can be executed on high-performance computers.

Literate programming notebooks are suitable for research analyses that require a modest amount of computer code. For analyses needing larger amounts of code, more advanced programming environments may be more suitable, perhaps in combination with a “literate documentation” tool such as Dexy.it.

## Workflow management systems enable software to be executed via a graphical user interface

Writing computer scripts and code seems daunting to many researchers. Although various courses and tutorials are helping to make this task less formidable [46, 74–76], many scientists use “workflow management systems” to facilitate the execution of scientific software [77]. Typically managed via a graphical user interface, workflow management systems enable scientists to upload data and process them using existing tools. For multistep analyses, the output from one tool can be used as input to additional tools, resulting in a series of commands known as a workflow.

Galaxy [78, 79] has gained considerable popularity within the bioinformatics community, especially for performing next-generation sequencing analysis. As users construct workflows, Galaxy provides descriptions of how software parameters should be used, examples of how input files should be formatted, and links to relevant discussion forums. To help with processing large data sets and computationally complex algorithms, Galaxy also provides an option to execute workflows on cloud-computing services [80]. In addition, researchers can share workflows with each other at UseGalaxy.org; this feature has enabled the Galaxy team to build a community that encourages reproducibility, helps define best practices, and reduce the time required for novices to get started.

Various other workflow systems are freely available to the research community (see Table 2). For example, VisTrails.org is used by researchers from many disciplines, including climate science, microbial ecology, and quantum mechanics [81]. It enables scientists to design visual workflows, and connect data inputs with analytical modules and the resulting outputs. In addition, VisTrails tracks a full history of how each workflow was created. This capability, referred to as “retrospective provenance”, makes it possible for others to not only reproduce the final version of an analysis, but also to examine previous incarnations of the workflow and how each change influenced the analytical outputs [82].

**Table 2:** Workflow management tools freely available to the research community

• Galaxy [78, 79]
• VisTrails [81]
• Kepler-project.org [114]
• CyVerse.org (formerly known as The iPlant Collaborative) [115]
• GenePattern [116–118]
• Taverna.org.uk [119]
• LONI Pipeline [120, 121]

Although workflow management systems offer many advantages, users must accept tradeoffs. For example, although the teams that develop these tools often provide public servers where users can execute workflows, many scientists share these resources, limiting the computational power or storage space available to execute large-scale analyses in a timely manner. As an alternative, many scientists install these systems on their own computers; however, configuring and supporting them requires time and expertise. In addition, if a workflow tool does not yet provide a module to support a given analysis, the scientist must create one. This task constitutes additional overheads; however, utilities such as the Galaxy Tool Shed [83] are helping to facilitate this process.

## Virtual machines encapsulate an entire operating system and software dependencies

Whether within a literate programming notebook, or via a workflow management system, an operating system and relevant software dependencies must be installed before an analysis is executed. The process of identifying, installing, and configuring such dependencies consumes a considerable amount of scientists’ time. Different operating systems (and versions thereof) may require different installation and configuration steps. Furthermore, earlier versions of software dependencies, which may currently be installed on a given computer, may be incompatible with—or produce different outputs than—newer versions.

**Fig. 5:**
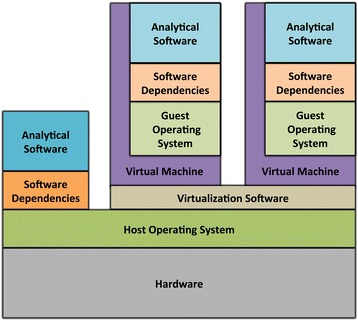
Architecture of virtual machines. Virtual machines encapsulate analytical software and dependencies within a “guest” operating system, which may be different to the main (“host”) operating system. A virtual machine executes in the context of virtualization software, which runs alongside other software installed on the computer

One solution is to use virtual machines, which can encapsulate an entire operating system and all software, scripts, code, and data necessary to execute a computational analysis [84, 85] (Fig. 5). Using virtualization software such as VirtualBox or VMWare (see Table 3), a virtual machine can be executed on practically any desktop, laptop, or server, irrespective of the main (“host”) operating system on the computer. For example, even though a scientist’s computer may be running a Windows operating system, they may perform an analysis on a Linux operating system that is running concurrently—within a virtual machine—on the same computer. The scientist has full control over the virtual (“guest”) operating system, and thus can install software and modify configuration settings as necessary. In addition, a virtual machine can be constrained to use specific amounts of computational resources (e.g., computer memory, processing power), thus enabling system administrators to ensure that multiple virtual machines can be executed simultaneously on the same computer without impacting each other’s performance. After executing an analysis, the scientist can export the entire virtual machine to a single, binary file. Other scientists can then use this file to reconstitute the same computational environment that was used for the original analysis. With a few exceptions (see Discussion), these scientists will obtain exactly the same results as the original scientist. This process provides the added benefits that 1) the scientist must only document the installation and configuration steps for a single operating system, 2) other scientists need only install the virtualization software and not individual software components, and 3) analyses can be re-executed indefinitely, so long as the virtualization software remains compatible with current computer systems [86]. The fact that a team of scientists can employ virtual machines to ensure that each team member has the same computational environment is also useful because team members may have different configurations on their host operating systems.

**Table 3:** Virtual machine software

Virtualization hypervisors:
• VirtualBox.org (open source)
• XenProject.org (open source)
• VMWare.com (partially open source)
Virtual machine management tools:
• VagrantUP.com (open source)
• Vortex (open source) [122]

One criticism of using virtual machines to support computational reproducibility is that virtual machine files are large (typically multiple gigabytes), especially if they include raw data files. This imposes a barrier for researchers to share virtual machines with the research community. One option is to use cloud-computing services (see Table 4). Scientists can execute an analysis in the cloud, take a “snapshot” of their virtual machine, and share it with others in that environment [84, 87]. Cloud-based services typically provide repositories where virtual machine files can easily be stored and shared among users. Despite these advantages, some researchers may prefer their data to reside on local computers, rather than in the cloud—at least while the research is being performed. In addition, cloud-based services may use proprietary software, so virtual machines may only be executable within each provider’s infrastructure. Furthermore, to use a cloud service provider, scientists may need to activate a fee-based account.

**Table 4:** Commercial cloud-service providers

• Amazon Web Services [123]
• Rackspace.com/Cloud
• Google Cloud Platform [124]
• Windows Azure [125]

When using virtual machines to support reproducibility, it is important that other scientists can not only re-execute the analysis, but also examine the scripts and code used within the virtual machine [88]. Although it is possible for others to examine the contents of a virtual machine directly, it is preferable to store the scripts and code in public repositories—separately from the virtual machine—so others can examine and extend the analysis more easily [89]. In addition, scientists can use a virtual machine that has been prepackaged for a particular research discipline. For example, CloudBioLinux contains a variety of bioinformatics tools commonly used by genomics researchers [90]. The scripts for building this virtual machine are stored in a public repository [91].

Scientists can automate the process of building and configuring virtual machines using tools such as Vagrant or Vortex (see Table 3). For either tool, users can write text-based configuration files that provide instructions for building virtual machines and allocating computational resources to them. In addition, these configuration files can be used to specify analysis steps [89]. Because these files are text based and relatively small (usually a few kilobytes), scientists can share them easily and track different versions of the files via source control repositories. This approach also mitigates problems that might arise during the analysis stage. For example, even when a computer’s host operating system must be reinstalled because of a computer hardware failure, the virtual machine can be recreated with relative ease.

## Software containers ease the process of installing and configuring dependencies

**Fig. 6:**
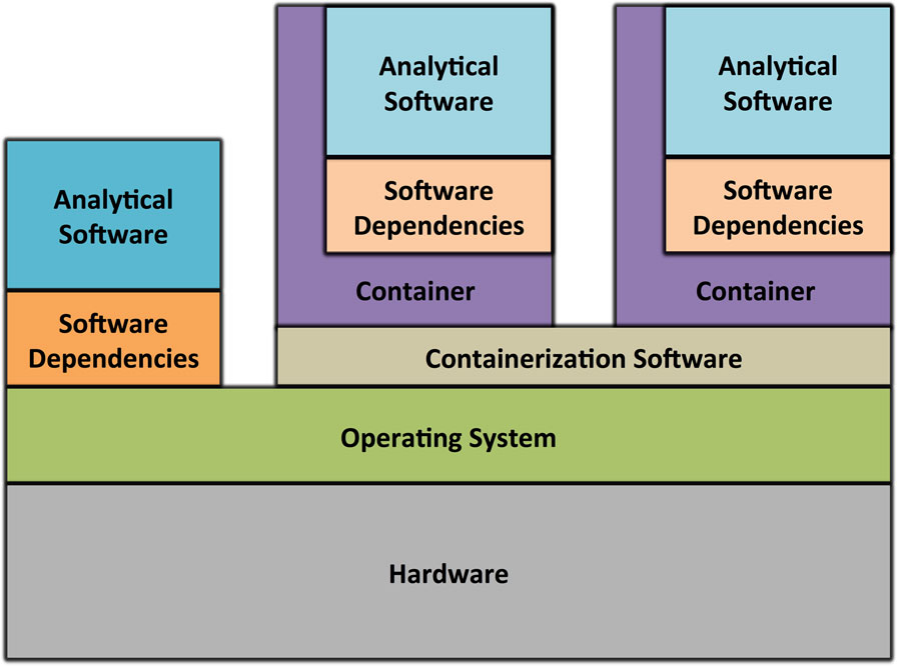
Architecture of software containers. Software containers encapsulate analytical software and dependencies. In contrast to virtual machines, containers execute within the context of the computer’s main operating system

Software containers are a lighter weight alternative to virtual machines. Like virtual machines, containers encapsulate operating system components, scripts, code, and data into a single package that can be shared with others. Thus, as with virtual machines, analyses executed within a software container should produce identical outputs, irrespective of the underlying operating system or the software that may be installed outside the container (see Discussion for caveats). As is true for virtual machines, multiple containers can be executed simultaneously on a single computer, and each container may contain different software versions and configurations. However, whereas virtual machines include an entire operating system, software containers interface directly with the computer’s main operating system and extend it as needed (Fig. 6). This design provides less flexibility than virtual machines because containers are specific to a given type of operating system; however, containers require considerably less computational overhead than virtual machines, and can be initialized much more quickly [92].

The open source Docker.com utility, which has gained popularity among informaticians since its release in 2013, provides the ability to build, execute, and share software containers for Linux-based operating systems. Users specify a Docker container’s contents using text-based commands. These instructions can be placed in a “Dockerfile”, which other scientists can use to rebuild the container. As with virtual machine configuration files, Dockerfiles are text based, so they can be shared easily, and can be tracked and versioned in source control repositories. Once a Docker container has been built, its contents can be exported to a binary file; these files are generally smaller than virtual machine files, so they can be shared more easily—for example, via hub.Docker.com.

A key feature of Docker containers is that their contents can be stacked in distinct layers (or “images”). Each image includes software components to address a particular need. Within a given research lab, scientists might create general purpose images to support functionality for multiple projects, and specialized images to address the needs of specific projects. An advantage of Docker’s modular design is that when images within a container are updated, Docker only needs to track the specific components that have changed; users who wish to update to a newer version must download a relatively small update. In contrast, even a minor change to a virtual machine would require users to export and reshare the entire virtual machine.

Scientists have begun to share Docker images that enable others to execute analyses described in research papers [93–95], and to facilitate benchmarking efforts among researchers in a given subdiscipline. For example, nucleotid.es is a catalog of genome-assembly tools that have been encapsulated in Docker images [96, 97]. Genome assembly tools differ considerably in the dependencies they require, and in the parameters they support. This project provides a means to standardize these assemblers, circumvent the need to install dependencies for each tool, and perform benchmarks across the tools. Such projects may help to reduce the reproducibility burden on individual scientists.

The use of Docker containers for reproducible research comes with caveats. Individual containers are stored and executed in isolation from other containers on the same computer; however, because all containers on a given machine share the same operating system, this isolation is not as complete as it is with virtual machines. This means, for example, that a given container is not guaranteed to have access to a specific amount of computer memory or processing power—multiple containers may have to compete for these resources [92]. In addition, containers may be more vulnerable to security breaches [92]. Because Docker containers can only be executed on Linux-based operating systems, they must be executed within a virtual machine on Windows and Mac operating systems. Docker provides installation packages to facilitate this integration; however, the overhead of using a virtual machine offsets some of the performance benefits of using containers.

Efforts are ongoing to develop and refine software container technologies. Table 5 lists various tools that are currently available. In the coming years, these technologies promise to play an influential role within the scientific community.

**Table 5:** Open-source containerization software

• Docker.com
• LinuxContainers.org
• lmctfy [126]
• OpenVZ.org
• Warden [127]

## Conclusions

Scientific advancement requires researchers to explicitly document the research steps they performed and to transparently share those steps with other researchers. This review provides a comprehensive, though not exhaustive, list of techniques that can help meet these requirements for computational analyses. Science philosopher Karl Popper contended that, “[w]e do not take even our own observations quite seriously, or accept them as scientific observations, until we have repeated and tested them” [2]. Indeed, in many cases, the individuals who benefit most from computational reproducibility are those who performed the original analysis, but reproducible and transparent practices can also increase the level at which a scientist’s work is accepted by other scientists [47, 98]. When other scientists can reproduce an analysis and determine exactly how its conclusions were drawn, they may be more apt to cite and build upon the work. In contrast, when others fail to reproduce research findings, it can derail scientific progress and may lead to embarrassment, accusations, and retractions.

**Fig. 7:**
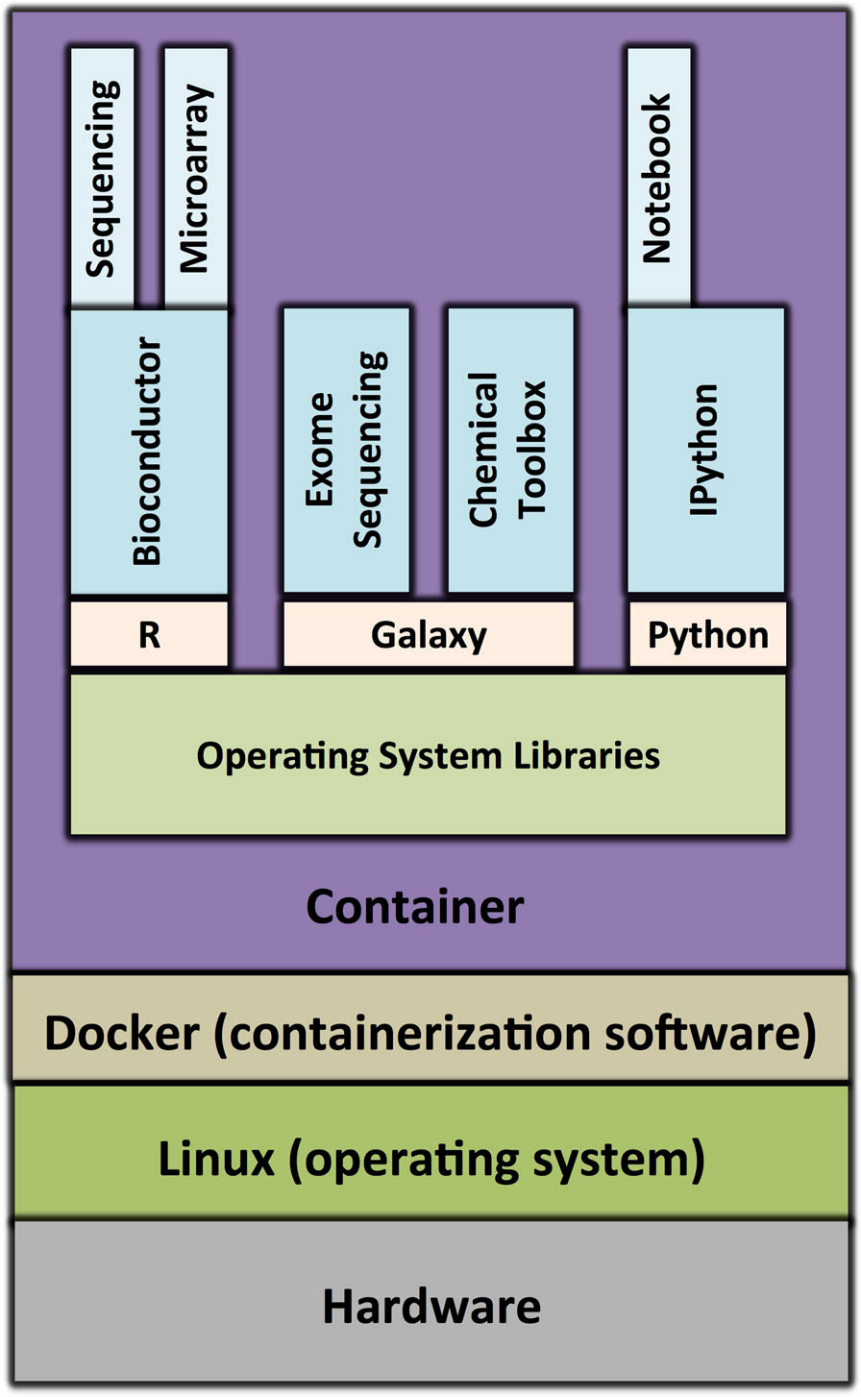
Example of a Docker container for genomics research. This container would enable researchers to preprocess various types of molecular data, using tools from Bioconductor and Galaxy, and to analyze the resulting data within a Jupyter notebook. Each box within the container represents a distinct Docker image. These images are layered such that some images depend on others (for example, the Bioconductor image depends on R). At its base, the container includes operating system libraries, which may not be present (or may be configured differently) on the computer’s main operating system

We have described seven tools and techniques for facilitating computational reproducibility. None of these approaches is sufficient for every scenario in isolation; rather, scientists will often find value in combining approaches. For example, a researcher who uses a literate programming notebook (that combines narratives with code) might incorporate the notebook into a software container so that others can execute it without needing to install specific software dependencies. The container might also include a workflow management system to ease the process of integrating multiple tools and incorporating best practices for the analysis. This container could be packaged within a virtual machine or cloud-computing environment to ensure that it can be executed consistently (see Fig. 7). Binder [99] and Everware [100] are two services that allow researchers to execute Jupyter notebooks within a Web browser, using a Docker container to package the underlying software, and a cloud-computing environment to execute it. Although still under active development, such services may be harbingers of the future for computationally reproducible science.

The call for computational reproducibility relies on the premise that reproducible science will bolster the efficiency of the overall scientific enterprise [101]. Although reproducible practices may require additional time and effort, these practices provide ancillary benefits that help offset those expenditures [47]. Primarily, scientists may experience increased efficiency in their research [47]. For example, before and after a manuscript is submitted for publication, it faces scrutiny from co-authors and peer reviewers who may suggest alterations to the analysis. Having a complete record of all the analysis steps, and being able to retrace those steps precisely, makes it faster and easier to implement the requested alterations [47, 102]. Reproducible practices can also improve the efficiency of team science because colleagues can more easily communicate their research protocols and inspect each other’s work; one type of relationship where this is critical is that between academic advisors and mentees [102]. Finally, when research protocols are shared transparently with the broader community, scientific advancement increases because scientists can learn more easily from each other’s work and there is less duplication of effort [102].

Reproducible practices do not necessarily ensure that others can obtain identical results to those obtained by the original scientists. Indeed, this objective may be infeasible for some types of computational analysis, including those that use randomization procedures, floating-point operations, or specialized computer hardware [85, 103]. In such cases, the goal may shift to ensuring that others can obtain results that are semantically consistent with the original findings [5, 6]. In addition, in studies where vast computational resources are needed to perform an analysis, or where data sets are distributed geographically [104–106], full reproducibility may be infeasible. Alternatively, it may be infeasible to reallocate computational resources for highly computationally intensive analyses [8]. In these cases, researchers can provide relatively simple examples to demonstrate the methodology [8]. When legal restrictions prevent researchers from publicly sharing software or data, or when software is available only via a Web interface, researchers should document the analysis steps as well as possible and describe why such components cannot be shared [25].

Computational reproducibility does not guarantee against analytical biases, or ensure that software produces scientifically valid results [107]. As with any research, a poor study design, confounding effects, or improper use of analytical software may plague even the most reproducible analyses [107, 108]. On one hand, increased transparency puts scientists at a greater risk that such problems will be exposed. On the other hand, scientists who are fully transparent about their scientific approach may be more likely to avoid such pitfalls, knowing that they will be more vulnerable to such criticisms. Either way, the scientific community benefits.

Lastly, we emphasize that some reproducibility is better than none. Although some of the practices described in this review require more technical expertise than others, they are freely accessible to all scientists, and provide long-term benefits to the researcher and to the scientific community. Indeed, as scientists act in good faith to perform these practices, where feasible, the pace of scientific progress will surely increase.

### Open Peer Review

The Open Peer Review files are available for this manuscript as Additional files – See Additional file 5.

## Additional files

Additional file 1: This script is supporting material for Fig. 1. It can be used to align DNA sequence data to a reference genome. First, it downloads the software and data files necessary for the analysis. Then, it extracts (“unzips”) these files, and aligns the data to a reference genome for Ebola virus. Finally, it converts, sorts, and indexes the aligned data. (SH 865 bytes)
Additional file 2: This Make file is supporting material for Fig. 2. It performs the same function as Additional file 1, except that it is formatted for the Make utility. Accordingly, it is structured so that specific tasks must be executed before other tasks, in a hierarchical manner
Additional file 3: This Jupyter notebook is supporting material for Fig. 3. It contains code (in the Python programming language) for generating random numbers and plotting them in a graph. (IPYNB 53 kb)
Additional file 4: This document contains code (in the R language) for generating random numbers and plotting them on a graph. This document is in R Markdown format and can be compiled using knitr. (RMD 382 bytes)
Additional file 5: Open Peer Review. (PDF 6134 kb)

## Abbreviations

DOI: Digital object identifier
HTML: Hypertext markup language
PDF: Portable document format
URL: Uniform resource locator
VCS: Version control system
