# Supplementary material for: Tools and techniques for computational reproducibility
Source: Gigascience. 2016 Jul 11;5:30. doi: 10.1186/s13742-016-0135-4 (PMC4940747; doi:10.1186/s13742-016-0135-4)
Supplement: Additional file 5: — Open Peer Review. (PDF 6134 kb) [file 13742_2016_135_MOESM5_ESM.pdf]

Review of Piccolo and Frampton manuscript

Stephen Eglen

Summary

This review provides an overview of the main tools and techniques used to ensure computational reproducibility of results. This review is a good fit to the readership of the *Gigascience* journal. Overall I found the manuscript to be clearly written and would recommend it for publication, although I would like the authors to consider the following issues in a potential revision. In particular, I am most critical about the current set of figures.

As a general comment, where possible, the material in the figures should be supported by online files (e.g. knitr, Docker) so that the reader can immediately get *workable* examples to examine, run and amend.

Detailed comments

p4, l2 (page 4, line 2): an extra citation might be (Gronenschild et al. 2012) which showed that even the choice of operating system or neuroimaging software version affects results.

p4, l18-20: as far as I am awared, journals do not encourage direct use of repositories like *github* or *bitbucket* because they offer no long-term storage. It is regarded much more appropriate to use permanent URLS (e.g. DOIs) to point to archived versions of software, such as zenodo. We have written elsewhere on this topic (Eglen et al. 2016).

p6, l10: in addition to the other topics of reproducibility and education, it would be worth mentionining education/training to encourage users to adopt reproducible practices (Wilson 2016).

p6, l15: minor point, but perhaps worth numbering the seven sections that describe the seven approaches reviewed, starting here.

p8, l17: "Make can be configured"; if you are referring to the "-j N" switch, then it is simpler to say that "Make can automatically identify. . . " as there is no extra configuration needed.

p11 l9: reference 53 at the end of the sentence is not needed, as you refer to it at the start of the sentence.

p13, l3-9: you should probably mention http://mybinder.org in this section. It provides a transparent method for interacting with Jupyter documents over the web (Rosenberg and Horn 2016).

p14, l2: I disagree slightly here with the view re: long-running jobs. *knitr* at least can cache intermediate computations transparently which helps enormously.

p14, l8: Do you have examples of Dexy in use within this field? Asking this also in a more general way, it might be worth making a table listing examples explicitly of each of the seven approaches, so that they are easy to find.

p18, l18: I do not see why the VMs are "black boxes". Surely to create the VM all the relevant code must be provided, so that you can at least examine what is done, or extend the analysis (as mentioned on l20). Can you clarify what you mean here.

p22, l1: as well as capturing software in Docker, we have used it recently to capture the entire environment to write our research papers in knitr (https://hub.docker.com/r/sje30/eglen2015/ and https://hub.docker.com/r/sje30/waverepo/).

p22, l14-19: I found the section about other operating systems (Windows/mac) rather clumsy. From the user's perspective, the modern docker toolbox seems to work smoothly enough (at least on macs) that the details at the end of p22 seem irrelevant. It might instead be worth mentioning that docker builds can be automatically triggered, e.g. upon new commits to github.

p 23, l6: "Scientific advancement requires *trust*." I think trust is the wrong word here. e.g. *The Royal Society's motto 'Nullius in verba' is taken to mean 'take nobody's word for it'* (https://royalsociety.org/about-us/history/). Rather, what these tools do is promote transparency to reduce the barriers for others to repeat prior work. With this in mind, I'd suggest the authors re-read this first paragraph of the discussion to see whether they think "trust" is what they are promoting here.

Figures

Figure 1: is this really needed in such a review? I don't think it adds anything.

Figure 2: I think should be provided as a text file so that people can run it for themselves.

Figure 3: Text file definitely needed so people can run it for themselves. But also I'd consider making the targets a bit more specific. All of the targets in this example Makefile are PHONY and perhaps it could be rewritten in the more canonical Makefile style? The way it is currently written, it is hard to see how this differs from a shell script. (Each time `make all` is run, won't the files be downloaded again?)

Figure 4: can you show something that is a bit more bio-relevant, e.g. some genomic analysis?

Figure 5: as figure 4. Can you design Figure 4 and 5 carefully to highlight the differences between knitr and jupyter?

Figure 6-8: I would suggest you drop these. They show the notion of stacks and containers, but most bioscientists won't get much value from them.

References

Eglen, Stephen, Ben Marwick, Yaroslav Halchenko, Michael Hanke, Shoaib Sufi, Padraig Gleeson, R Angus Silver, et al. 2016. "Towards Standard Practices for Sharing Computer Code and Programs in Neuroscience." *BioRxiv*. doi:10.1101/045104.

Gronenschild, Ed H B M, Petra Habets, Heidi I L Jacobs, Ron Mengelers, Nico Rozendaal, Jim van Os, and Machteld Marcelis. 2012. "The Effects of FreeSurfer Version, Workstation Type, and Macintosh Operating System Version on Anatomical Volume and Cortical Thickness Measurements." *PLoS One* 7 (6): e38234. doi:10.1371/journal.pone.0038234.

Rosenberg, David M, and Charles C Horn. 2016. "Neurophysiological Analytics for All! Free Open-Source Software Tools for Documenting, Analyzing, Visualizing, and Sharing Using Electronic Notebooks." *J. Neurophysiol.*, 20~apr, jn.00137.2016. doi:10.1152/jn.00137.2016.

Wilson, Greg. 2016. "Software Carpentry: Lessons Learned." *F1000Res.* 3 (28~jan). doi:10.12688/f1000research.3-62.v2.

GigaScience

Tools and techniques for computational reproducibility

--Manuscript Draft--

**Manuscript Number:**	
**Full Title:**	Tools and techniques for computational reproducibility
**Article Type:**	Review
**Funding Information:**	
**Abstract:**	When reporting research findings, scientists document the steps they followed so that others can verify and build upon the research. When those steps have been described in sufficient detail that others can retrace the steps and obtain similar results, the research is said to be reproducible. Computers play a vital role in many research disciplines and present both opportunities and challenges for reproducibility. Computers can be programmed to execute analysis tasks, and those programs can be repeated and shared with others. Due to the deterministic nature of most computer programs, the same analysis tasks, applied to the same data, will often produce the same outputs. However, in practice, computational findings often cannot be reproduced due to complexities in how software is packaged, installed, and executed—and due to limitations in how scientists document analysis steps. Many tools and techniques are available to help overcome these challenges. Here we describe seven such strategies. With a broad scientific audience in mind, we describe strengths and limitations of each approach, as well as circumstances under which each might be applied. No single strategy is sufficient for every scenario; thus we emphasize that it is often useful to combine approaches.
**Corresponding Author:**	Stephen R Piccolo, Ph.D.<br>Brigham Young University<br>Provo, UT UNITED STATES
**Corresponding Author Secondary Information:**	
**Corresponding Author's Institution:**	Brigham Young University
**Corresponding Author's Secondary Institution:**	
**First Author:**	Stephen R Piccolo, Ph.D.
**First Author Secondary Information:**	
**Order of Authors:**	Stephen R Piccolo, Ph.D.
	Michael B Frampton
**Order of Authors Secondary Information:**	
**Opposed Reviewers:**	
**Additional Information:**	
**Question**	**Response**
**Experimental design and statistics**<br><br>Full details of the experimental design and statistical methods used should be given in the Methods section, as detailed in our Minimum Standards Reporting Checklist. Information essential to interpreting the data presented should be made available in the figure legends.	No

Have you included all the information requested in your manuscript?	
If not, please give reasons for any omissions below.<br><br>as follow-up to "**Experimental design and statistics**<br><br>Full details of the experimental design and statistical methods used should be given in the Methods section, as detailed in our Minimum Standards Reporting Checklist. Information essential to interpreting the data presented should be made available in the figure legends.<br><br>Have you included all the information requested in your manuscript?<br><br>"	It is a review article.
**Resources**<br><br>A description of all resources used, including antibodies, cell lines, animals and software tools, with enough information to allow them to be uniquely identified, should be included in the Methods section. Authors are strongly encouraged to cite Research Resource Identifiers (RRIDs) for antibodies, model organisms and tools, where possible.<br><br>Have you included the information requested as detailed in our Minimum Standards Reporting Checklist?	No
If not, please give reasons for any omissions below.<br><br>as follow-up to "**Resources**<br><br>A description of all resources used, including antibodies, cell lines, animals and software tools, with enough information to allow them to be uniquely identified, should be included in the Methods section. Authors are strongly encouraged to cite Research Resource Identifiers (RRIDs) for antibodies, model organisms and tools, where possible.	It is a review article.

Have you included the information requested as detailed in our Minimum Standards Reporting Checklist?<br><br>"	
**Availability of data and materials**<br><br>All datasets and code on which the conclusions of the paper rely must be either included in your submission or deposited in publicly available repositories (where available and ethically appropriate), referencing such data using a unique identifier in the references and in the “Availability of Data and Materials” section of your manuscript.<br><br>Have you have met the above requirement as detailed in our Minimum Standards Reporting Checklist?	No
If not, please give reasons for any omissions below.<br><br>as follow-up to "**Availability of data and materials**<br><br>All datasets and code on which the conclusions of the paper rely must be either included in your submission or deposited in publicly available repositories (where available and ethically appropriate), referencing such data using a unique identifier in the references and in the “Availability of Data and Materials” section of your manuscript.<br><br>Have you have met the above requirement as detailed in our Minimum Standards Reporting Checklist?<br><br>"	It is a review article.

Powered by Editorial Manager® and ProduXion Manager® from Aries Systems Corporation

Manuscript

Click here to download Manuscript Piccolo_Manuscript_Gigascience.docx

Click here to view linked References

Tools and techniques for computational reproducibility

Stephen R. Piccolo[1*], Michael B. Frampton[2]

1 - Department of Biology, Brigham Young University, Provo, UT, USA

2 - Department of Computer Science, Brigham Young University, Provo, UT, USA

* Corresponding author: 4102 LSB, Brigham Young University, Provo, UT, 84602; 801-422-7116; stephen_piccolo@byu.edu

Keywords

Computational reproducibility; practice of science; literate programming; virtualization; software containers; software frameworks.

Competing Interests

None of the authors of this manuscript have any competing interests to declare.

Abstract

When reporting research findings, scientists document the steps they followed so that others can verify and build upon the research. When those steps have been described in sufficient detail that others can retrace the steps and obtain similar results, the research is said to be reproducible. Computers play a vital role in many research disciplines and present both opportunities and challenges for reproducibility. Computers can be programmed to execute analysis tasks, and those programs can be repeated and shared with others. Due to the deterministic nature of most computer programs, the same analysis tasks, applied to the same data, will often produce the same outputs. However, in practice, computational findings often cannot be reproduced due to complexities in how software is packaged, installed, and executed—and due to limitations in how scientists document analysis steps. Many tools and techniques are available to help overcome these challenges. Here we describe seven such strategies. With a broad scientific audience in mind, we describe strengths and limitations of each approach, as well as circumstances under which each might be applied. No single strategy is sufficient for every scenario; thus we emphasize that it is often useful to combine approaches.

Introduction

When reporting research, scientists document the steps they followed to obtain their results. If the description is comprehensive enough that they and others can repeat the procedures and obtain semantically consistent results, the findings are considered to be "reproducible"[1–6]. Reproducible research forms the basic building blocks of science, insofar as it allows researchers to verify and build on each other's work with confidence.

Computers play an increasingly important role in many scientific disciplines[7–10]. For example, in the United Kingdom, 92% of academic scientists use some type of software in their research, and 69% of scientists say their research is feasible only with software tools[11]. Thus efforts to increase scientific reproducibility should consider the ubiquity of computers in research.

Computers present both opportunities and challenges for scientific reproducibility. On one hand, due to the deterministic nature of most computer programs, many computational analyses can be performed such that others can obtain exactly identical results when applied to the same input data[12]. Accordingly, computational research can be held to a high reproducibility standard. On the other hand, even when no technical barrier prevents reproducibility, scientists often cannot reproduce computational findings due to complexities in how software is packaged, installed, and executed—and due to limitations in how scientists document these steps[13]. This problem is acute in many disciplines, including genomics, signal processing, and ecological modeling[14–16], where data sets are large and computational tools are evolving rapidly. However, the same problem can affect

any scientific discipline that requires computers for research. Seemingly minor differences in computational approaches can have major influences on analytical outputs[12,17–21], and the effects of these differences may exceed those that result from experimental factors[22].

Journal editors, funding agencies, governmental institutions, and individual scientists have increasingly made calls for the scientific community to embrace practices that support computational reproducibility[23–30]. This movement has been motivated, in part, by scientists' failed efforts to reproduce previously published analyses. For example, Ioannidis, et al. evaluated 18 published research studies that used computational methods to evaluate gene-expression data but were able to reproduce only 2 of those studies[31]. In many cases, a failure to share the study's data was the culprit; however, incomplete descriptions of software-based analyses were also common. Nekrutenko and Taylor examined 50 papers that analyzed next-generation sequencing data and observed that fewer than half provided any details about software versions or parameters[32]. Recreating analyses that lack such details can require hundreds of hours of effort[33] and may be impossible, even after consulting the original authors. Failure to reproduce research may also lead to careerist effects, including retractions[34].

Noting such concerns, some journals have emphasized the value of placing computer source code in open-access repositories, such as *GitHub* (https://github.com) or *BitBucket* (https://bitbucket.org). In addition, journals have extended requirements for "Methods" sections, now asking researchers to provide detailed descriptions of 1) how to install

software and its dependencies and 2) what parameters and data-preprocessing steps are used in analyses[10,23]. A recent Institute of Medicine report emphasized that, in addition to computer code and research data, "fully specified computational procedures" should be made available to the scientific community[24]. They elaborated that such procedures should include "all of the steps of computational analysis" and that "all aspects of the analysis need to be transparently reported"[24]. Such policies represent important progress. However, it is ultimately the responsibility of individual scientists to ensure that others can verify and build upon their analyses.

Describing a computational analysis sufficiently—such that others can reexecute it, validate it, and refine it—requires more than simply stating what software was used, what commands were executed, and where to find the source code[13,26,35–37]. Software is executed within the context of an operating system (for example, *Windows*, *Mac OS*, or *Linux*), which enables the software to interface with computer hardware (Figure 1). In addition, most software relies on a hierarchy of software dependencies, which perform complementary functions and must be installed alongside the main software tool. One version of a given software tool or dependency may behave differently or have a different interface than another version of the same software. In addition, most analytical software offers a range of parameters (or settings) that the user can specify. If any of these variables differs from what the original experimenter used, the software may not execute properly or analytical outputs may differ considerably from what the original experimenter observed.

Scientists can use various tools and techniques to overcome these challenges and to increase the likelihood that their computational analyses will be reproducible. These techniques range in complexity from simple (e.g., providing written documentation) to advanced (e.g., providing a "virtual" environment that includes an operating system and all software necessary to execute the analysis). This review describes seven strategies across this spectrum. We describe strengths and limitations of each approach, as well as circumstances under which each might be applied. No single strategy will be sufficient for every scenario; therefore, in many cases, it will be most practical to combine multiple approaches. This review focuses primarily on the computational aspects of reproducibility. The related topics of empirical reproducibility, statistical reproducibility, and data sharing have been described elsewhere[38–44]. We believe that with greater awareness and understanding of computational-reproducibility techniques, scientists—including those with limited computational experience—will be more apt to perform computational research in a reproducible manner.

Narrative descriptions are a simple but valuable way to support computational reproducibility

The most fundamental strategy for enabling others to reproduce a computational analysis is to provide a detailed, written description of the process. For example, when reporting computational results in a research article, authors customarily provide a narrative that describes the software they used and the analytical steps they followed. Such narratives can be invaluable in enabling others to evaluate the scientific approach and to reproduce

the findings. In many situations—for example, when software execution requires user interaction or when proprietary software is used—narratives are the only feasible option for documenting such steps. However, even when a computational analysis uses open-source software and can be fully automated, narratives help others understand how to reexecute an analysis.

Although most research articles that use computational methods provide some type of narrative, these descriptions often lack sufficient detail to enable others to retrace those steps [31,32]. Narrative descriptions should indicate the operating system(s), software dependencies, and analytical software that were used and how to obtain them. In addition, narratives should indicate the exact software versions used, the order in which they were executed, and all non-default parameters that were specified. Such descriptions should account for the fact that computer configurations differ vastly, even for computers that use the same operating system. Because it can be difficult for scientists to remember such details after the fact, it is best to record this information throughout the research process, rather than at the time of manuscript preparation[8].

The following sections describe techniques for automating computational analyses. These techniques can diminish the need for scientists to write narratives. However, because it is often impractical to automate all computational steps, we expect that, for the foreseeable future, narratives will play a vital role in enabling computational reproducibility.

Custom scripts and code can automate a research analysis

Scientific software can often be executed in an automated manner via text-based commands. Using such commands—via a *command-line interface*—scientists can indicate which software program(s) should be executed and which parameter(s) should be used. When multiple commands must be executed, they can be compiled into *scripts*, which specify the order in which the commands should be executed (Figure 2). In many cases, scripts also include commands for installing and configuring software. Such scripts serve as valuable documentation not only for individuals who wish to reexecute the analysis but also for the researcher who performed the original analysis[45]. In these cases, no amount of narrative is an adequate substitute for providing the actual commands that were used.

When writing command-line scripts, it is essential to explicitly document any software dependencies and input data that are required for each step in the analysis. The *Make* utility (https://www.gnu.org/software/make) provides one way to specify such requirements[35]. Before any command is executed, *Make* verifies that each documented dependency is available. Accordingly, researchers can use *Make* files (scripts) to specify a full hierarchy of operating-system components and dependent software that must be present to perform the analysis (Figure 3). In addition, *Make* can be configured to automatically identify any commands that can be executed in parallel, potentially reducing the amount of time required to execute the analysis. Although *Make* was designed originally for UNIX-based operating systems (such as *Mac OS* or *Linux*), similar utilities have since been developed for *Windows* operating systems

(http://gnuwin32.sourceforge.net/packages/make.htm). Box 1 lists various utilities that can be used to automate software execution.

Box 1: Utilities that can be used to automate software execution.

- *GNU Make* and *Make for Windows*: Tools for building software from source files and for ensuring that the software's dependencies are met.
- *Snakemake[46]* = An extension of Make that provides a more flexible syntax and makes it easier to execute tasks in parallel.
- *BPipe[47]* = A tool that provides a flexible syntax for users to specify commands to be executed; it maintains an audit trail of all commands that have been executed.
- *GNU Parallel[48]* = A tool for executing commands in parallel across one or more computers.
- *Makeflow[49]* = A tool that can execute commands simultaneously on various types of computer architectures, including computer clusters and cloud environments.
- *SCONS[50]* = An alternative to *GNU Make* that enables users to customize the process of building and executing software using scripts written in the Python programming language.
- *CMAKE* (https://cmake.org) = A tool that enables users to execute *Make* scripts more easily on multiple operating systems.

In addition to creating scripts that execute existing software, many researchers also create new software by writing computer code in a programming language such as Python, C++, Java, or R. Such code may perform relatively simple tasks, such as reformatting data files or invoking third-party software. In other cases, computer code may constitute a manuscript's key intellectual contribution.

Whether analysis steps are encoded in scripts or as computer code, scientists can support reproducibility by publishing these artefacts alongside research papers. By doing so, the authors enable readers to evaluate the analytical approach in full detail and to extend the analysis more readily[51]. Although scripts and code may be included alongside a manuscript as supplementary material, a better alternative is to store them in a *version-control system* (VCS)[8,9,45] and to share these repositories via Web-based services like *GitHub* (https://github.com) or *Bitbucket* (https://bitbucket.org). With such a VCS repository, scientists can track different versions of scripts and code that have been developed as the research project evolved. In addition, outside observers can see the full version history, contribute revisions to the code, and reuse the code for their own purposes[52]. When submitting a manuscript, the authors may "tag" a specific version of the repository that was used for the final analysis described in the manuscript.

Software frameworks enable easier handling of software dependencies

Virtually all computer scripts and code rely on external software dependencies and operating-system components. For example, suppose that a research study required a scientist to apply *Student's t-test*. Rather than write code that implements this statistical test, the scientist would likely find an existing software *library* that implements the test and then invoke that library from her code. A considerable amount of time can be saved with this approach, and a wide range of software libraries are freely available. However, software libraries change frequently—invoking the wrong version of a library may result in

an error or an unexpected output. Thus to enable others to reproduce an analysis, it is critical to indicate which dependencies (and versions thereof) must be installed.

One way to address this challenge is to build on a preexisting software framework. Such frameworks make it easier to access software libraries that are commonly used to perform specific types of analysis task. Typically, such frameworks also make it easier to download and install software dependencies and ensure that the versions of software libraries and their dependencies are compatible with each other. For example, *Bioconductor[53]*, created for the *R* statistical programming language[54], is a popular framework that contains hundreds of software packages for analyzing biological data[53]. The *Bioconductor* framework facilitates versioning, documenting, and distributing code. Once a software library has been incorporated into *Bioconductor*, other researchers can find, download, install, and configure it on most operating systems with relative ease. In addition, *Bioconductor* installs software dependencies automatically. These features ease the process of performing an analysis and can help with reproducibility. Various software frameworks exist for other scientific disciplines[55–60]. General-purpose tools for managing software dependencies also exist—for example, *Apache Ivy* (http://ant.apache.org/ivy) and *Puppet* (https://puppetlabs.com).

To best support reproducibility, software frameworks should make it easy for scientists to download and install previous versions of a software tool as well as previous versions of dependencies. Such a design would enable other scientists to reproduce analyses that were conducted with previous versions of a software framework. In the case of Bioconductor,

considerable extra work may be required to install specific versions of Bioconductor software and their dependencies. To overcome such limitations, scientists may use a software container or virtual machine (see below) to package the specific versions they used in an analysis. Alternatively, they might use third-party solutions such as the aRchive project (http://bioarchive.github.io).

Literate programming combines narratives directly with code

Although narratives, scripts, and computer code support reproducibility individually, additional value can be gained from combining these entities. Even though a researcher may provide computer code alongside a research paper, other scientists may have difficulty interpreting how the code accomplishes specific tasks. A longstanding way to address this problem is via code comments, which are human-readable annotations interspersed throughout computer code. However, code comments and other types of documentation often become outdated as code evolves throughout the analysis process[61]. One way to overcome this problem is to use a technique called *literate programming[62]*. With this approach, the scientist writes a narrative of the scientific analysis and intermingles code directly within the narrative. As the code is executed, a document is generated that includes the code, narratives, and any outputs (e.g., figures, tables) that the code produces. Accordingly, literate programming helps ensure that readers understand exactly how a particular research result was obtained. In addition, this approach motivates the scientist to keep the target audience in mind when performing a computational analysis, rather than simply to write code that a computer can parse[62]. Consequently, by reducing barriers of

understanding among scientists, literate programming can help to engender greater trust in computational findings.

One popular literate-programming tool is *Jupyter[63]*. Using its Web-based interface, scientists can create interactive "notebooks" that combine code, data, mathematical equations, plots, and rich media[64]. Originally known as *IPython* and previously designed exclusively for the *Python* programming language, *Jupyter* (http://jupyter.org) now makes it possible to execute code in many different programming languages. Such functionality may be important to scientists who prefer to combine the strengths of different programming languages.

knitr[65] has also gained considerable popularity as a literate-programming tool. It is written in the *R* programming language and thus can be integrated seamlessly with the array of statistical and plotting tools available in that environment. However, like *Jupyter*, *knitr* can execute code written in multiple programming languages. Commonly, *knitr* is applied to documents that have been authored using *RStudio[66]*, an open-source tool with advanced editing and package-management features.

Jupyter notebooks and *knitr* reports can be saved in various output formats, including HTML and PDF (see examples in Figures 4-5). Increasingly, scientists include such documents with journal manuscripts as supplementary material, enabling others to repeat analysis steps and recreate manuscript figures[67–70].

Scientists typically use literate-programming tools for data analysis tasks that can be executed in a modest amount of time (e.g., minutes or hours). It is possible to execute

Jupyter or *knitr* at the command line; thus longer-running tasks can be executed on high-performance computers. However, this approach runs counter to the interactive nature of notebooks and require additional technical expertise to configure and execute the notebooks.

Literate-programming notebooks are suitable for research analyses that require a modest amount of computer code. For analyses that require larger amounts of code, more advanced programming environments may be more suitable—perhaps in combination with a "literate documentation" tool such as Dexy (http://www.dexy.it).

Workflow-management systems enable software execution via a graphical user interface

Writing computer scripts and code may seem daunting to many researchers. Although various courses and tutorials are helping to make this task less formidable[71–74], many scientists use "workflow management systems" to facilitate the process of executing scientific software[75]. Typically managed via a graphical user interface, workflow management systems enable scientists to upload data and process it using existing tools. For multistep analyses, the output from one tool can be used as input to additional tools, potentially resulting in a series of commands known as a workflow.

Galaxy[76,77] has gained considerable popularity within the bioinformatics community—especially for performing next-generation sequencing analysis. As users construct workflows, *Galaxy* provides descriptions of how software parameters should be used,

examples of how input files should be formatted, and links to relevant discussion forums. To help with processing large data sets and computationally complex algorithms, *Galaxy* also provides an option to execute workflows on cloud-computing services[78]. In addition, researchers can share workflows with each other (https://usegalaxy.org); this feature has enabled the *Galaxy* team to build a community that helps to encourage reproducibility, define best practices, and reduce the time required for novices to get started.

Various other workflow systems are freely available to the research community (see Box 2). For example, *VisTrails* is used by researchers from many disciplines, including climate science, microbial ecology, and quantum mechanics[79]. It enables scientists to design workflows visually, connecting data inputs with analytical modules and the resulting outputs. In addition, *VisTrails* tracks a full history of how each workflow was created. This capability, referred to as "retrospective provenance", makes it possible for others not only to reproduce the final version of an analysis but also to examine previous incarnations of the workflow and examine how each change influenced the analytical outputs[80].

Box 2: Workflow management tools freely available to the research community.

- Galaxy[76,77] - https://usegalaxy.org
- VisTrails[79] - http://www.vistrails.org
- Kepler[81] - https://kepler-project.org
- iPlant Collaborative[82] - http://www.iplantcollaborative.org
- GenePattern[83,84] - http://www.broadinstitute.org/cancer/software/genepattern
- Taverna[85] - http://www.taverna.org.uk
- LONI Pipeline[86] - http://pipeline.bmap.ucla.edu

Although workflow-management systems offer many advantages, users must accept tradeoffs. For example, although the teams that develop these tools often provide public servers where users can execute workflows, many scientists share these limited resources, so the public servers may not have adequate computational power or storage space to execute large-scale analyses in a timely manner. As an alternative, many scientists install these systems on their own computers; however, configuring and supporting them requires time and expertise. In addition, if a workflow tool does not yet provide a module to support a given analysis, the scientist must create a new module to support it. This task constitutes additional overhead; however, utilities such as the *Galaxy Tool Shed[87]* are helping to facilitate this process.

Virtual machines encapsulate an entire operating system and software dependencies

Whether an analysis is executed at the command line, within a literate-programming notebook, or via a workflow-management system, an operating system and relevant software dependencies must be installed before the analysis can be performed. The process of identifying, installing, and configuring such dependencies consumes a considerable amount of scientists' time. Different operating systems (and versions thereof) may require different installation and configuration steps. Furthermore, earlier versions of software dependencies, which may currently be installed on a given computer, may be incompatible with—or produce different outputs than—newer versions.

One solution is to use virtual machines, which can encapsulate an entire operating system and all software, scripts, code, and data necessary to execute a computational analysis[88,89] (Figure 6). Using virtualization software—such as *VirtualBox* or *VMWare* (see Box 3)—a virtual machine can be executed on practically any desktop, laptop, or server, irrespective of the main ("host") operating system on the computer. For example, even though a scientist's computer may be running a *Windows* operating system, the scientist may perform an analysis on a *Linux* operating system that is running concurrently—within a virtual machine—on the same computer. The scientist has full control over the virtual ("guest") operating system and thus can install software and modify configuration settings as necessary. In addition, a virtual machine can be constrained to use specific amounts of computational resources (e.g., computer memory, processing power), thus enabling system administrators to ensure that multiple virtual machines can be executed simultaneously on the same computer without impacting each other's performance. After executing an analysis, the scientist can export the entire virtual machine to a single, binary file. Other scientists can then use this file to reconstitute the same computational environment that was used for the original analysis. With a few exceptions (see Discussion), these scientists will obtain exactly the same results that the original scientist obtained. This process provides the added benefits that 1) the scientist must only document the installation and configuration steps for a single operating system, 2) other scientists need only install the virtualization software and not individual software components, and 3) analyses can be reexecuted indefinitely, so long as the virtualization software remains compatible with current computer systems[90]. Also useful, a team of

scientists can employ virtual machines to ensure that each team member has the same computational environment, even though the team members may have different configurations on their host operating systems.

One criticism of using virtual machines to support computational reproducibility is that virtual-machine files are large (typically multiple gigabytes), especially if they include raw data files. This imposes a barrier for researchers to share virtual machines with the research community. One option is to use cloud-computing services (see Box 4). Scientists can execute an analysis in the cloud, take a "snapshot" of their virtual machine, and share it with others in that environment[88,91]. Cloud-based services typically provide repositories where virtual-machine files can be stored and shared easily among users. Despite these advantages, some researchers may prefer that their data reside on local computers, rather than in the cloud—at least while the research is being performed. In addition, cloud-based services may use proprietary software, so virtual machines may only be executable within each provider's infrastructure. Furthermore, to use a cloud-service provider, scientists may need to activate a fee-based account.

Another criticism of using virtual machines to support computational reproducibility is that the software and scripts used in the analysis will be less easily accessible to other scientists—details of the analysis are effectively concealed behind a “black box”[92]. Although other researchers may be able to reexecute the analysis within the virtual machine, it may be more difficult for them to understand and extend the analysis[92]. This problem can be ameliorated when all narratives, scripts, and code are stored in public

repositories—separately from the virtual machine—and then imported when the analysis is executed[93]. Another solution is to use a prepackaged virtual machine, such as *Cloud BioLinux*, that contains a variety of software tools commonly used within a given research community[94].

Scientists can automate the process of building and configuring virtual machines using tools such as *Vagrant* or *Vortex* (see Box 3). For either tool, users can write text-based configuration files that provide instructions for building virtual machines and allocating computational resources to them. In addition, these configuration files can be used to specify analysis steps[93]. Because these files are text based and relatively small (usually a few kilobytes), scientists can share them easily and track different versions of the files via source-control repositories. This approach also mitigates problems that might arise during the analysis stage. For example, even when a computer's *host* operating system must be reinstalled due to a computer hardware failure, the virtual machine can be recreated with relative ease.

Box 3: Virtual-machine software.

Virtualization hypervisors:

- VirtualBox (open source) - https://www.virtualbox.org
- Xen (open source) - http://www.xenproject.org
- VMWare (partially open source) - http://www.vmware.com

Virtual-machine management tools:

- Vagrant (open source) - https://www.vagrantup.com
- Vortex (open source) - https://github.com/websecurify/node-vortex

Box 4: Commercial cloud-service providers.

- Amazon Web Services - http://aws.amazon.com
- Rackspace Cloud - http://www.rackspace.com/cloud
- Google Cloud Platform - https://cloud.google.com/compute
- Windows Azure - https://azure.microsoft.com

Software containers ease the process of installing and configuring dependencies

Software containers are a lighter-weight alternative to virtual machines. Like virtual machines, containers can encapsulate operating-system components, scripts, code, and data into a single package that can be shared with others. Thus, as with virtual machines, analyses executed within a software container should produce identical outputs, irrespective of the underlying operating system or whatever software may be installed outside the container (see Discussion for caveats). As is true for virtual machines, multiple containers can be executed simultaneously on a single computer, and each container may contain different software versions and configurations. However, whereas virtual machines include an entire operating system, software containers interface directly with the computer's main operating system and extend it as needed (Figure 3). This design provides less flexibility than virtual machines because containers are specific to a given type of operating system; however, containers require considerably less computational overhead than virtual machines and can be initialized much more quickly[95].

The open-source *Docker* utility (https://www.docker.com)—which has gained popularity among informaticians since its release in 2013—provides the ability to build, execute, and share software containers for Linux-based operating systems. Users specify a *Docker* container's contents using text-based commands. These instructions can be placed in a "Dockerfile," which other scientists can use to rebuild the container. As with virtual-machine configuration files, Dockerfiles are text based, so they can be shared easily and can be tracked and versioned in source-control repositories. Once a *Docker* container has been built, its contents can be exported to a binary file; these files are generally smaller than virtual-machine files, so they can be shared more easily—for example, via *DockerHub* (https://hub.docker.com).

A key feature of *Docker* containers is that their contents can be stacked in distinct layers (or "images"). Each image includes software component(s) that address a particular need (see Figure 7 for an example). Within a given research lab, scientists might create general-purpose images that support functionality for multiple projects, and they might create specialized images that address the needs of specific projects. *Docker's* modular design provides the advantage that when images within a container are updated, *Docker* only needs to track the specific components that have changed; users who wish to update to a newer version must download a relatively small update. In contrast, even a minor change to a virtual machine would require users to export and reshare the entire virtual machine.

Scientists have begun to share *Docker* images with others who are working in the same subdiscipline. For example, *nucleotid.es* is a catalog of genome-assembly tools that have

been encapsulated in *Docker* images[96,97]. Genome-assembly tools differ considerably in the dependencies that they require and in the parameters that they support. This project provides a means to standardize these assemblers, to circumvent the need to install dependencies for each tool, and to perform benchmarks across the tools. Such projects may help to reduce the reproducibility burden on individual scientists.

The use of *Docker* containers for reproducible research comes with caveats. Individual containers are stored and executed in isolation from other containers on the same computer; however, because all containers on a given machine share the same operating system, this isolation is not as complete as it is with virtual machines. This means, for example, that a given container is not guaranteed to have access to a specific amount of computer memory or processing power—multiple containers may have to compete for these resources[95]. In addition, containers may be more vulnerable to security breaches[95]. Another caveat is that *Docker* containers can only be executed on Linux-based operating systems. For other operating systems, *Docker* containers must be executed within a virtual machine (for example, see http://boot2docker.io). Although this configuration offsets some benefits of using containers, combining virtual machines with containers may provide a happy medium for many scientists, allowing them to use a non-Linux *host* operating system, while receiving the benefits of containers within the *guest* operating system.

Efforts are ongoing to develop and refine software-container technologies. Box 5 lists various tools that are currently available. In coming years, these technologies promise to play an influential role within the scientific community.

Box 5: Open-source containerization software.

- Docker - https://www.docker.com
- Linux Containers - https://linuxcontainers.org
- lmctfy - https://github.com/google/lmctfy
- OpenVZ - http://openvz.org
- Warden - http://docs.cloudfoundry.org/concepts/architecture/warden.html

Discussion

Scientific advancement requires trust. This review provides a comprehensive, though inexhaustive, list of techniques that can help to engender such trust. Principally, scientists must perform research in such ways that they can trust their own findings[3,45]. Science philosopher Karl Popper contended that "[w]e do not take even our own observations quite seriously, or accept them as scientific observations, until we have repeated and tested them"[2]. Indeed, in many cases, the individuals who benefit most from computational reproducibility are those who performed the original analysis. But reproducible practices can also help scientists garner each other's trust[45,98]. When other scientists can reproduce an analysis and determine exactly how its conclusions were drawn, they may be more apt to cite the work and build upon it. In contrast, when others fail to reproduce

research findings, it can derail scientific progress and lead to embarrassment, accusations, and retractions.

We have described seven tools and techniques for computational reproducibility. None of these approaches is sufficient for every scenario in isolation. Rather scientists will often find value in combining approaches. For example, a researcher who uses a literate-programming notebook (which combines narratives with code) might incorporate the notebook into a software container so that others can execute it without needing to install specific software dependencies. The container might also include a workflow-management system to ease the process of integrating multiple tools and incorporating best practices for the analysis. This container could be packaged within a virtual machine to ensure that it can be executed on many operating systems (see Figure 8). In determining a reproducibility strategy, scientists must evaluate the tradeoff between robustness and practicality.

The call for computational reproducibility relies on the premise that reproducible science will bolster the efficiency of the overall scientific enterprise[99]. Although reproducible practices may require additional time and effort, these practices provide ancillary benefits that help offset those expenditures[45]. Primarily, the scientists who perform a study may experience increased efficiency[45]. For example, before and after a manuscript is submitted for publication, it faces scrutiny from co-authors and peer reviewers who may suggest alterations to the analysis. Having a complete record of all analysis steps and being able to retrace those steps precisely, makes it faster and easier to implement the requested

alterations[45,100]. Reproducible practices can also improve the efficiency of team science because colleagues can more easily communicate their research protocols and inspect each other's work; one type of relationship where this is critical is that between academic advisors and mentees[100]. Finally, when research protocols are shared transparently with the broader community, scientific advancement increases because scientists can learn more easily from each other's work and duplicate each other's efforts less frequently[100].

Reproducible practices do not necessarily ensure that others can obtain results that are perfectly identical to what the original scientists obtained. Indeed, this objective may be infeasible for some types of computational analysis, including those that use randomization procedures, floating-point operations, or specialized computer hardware[89,101]. In such cases, the goal may shift to ensuring that others can obtain results that are semantically consistent with the original findings[5,6]. In addition, in studies where vast computational resources are needed to perform an analysis or where data sets are distributed geographically[102–104], full reproducibility may be infeasible. Alternatively, it may be infeasible to reallocate computational resources for analyses that are highly computationally intensive[8]. In these cases, researchers can provide relatively simple examples that demonstrate the methodology[8]. When legal restrictions prevent researchers from sharing software or data publicly, or when software is available only via a Web interface, researchers should document the analysis steps as well as possible and describe why such components cannot be shared[24].

Computational reproducibility does not guarantee against analytical biases or ensure that software produces scientifically valid results[105]. As with any research, a poor study design, confounding effects, or improper use of analytical software may plague even the most reproducible analyses[105,106]. On one hand, increased transparency puts scientists at a greater risk that such problems will be exposed. On the other hand, scientists who are fully transparent about their scientific approach may be more likely to avoid such pitfalls, knowing that they will be more vulnerable to such criticisms. Either way, the scientific community benefits.

Lastly, we emphasize that some reproducibility is better than none. As Voltaire said, the perfect should not be the enemy of the good[107]. Although some of the practices described in this review require more technical expertise than others, these practices are freely accessible to all scientists and provide long-term benefits to the researcher and to the scientific community. Indeed, as scientists act in good faith to perform these practices, where feasible, the pace of scientific progress will surely increase.

Figure Legends

Figure 1: Basic computer architecture. Computer hardware consists of hardware devices, including central processing units, hard drives, random access memory, keyboard, mouse, etc. Operating systems enable software to interface with hardware; popular operating-system families are *Windows*, *Mac OS*, and *Linux*. Users interact with computers via software interfaces. In scientific computing, software enables users to execute algorithms, analyze data, generate graphics, etc. To execute properly, most software tools depend on specific versions of software dependencies, which must be installed on the same operating system.

Figure 2: Example of a command-line script. This script can be used to align DNA sequence data to a reference genome. First it downloads software and data files necessary for the analysis. Then it extracts ("unzips") these files, aligns the data to a reference genome for Ebolavirus. Finally, it converts, sorts, and indexes the aligned data.

Figure 3: Example of a *Make* file. This file performs the same function as the command-line script shown in Figure 2, except that it is formatted for the *Make* utility. Accordingly, it is structured so that specific tasks must be executed before other tasks, in a hierarchical structure. For example, the "download" target must be completed before the downloaded files can be prepared ("prepare" target). The "all" target specifies all of the targets that must be executed, in order.

Figure 4: Example of a *Jupyter* notebook. This example contains code (in the Python programming language) for generating random numbers and plotting them in a graph within a *Jupyter* notebook. Importantly, the code and output object (graph) are contained within the same document.

Figure 5: Example of a document that has been created using *knitr*. This example contains code (in the R language) for generating random numbers and plotting them in a graph. The *knitr* tool was used to generate the document, which combines the code and the output object (figure).

Figure 6: Architecture of virtual machines. Virtual machines encapsulate analytical software and dependencies within a "guest" operating system, which may be different than the main ("host") operating system. A virtual machine executes in the context of virtualization software, which executes alongside whatever other software is installed on the computer.

Figure 7: Architecture of software containers. Software containers encapsulate analytical software and dependencies. In contrast to virtual machines, containers execute within the context of the computer's main operating system.

Figure 8: Example of a *Docker* container that could be used for genomics research. This container would enable researchers to preprocess various types of molecular data, using tools from *Bioconductor* and *Galaxy*, and to analyze the resulting data within an *IPython* notebook. Each box within the container represents a distinct *Docker* image. These images are layered such that some images depend on others (for example, the *Bioconductor*

image depends on R). At its base, the container includes operating-system libraries, which may not be present (or may be configured differently) on the computer's main operating system.

References

1. Fisher RA. The Design of Experiments. New York: Hafner Press; 1935.

2. Popper KR. 2002. The logic of scientific discovery. London Routledge; 1959.

3. Peng RD. Reproducible research in computational science. Science. 2011;334:1226–7.

4. Russell JF. If a job is worth doing, it is worth doing twice. Nature. 2013;496:7.

5. Feynman RP, Leighton RB, Sands M. Six Easy Pieces: Essentials of Physics Explained by Its Most Brilliant Teacher. Perseus Books; 1994. p. 34–5.

6. Murray-Rust P, Murray-Rust D. Reproducible Physical Science and the Declaratron. In: Stodden VC, Leisch F, Peng RD, editors. Implementing Reproducible Research. CRC Press; 2014. p. 113.

7. Hey AJG, Tansley S, Tolle KM, Others. The fourth paradigm: data-intensive scientific discovery. Microsoft Research Redmond, WA; 2009.

8. Millman KJ, Pérez F. Developing Open-Source Scientific Practice. Implementing Reproducible Research. CRC Press; 2014;149.

9. Wilson G, Aruliah DA, Brown CT, Chue Hong NP, Davis M, Guy RT, et al. Best practices for scientific computing. PLoS Biol. 2014;12:e1001745.

10. Software with impact. Nat. Methods. 2014;11:211.

11. Hong NC. We are the 92% [Internet]. Figshare; 2014. Available from: http://dx.doi.org/10.6084/M9.FIGSHARE.1243288

12. Sacks J, Welch WJ, Mitchell TJ, Wynn HP. Design and Analysis of Computer Experiments. Stat. Sci. Institute of Mathematical Statistics; 1989;4:409–23.

13. Garijo D, Kinnings S, Xie L, Xie L, Zhang Y, Bourne PE, et al. Quantifying reproducibility in computational biology: the case of the tuberculosis drugome. PLoS One. 2013;8:e80278.

14. Error prone. Nature. 2012;487:406.

15. Vandewalle P, Barrenetxea G, Jovanovic I, Ridolfi A, Vetterli M. Experiences with Reproducible Research in Various Facets of Signal Processing Research. 2007 IEEE International Conference on Acoustics, Speech and Signal Processing - ICASSP ’07. IEEE; 2007. p. IV – 1253 – IV – 1256.

16. Cassey P, Cassey P, Blackburn T, Blackburn T. Reproducibility and Repeatability in Ecology. Bioscience. 2006;56:958–9.

17. Murphy JM, Sexton DMH, Barnett DN, Jones GS, Webb MJ, Collins M, et al. Quantification of modelling uncertainties in a large ensemble of climate change simulations. Nature. 2004;430:768–

72.

18. McCarthy DJ, Humburg P, Kanapin A, Rivas MA, Gaulton K, Cazier J-B, et al. Choice of transcripts and software has a large effect on variant annotation. Genome Med. 2014;6:26.

19. Neuman JA, Isakov O, Shomron N. Analysis of insertion-deletion from deep-sequencing data: Software evaluation for optimal detection. Brief. Bioinform. 2013;14:46–55.

20. Bradnam KR, Fass JN, Alexandrov A, Baranay P, Bechner M, Birol I, et al. Assemblathon 2: evaluating de novo methods of genome assembly in three vertebrate species. Gigascience. 2013;2:10.

21. Bilal E, Dutkowski J, Guinney J, Jang IS, Logsdon BA, Pandey G, et al. Improving Breast Cancer Survival Analysis through Competition-Based Multidimensional Modeling. PLoS Comput. Biol. 2013;9:e1003047.

22. Moskvin OV, McIlwain S, Ong IM. CAMDA 2014: Making sense of RNA-Seq data: From low-level processing to functional analysis. Systems Biomedicine. 2014;2:31–40.

23. Reducing our irreproducibility. Nature. 2013;496:398–398.

24. Michael CM, Nass SJ, Omenn GS, editors. Evolution of Translational Omics: Lessons Learned and the Path Forward. Washington, D.C.: The National Academies Press; 2012.

25. Collins FS, Tabak L a. Policy: NIH plans to enhance reproducibility. Nature. 2014;505:612–3.

26. Chambers JM. S as a Programming Environment for Data Analysis and Graphics. Problem Solving Environments for Scientific Computing, Proc. 17th Symp. on the Interface of Stat. and Comp. North Holland; 1985. p. 211–4.

27. LeVeque RJ, Mitchell IM, Stodden V. Reproducible research for scientific computing: Tools and strategies for changing the culture. Computing in Science and Engineering. 2012;14:13.

28. Stodden V, Guo P, Ma Z. Toward Reproducible Computational Research: An Empirical Analysis of Data and Code Policy Adoption by Journals. PLoS One. 2013;8:2–9.

29. Morin A, Urban J, Adams PD, Foster I, Sali A, Baker D, et al. Research priorities. Shining light into black boxes. Science. 2012;336:159–60.

30. Rebooting review. Nat. Biotechnol. 2015;33:319.

31. Ioannidis JP a., Allison DB, Ball C a., Coulibaly I, Cui X, Culhane AC, et al. Repeatability of published microarray gene expression analyses. Nat. Genet. 2009;41:149–55.

32. Nekrutenko A, Taylor J. Next-generation sequencing data interpretation: enhancing reproducibility and accessibility. Nat. Rev. Genet. Nature Publishing Group; 2012;13:667–72.

33. Baggerly K a., Coombes KR. Deriving chemosensitivity from cell lines: Forensic bioinformatics and reproducible research in high-throughput biology. Ann. Appl. Stat. 2009;3:1309–34.

34. Decullier E, Huot L, Samson G, Maisonneuve H. Visibility of retractions: a cross-sectional one-year study. BMC Res. Notes. 2013;6:238.

35. Claerbout JF, Karrenbach M. Electronic Documents Give Reproducible Research a New Meaning. Meeting of the Society of Exploration Geophysics. New Orleans, LA; 1992.

36. Stodden V, Miguez S. Best Practices for Computational Science: Software Infrastructure and Environments for Reproducible and Extensible Research. Journal of Open Research Software. 2014;2:21.

37. Ravel J, Wommack KE. All hail reproducibility in microbiome research. Microbiome. 2014;2:8.

38. Stodden V. 2014: What scientific idea is ready for retirement? [Internet]. http://edge.org/response-detail/25340. 2014. Available from: http://edge.org/response-detail/25340

39. Birney E, Hudson TJ, Green ED, Gunter C, Eddy S, Rogers J, et al. Prepublication data sharing. Nature. 2009;461:168–70.

40. Hothorn T, Leisch F. Case studies in reproducibility. Brief. Bioinform. 2011;12:288–300.

41. Schofield PN, Bubela T, Weaver T, Portilla L, Brown SD, Hancock JM, et al. Post-publication sharing of data and tools. Nature. 2009;461:171–3.

42. Piwowar H a., Day RS, Fridsma DB. Sharing detailed research data is associated with increased citation rate. PLoS One [Internet]. 2007;2. Available from: http://dx.doi.org/10.1371/journal.pone.0000308

43. Johnson VE. Revised standards for statistical evidence. Proc. Natl. Acad. Sci. U. S. A. 2013;110:19313–7.

44. Halsey LG, Curran-everett D, Vowler SL, Drummond GB. The fickle P value generates irreproducible results. Nat. Methods. 2015;12:179–85.

45. Sandve GK, Nekrutenko A, Taylor J, Hovig E. Ten Simple Rules for Reproducible Computational Research. PLoS Comput. Biol. 2013;9:1–4.

46. Köster J, Rahmann S. Snakemake--a scalable bioinformatics workflow engine. Bioinformatics. 2012;28:2520–2.

47. Sadedin SP, Pope B, Oshlack A. Bpipe : A Tool for Running and Managing Bioinformatics Pipelines. Bioinformatics. 2012;28:1525–6.

48. Tange O. GNU Parallel - The Command-Line Power Tool. ;login: The USENIX Magazine. Frederiksberg, Denmark; 2011;36:42–7.

49. Albrecht M, Donnelly P, Bui P, Thain D. Makeflow: A portable abstraction for data intensive computing on clusters, clouds, and grids. Proceedings of the 1st ACM SIGMOD Workshop on Scalable Workflow Execution Engines and Technologies. 2012.

50. Knight S, Austin C, Crain C, Leblanc S, Roach A. Scons software construction tool [Internet]. 2011. Available from: http://www.scons.org

51. Code share. Nature. 2014;514:536.

52. Loeliger J, McCullough M. Version Control with Git: Powerful Tools and Techniques for Collaborative Software Development. "O'Reilly Media, Inc."; 2012. p. 456.

53. Huber W, Carey VJ, Gentleman R, Anders S, Carlson M, Carvalho BS, et al. Orchestrating high-throughput genomic analysis with Bioconductor. Nat. Methods. Nature Publishing Group; 2015;12:115–21.

54. R Core Team. R: A Language and Environment for Statistical Computing [Internet]. Vienna, Austria: R Foundation for Statistical Computing; 2014. Available from: http://www.r-project.org

55. Tóth G, Sokolov IV, Gombosi TI, Chesney DR, Clauer CR, De Zeeuw DL, et al. Space Weather Modeling Framework: A new tool for the space science community. J. Geophys. Res. 2005;110:A12226.

56. Tan E, Choi E, Thoutireddy P, Gurnis M, Aivazis M. GeoFramework: Coupling multiple models of mantle convection within a computational framework. Geochem. Geophys. Geosyst. [Internet]. 2006;7. Available from: http://doi.wiley.com/10.1029/2005GC001155

57. Heisen B, Boukhelef D, Esenov S, Hauf S, Kozlova I, Maia L, et al. Karabo: An Integrated Software Framework Combining Control, Data Management, and Scientific Computing Tasks. 14th International Conference on Accelerator & Large Experimental Physics Control Systems, ICALEPCS2013. San Francisco, CA; 2013.

58. Schneider CA, Rasband WS, Eliceiri KW. NIH Image to ImageJ: 25 years of image analysis. Nat. Methods. Nature Publishing Group; 2012;9:671–5.

59. Schindelin J, Arganda-Carreras I, Frise E, Kaynig V, Longair M, Pietzsch T, et al. Fiji: an open-source platform for biological-image analysis. Nat. Methods. Nature Publishing Group, a division of Macmillan Publishers Limited. All Rights Reserved.; 2012;9:676–82.

60. Biasini M, Schmidt T, Bienert S, Mariani V, Studer G, Haas J, et al. OpenStructure: an integrated software framework for computational structural biology. Acta Crystallogr. D Biol. Crystallogr. 2013;69:701–9.

61. Martin RC. Clean code: a handbook of agile software craftsmanship. Pearson Education; 2009.

62. Knuth DE. Literate Programming. Comput. J. 1984;27:97–111.

63. Pérez F, Granger BE. IPython: a System for Interactive Scientific Computing. Computing in Science and Engineering. IEEE Computer Society; 2007;9:21–9.

64. Shen H. Interactive notebooks: Sharing the code. Nature. 2014;515:151–2.

65. Xie Y. Dynamic Documents with R and knitr. CRC Press; 2013. p. 216.

66. RStudio Team. RStudio: Integrated Development for R [Internet]. [cited 2015 Nov 20]. Available from: http://www.rstudio.com

67. Gross AM, Orosco RK, Shen JP, Egloff AM, Carter H, Hofree M, et al. Multi-tiered genomic analysis of head and neck cancer ties TP53 mutation to 3p loss. Nat. Genet. Nature Publishing Group; 2014;46:1–7.

68. Ding T, Schloss PD. Dynamics and associations of microbial community types across the human body. Nature. Nature Publishing Group, a division of Macmillan Publishers Limited. All Rights Reserved.; 2014;509:357–60.

69. Ram Y, Hadany L. The probability of improvement in Fisher's geometric model: A probabilistic approach. Theor. Popul. Biol. 2015;99:1–6.

70. Meadow JF, Altrichter AE, Kembel SW, Moriyama M, O'Connor TK, Womack AM, et al. Bacterial communities on classroom surfaces vary with human contact. Microbiome. 2014;2:7.

71. White E. Programming for Biologists [Internet]. Available from: http://www.programmingforbiologists.org

72. Wilson G. Software Carpentry: lessons learned. F1000Res. 2014;3:62.

73. Peng RD. Coursera course: Computing for Data Analysis [Internet]. Available from: https://www.coursera.org/course/compdata

74. Bioconductor - Courses and Conferences [Internet]. [cited 2015 Nov 20]. Available from: http://master.bioconductor.org/help/course-materials

75. Gil Y, Deelman E, Ellisman M, Fahringer T, Fox G, Gannon D, et al. Examining the challenges of scientific workflows. Computer . 2007;40:24–32.

76. Giardine B, Riemer C, Hardison RC, Burhans R, Elnitski L, Shah P, et al. Galaxy: a platform for interactive large-scale genome analysis. Genome Res. 2005;15:1451–5.

77. Goecks J, Nekrutenko A, Taylor J. Galaxy: a comprehensive approach for supporting accessible, reproducible, and transparent computational research in the life sciences. Genome Biol. 2010;11:R86.

78. Afgan E, Baker D, Coraor N, Goto H, Paul IM, Makova KD, et al. Harnessing cloud computing with Galaxy Cloud. Nat. Biotechnol. 2011;29:972–4.

79. Callahan SP, Freire J, Santos E, Scheidegger CE, Silva CT, Vo HT. VisTrails: Visualization Meets Data Management. Proceedings of the 2006 ACM SIGMOD International Conference on Management of Data. New York, NY, USA: ACM; 2006. p. 745–7.

80. Davidson SB, Freire J. Provenance and scientific workflows. Proceedings of the 2008 ACM SIGMOD international conference on Management of data - SIGMOD '08. 2008. p. 1345.

81. Altintas I, Berkley C, Jaeger E, Jones M, Ludascher B, Mock S. Kepler: an extensible system for design and execution of scientific workflows. Proceedings. 16th International Conference on

Scientific and Statistical Database Management, 2004. IEEE; 2004. p. 423–4.

82. Goff SA, Vaughn M, McKay S, Lyons E, Stapleton AE, Gessler D, et al. The iPlant Collaborative: Cyberinfrastructure for Plant Biology. Front. Plant Sci. Frontiers; 2011;2:34.

83. Reich M, Liefeld T, Gould J, Lerner J, Tamayo P, Mesirov JP. GenePattern 2.0. Nat. Genet. 2006;38:500–1.

84. Reich M, Liefeld J, Thorvaldsdottir H, Ocana M, Polk E, Jang D, et al. GenomeSpace: An environment for frictionless bioinformatics. Cancer Res. 2012;72:3966–3966.

85. Wolstencroft K, Haines R, Fellows D, Williams A, Withers D, Owen S, et al. The Taverna workflow suite: designing and executing workflows of Web Services on the desktop, web or in the cloud. Nucleic Acids Res. 2013;41:557–61.

86. Rex DE, Ma JQ, Toga AW. The LONI Pipeline Processing Environment. Neuroimage. 2003;19:1033–48.

87. Lazarus R, Kaspi A, Ziemann M. Creating re-usable tools from scripts: The Galaxy Tool Factory. Bioinformatics. 2012;28:3139–40.

88. Dudley JT, Butte AJ. In silico research in the era of cloud computing. Nat. Biotechnol. Nature Publishing Group; 2010;28:1181–5.

89. Hurley DG, Budden DM, Crampin EJ. Virtual Reference Environments: a simple way to make research reproducible. Brief. Bioinform. 2014;1–3.

90. Gent IP. The Recomputation Manifesto. arXiv [Internet]. 2013; Available from: http://arxiv.org/abs/1304.3674

91. Howe B. Virtual Appliances, Cloud Computing, and Reproducible Research. Comput. Sci. Eng. IEEE Computer Society; 2012;14:36–41.

92. Brown CT. Virtual machines considered harmful for reproducibility [Internet]. 2012. Available from: http://ivory.idyll.org/blog/vms-considered-harmful.html

93. Piccolo SR. Building portable analytical environments to improve sustainability of computational-analysis pipelines in the sciences [Internet]. 2014. Available from: http://dx.doi.org/10.6084/m9.figshare.1112571

94. Krampis K, Booth T, Chapman B, Tiwari B, Bicak M, Field D, et al. Cloud BioLinux: pre-configured and on-demand bioinformatics computing for the genomics community. BMC Bioinformatics. BioMed Central Ltd; 2012;13:42.

95. Felter W, Ferreira A, Rajamony R, Rubio J. An Updated Performance Comparison of Virtual Machines and Linux Containers [Internet]. IBM Research Division; 2014. Available from: http://domino.research.ibm.com/library/CyberDig.nsf/papers/0929052195DD819C85257D2300681E7B/$File/rc25482.pdf

96. Belmann P, Dröge J, Bremges A, McHardy AC, Sczyrba A, Barton MD. Bioboxes: standardised

containers for interchangeable bioinformatics software. Gigascience. 2015;4:47.

97. Barton M. nucleotides · genome assembler benchmarking [Internet]. [cited 2015 Nov 20]. Available from: http://nucleotid.es

98. Hones MJ. Reproducibility as a Methodological Imperative in Experimental Research. PSA: Proceedings of the Biennial Meeting of the Philosophy of Science Association. Philosophy of Science Association; 1990. p. 585–99.

99. Crick T. " Share and Enjoy ": Publishing Useful and Usable Scientific Models. Available from: http://arxiv.org/abs/1409.0367v2

100. Donoho DL. An invitation to reproducible computational research. Biostatistics. 2010;11:385–8.

101. Goldberg D. What Every Computer Scientist Should Know About Floating-point Arithmetic. ACM Comput. Surv. New York, NY, USA: ACM; 1991;23:5–48.

102. Shirts M, Pande VS. COMPUTING: Screen Savers of the World Unite! Science. 2000;290:1903–4.

103. Bird I. Computing for the Large Hadron Collider. Annu. Rev. Nucl. Part. Sci. 2011;61:99–118.

104. Anderson DP. BOINC: A System for Public Resource Computing and Storage. Proceedings of the Fifth IEEE/ACM International Workshop on Grid Computing (GRID'04). 2004.

105. Ransohoff DF. Bias as a threat to the validity of cancer molecular-marker research. Nat. Rev. Cancer. 2005;5:142–9.

106. Bild AH, Chang JT, Johnson WE, Piccolo SR. A field guide to genomics research. PLoS Biol. 2014;12:e1001744.

107. Ratcliffe S. Concise Oxford Dictionary of Quotations. Oxford University Press; 2011. p. 389.

Figure 1 Click here to download Figure Figure_1.jpg

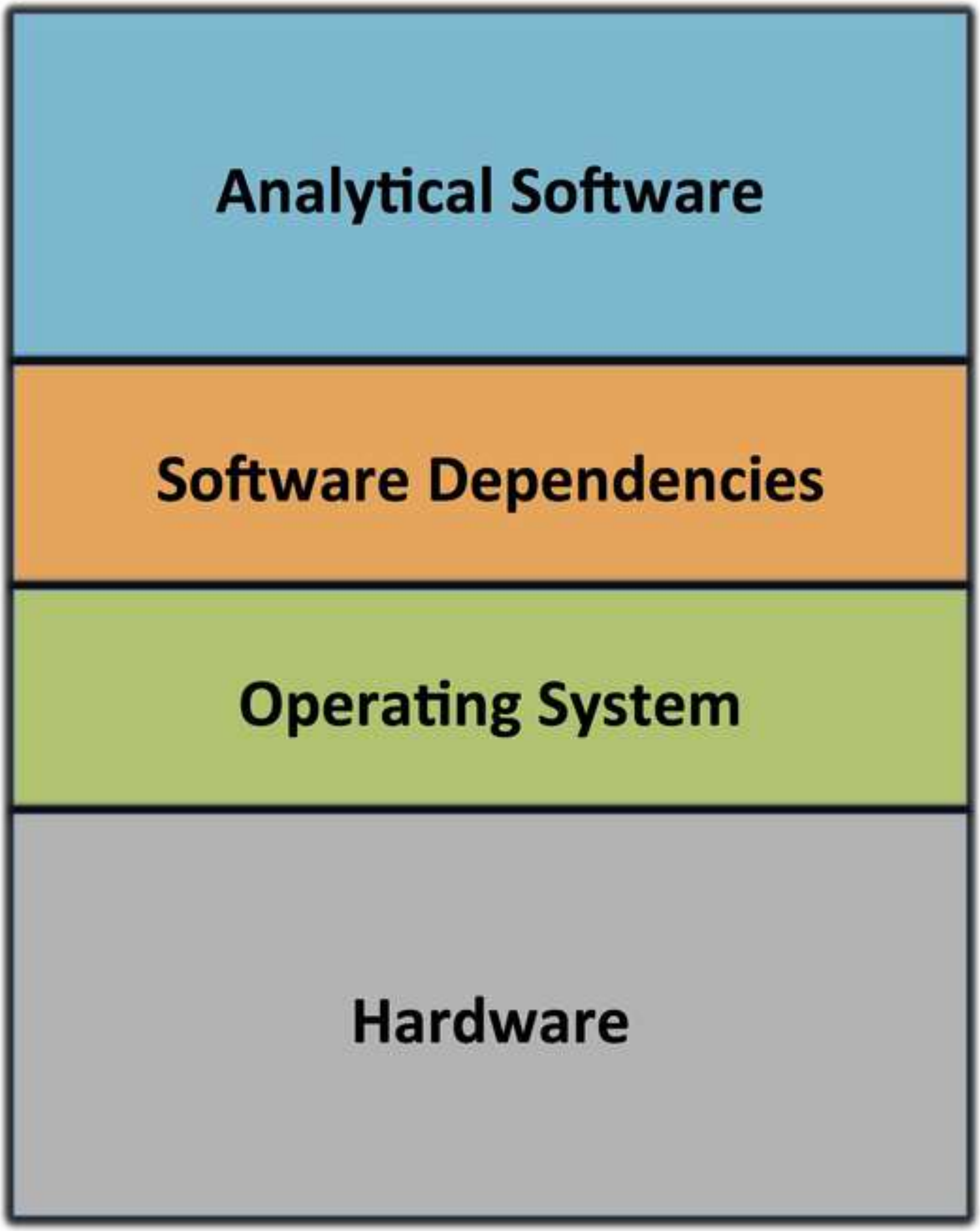

Figure 2

Click here to download Figure Figure_2.jpg

```
#!/bin/bash

# Download software, reference genome, and FASTQ files
wget http://downloads.sourceforge.net/project/bio-bwa/bwakit/bwakit-0.7.12_x64-linux.tar.bz2
wget -O refGenomeFiles.zip https://ndownloader.figshare.com/files/4841809
wget -O FASTQ.zip https://ndownloader.figshare.com/articles/3114454/versions/1

# Extract software and reference genome files
tar -jxvf bwakit-0.7.12_x64-linux.tar.bz2
unzip refGenomeFiles.zip
unzip FASTQ.zip

# Align FASTQ data to reference genome and save to SAM file
bwa.kit/bwa mem  KJ660346.fa SRR1972917_1.fastq SRR1972917_1.fastq > SRR1972917_aligned.sam

# Convert SAM file to BAM file
bwa.kit/samtools view -bS SRR1972917_aligned.sam > SRR1972917_aligned.bam

# Sort BAM file
bwa.kit/samtools sort SRR1972917_aligned.bam SRR1972917_aligned_sorted

# Index BAM file
bwa.kit/samtools index SRR1972917_aligned_sorted.bam
```

Figure 3

Click here to download Figure Figure_3.jpg

```
all: download prepare align buildbam

buildbam: SRR1972917_aligned.sam
	# Convert SAM file to BAM file
	bwa.kit/samtools view -bS SRR1972917_aligned.sam > SRR1972917_aligned.bam

	# Sort BAM file
	bwa.kit/samtools sort SRR1972917_aligned.bam SRR1972917_aligned_sorted

	# Index BAM file
	bwa.kit/samtools index SRR1972917_aligned_sorted.bam

align: bwa.kit/bwa SRR1972917_1.fastq SRR1972917_2.fastq KJ660346.fa
	# Align FASTQ data to reference genome and save to SAM file
	bwa.kit/bwa mem  KJ660346.fa SRR1972917_1.fastq SRR1972917_1.fastq > SRR1972917_aligned.sam

download:
	# Download software, reference genome, and FASTQ files
	wget http://downloads.sourceforge.net/project/bio-bwa/bwakit/bwakit-0.7.12_x64-linux.tar.bz2
	wget -O refGenomeFiles.zip https://ndownloader.figshare.com/files/4841809
	wget -O FASTQ.zip https://ndownloader.figshare.com/articles/3114454/versions/1

prepare: refGenomeFiles.zip FASTQ.zip bwakit-0.7.12_x64-linux.tar.bz2
	# Extract software and reference genome files
	tar -jxvf bwakit-0.7.12_x64-linux.tar.bz2
	unzip refGenomeFiles.zip
	unzip FASTQ.zip
```

Figure 4 Click here to download Figure Figure_4.jpg

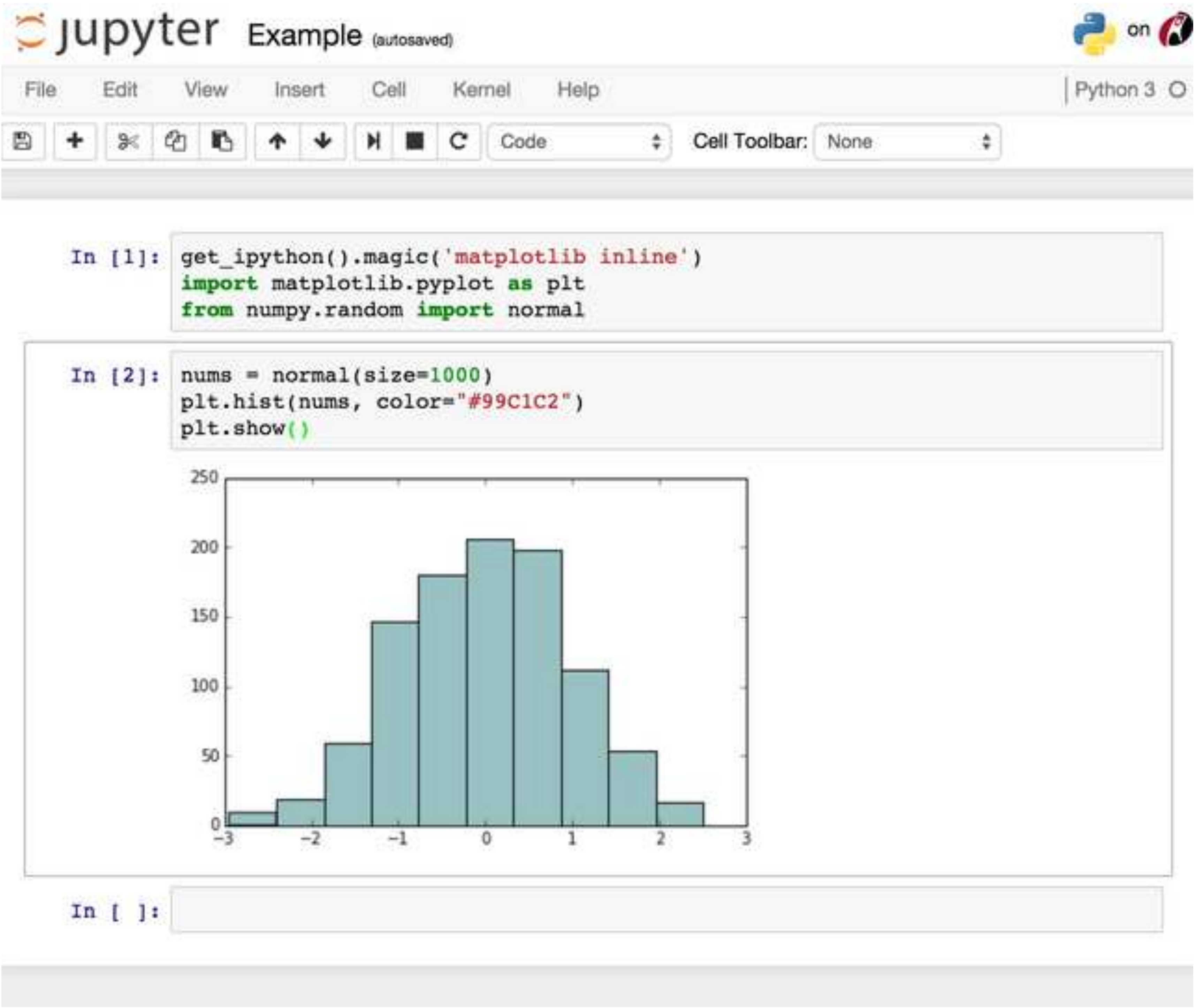

R Markdown Example

1. Set the random seed.

```
set.seed(99)
```

2. Generate 1000 random normal numbers

```
norm <- rnorm(1000)
```

3. Plot them as a histogram

```
hist(norm, col="#99C1C2")
```

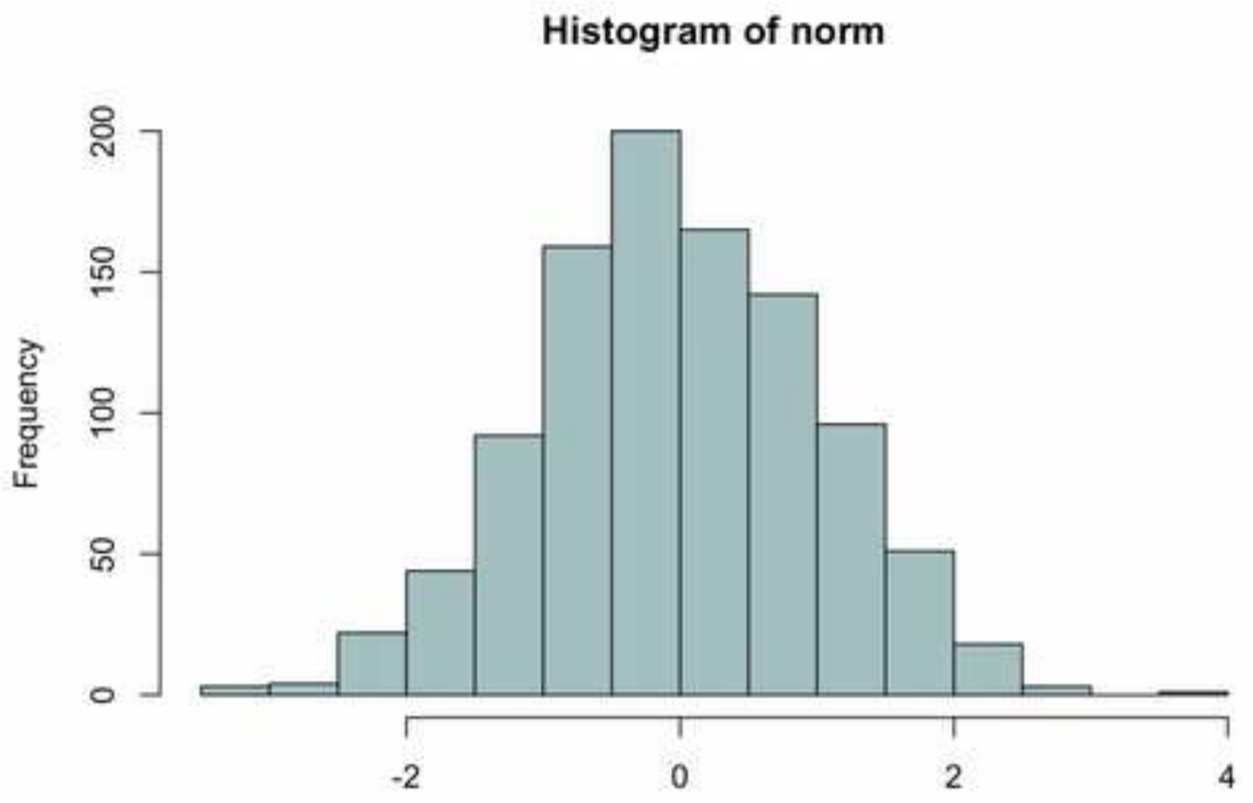

Figure 6

Click here to download Figure Figure_6.jpg

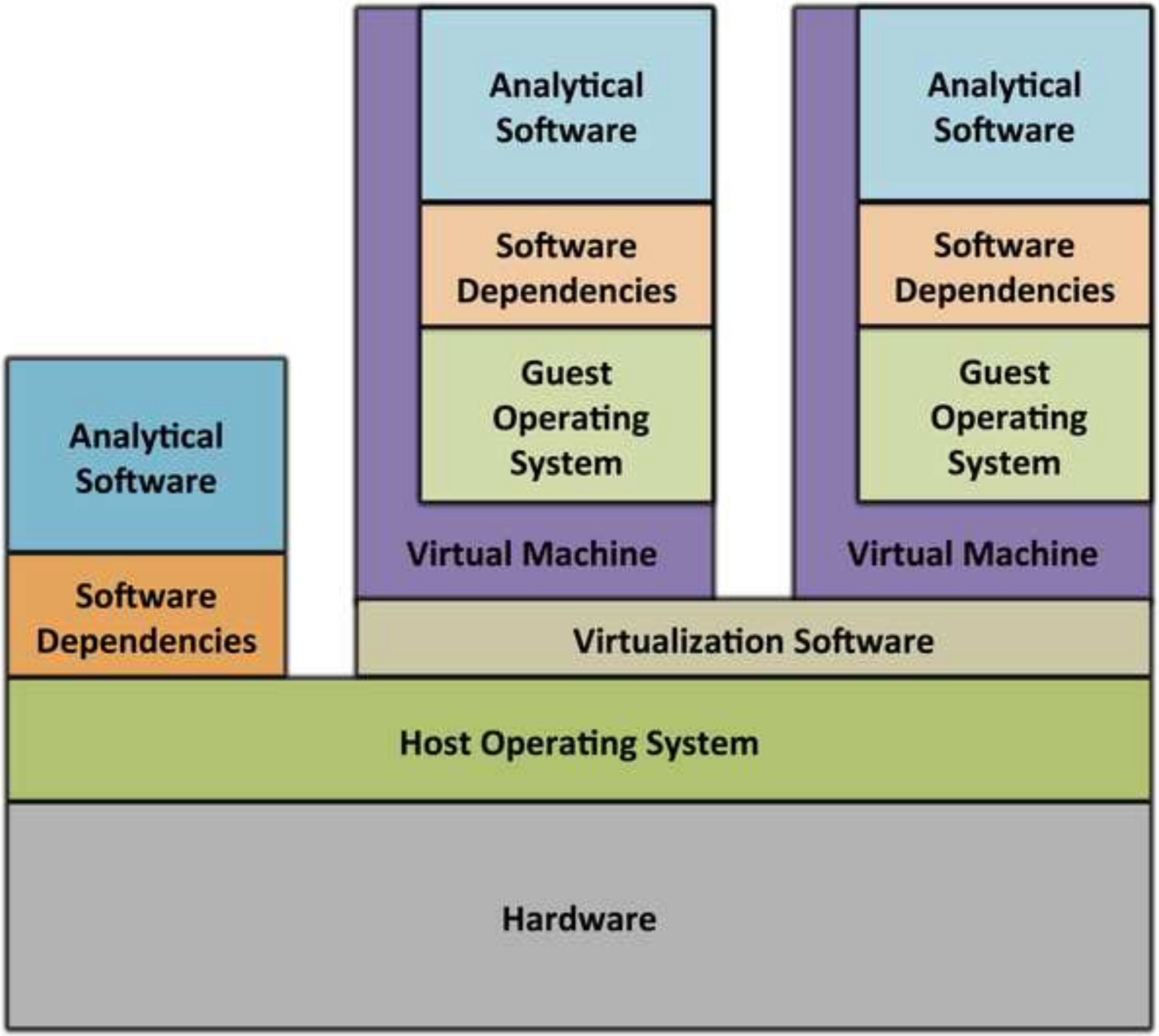

Figure 7

Click here to download Figure Figure_7.jpg

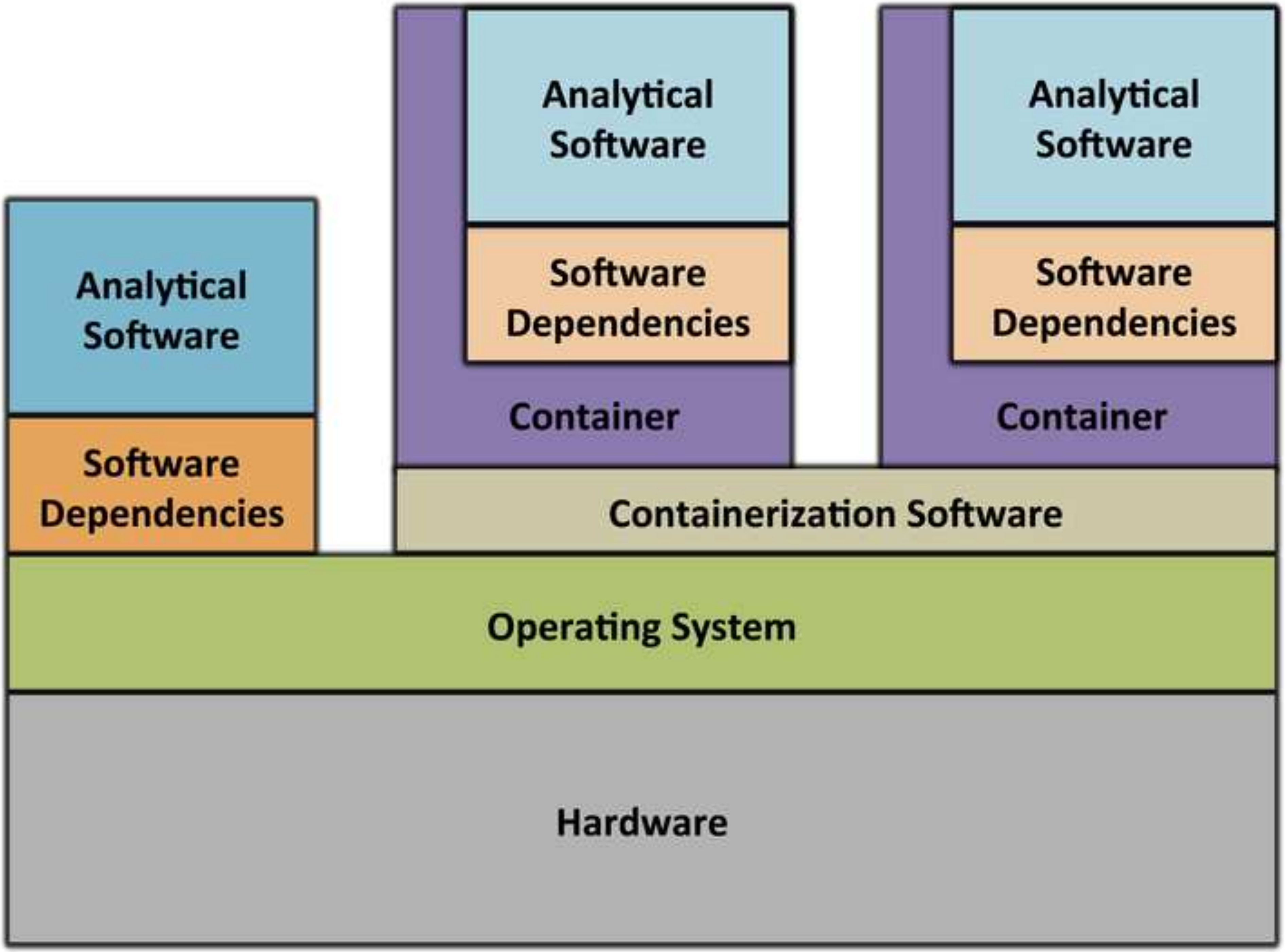

Figure 8

Click here to download Figure Figure_8.jpg

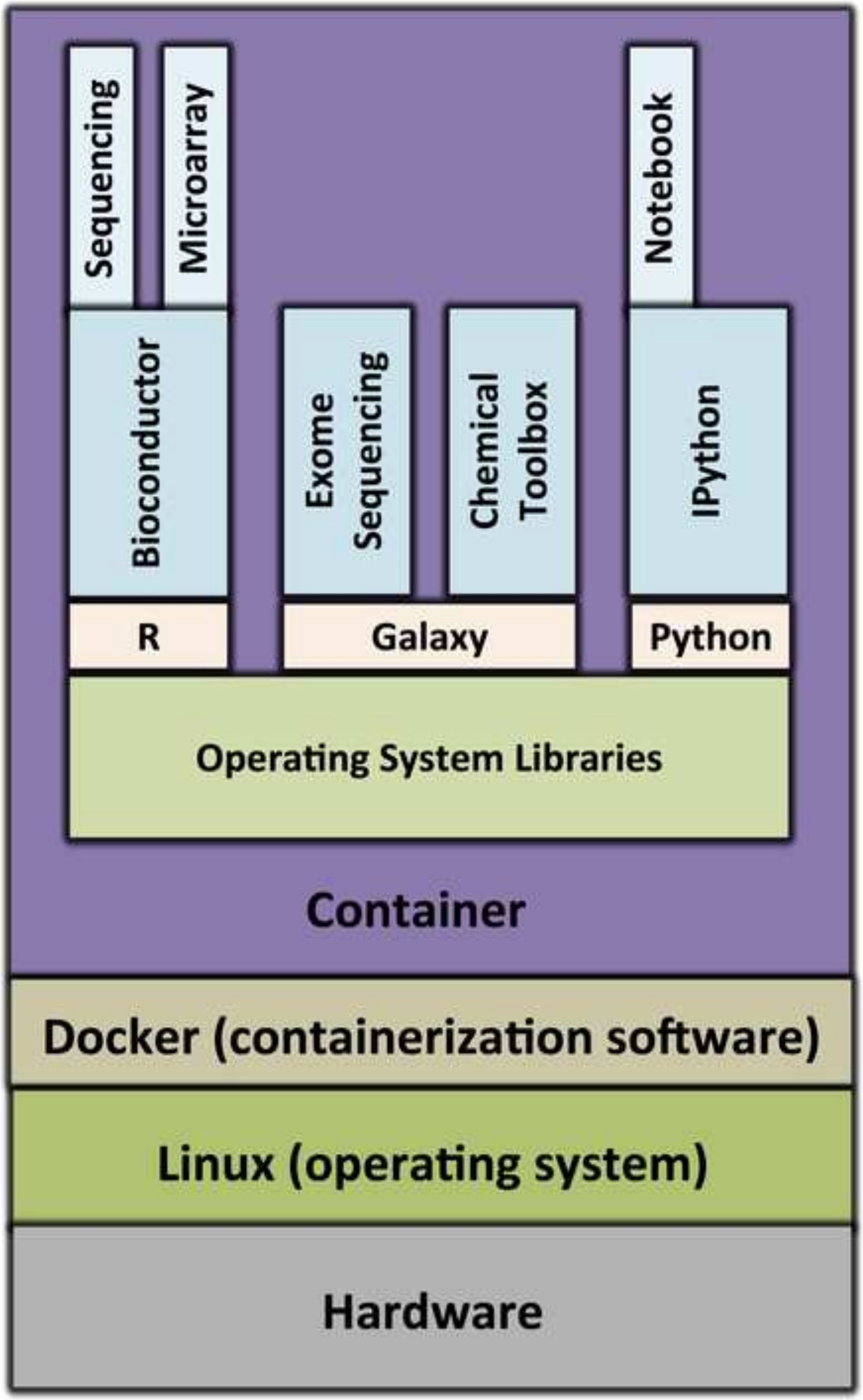

GigaScience

Tools and techniques for computational reproducibility

--Manuscript Draft--

**Manuscript Number:**	GIGA-D-16-00020R1
**Full Title:**	Tools and techniques for computational reproducibility
**Article Type:**	Review
**Funding Information:**	
**Abstract:**	When reporting research findings, scientists document the steps they followed so that others can verify and build upon the research. When those steps have been described in sufficient detail that others can retrace the steps and obtain similar results, the research is said to be reproducible. Computers play a vital role in many research disciplines and present both opportunities and challenges for reproducibility. Computers can be programmed to execute analysis tasks, and those programs can be repeated and shared with others. The deterministic nature of most computer programs means that the same analysis tasks, applied to the same data, will often produce the same outputs. However, in practice, computational findings often cannot be reproduced because of complexities in how software is packaged, installed, and executed—and because of limitations associated with how scientists document analysis steps. Many tools and techniques are available to help overcome these challenges; here we describe seven such strategies. With a broad scientific audience in mind, we describe the strengths and limitations of each approach, as well as the circumstances under which each might be applied. No single strategy is sufficient for every scenario; thus we emphasize that it is often useful to combine approaches.
**Corresponding Author:**	Stephen R Piccolo, Ph.D.<br>Brigham Young University<br>Provo, UT UNITED STATES
**Corresponding Author Secondary Information:**	
**Corresponding Author's Institution:**	Brigham Young University
**Corresponding Author's Secondary Institution:**	
**First Author:**	Stephen R Piccolo, Ph.D.
**First Author Secondary Information:**	
**Order of Authors:**	Stephen R Piccolo, Ph.D.
	Michael B Frampton
**Order of Authors Secondary Information:**	
**Response to Reviewers:**	Dear Dr. Edmunds:<br><br>We thank you and the reviewers for providing helpful comments on our review article, “Tools and techniques for computational reproducibility” (GIGA-D-16-00020). We have edited the manuscript according to the reviewers’ suggestions. We have also added a reference to several papers that have used a Docker container for their analysis, and to Eglen, et al., which used knitr. These changes have improved our manuscript.<br><br>Below is a point-by-point response to the reviewers’ comments.<br><br>Thank you for considering our article. Please let me know if we can answer any questions you may have.<br><br>Warm regards,<br><br>Stephen R. Piccolo, PhD<br>Assistant Professor

Powered by Editorial Manager® and ProduXion Manager® from Aries Systems Corporation

Department of Biology
Brigham Young University
(801) 422-7116
Stephen_Piccolo@byu.edu

** In the point-by-point replies below, the reviewer's comments are shown first. Our responses are prefixed by >> characters. **

Reviewer #1 (Titus Brown)

In this paper, Piccolo et al. do a nice (and I think comprehensive?) job of outlining strategies for computational reproducibility. The point is well made that science is increasingly dependent on computational reproducibility (and that in theory we should be able to do computational reproducibility easily and well) and hence we should explore effective approaches that are actually being used.

I know of no other paper that covers this array of material, and this is a quite nice exposition that I would recommend to many. I can't evaluate how broadly it will appeal to a diverse audience but it seems very readable to me.

>> We thank the reviewer for taking time to review our manuscript and for these positive comments!

This paper is a nice complement to Ten Simple Rules...,
http://journals.plos.org/ploscompbiol/article?id=10.1371/journal.pcbi.1003285, in that it is longer, more in depth, and provides more explicit recommendations. It is also more up to date.

>> Thanks for this comment. Our goal, in part, was to extend beyond what the Ten Simple Rules… article covers. We cite that article multiple times within our manuscript.

I reviewed this paper previously, and it has been updated in light of my (and other) reviewers' comments. I was positive about it then, and the revisions are good ;). One note is that the 'binder' service (http://mybinder.org) should be mentioned as a harbinger of the future. I have a blog post summary of it here: ivory.idyll.org/blog/2016-mybinder.html

Signed,
C. Titus Brown
ctbrown@ucdavis.edu

>> Thanks for this suggestion. We have updated the second sentence of the Discussion section. We mention Binder (and Everware) and describe them as potential "harbingers of the future." (I hope it's OK that we borrowed this phrase from Titus.)

Reviewer #2 (Stephen Eglen)

Summary

This review provides an overview of the main tools and techniques used to ensure computational reproducibility of results. This review is a good fit to the readership of the Gigascience journal.

>> We thank the reviewer for taking time to review this manuscript and for these positive comments!

Overall I found the manuscript to be clearly written and would recommend it for publication, although I would like the authors to consider the following issues in a potential revision. In particular, I am most critical about the current set of figures. As a general comment, where possible, the material in the figures should be supported by

Powered by Editorial Manager® and ProduXion Manager® from Aries Systems Corporation

online files (e.g. knitr, Docker) so that the reader can immediately get workable examples to examine, run and amend.

>> We have addressed these comments below. We now provide executable files to complement Figures 1-4. We have included these in the supplementary material. We have also improved these materials based on the reviewer's comments.

Detailed comments

p4, l2 (page 4, line 2): an extra citation might be (Gronenschild et al. 2012) which showed that even the choice of operating system or neuroimaging software version affects results.

>> We have added this citation at the suggested location.

p4, l18-20: as far as I am aware, journals do not encourage direct use of repositories like github or bitbucket because they offer no long-term storage. It is regarded much more appropriate to use permanent URLS (e.g. DOIs) to point to archived versions of software, such as zenodo. We have written elsewhere on this topic (Eglen et al. 2016).

>> We have updated this text so that it no longer mentions GitHub and Bitbucket as examples of storage locations for archived versions of code. Now we mention Zenodo and figshare as services that provide permanent DOIs.

p6, l10: in addition to the other topics of reproducibility and education, it would be worth mentioning education/training to encourage users to adopt reproducible practices (Wilson 2016).

>> We have added a reference to Wilson 2016 and have mentioned "education about reproducibility" as a topic that is covered elsewhere.

p6, l15: minor point, but perhaps worth numbering the seven sections that describe the seven
approaches reviewed, starting here.

>> We have numbered these sections as an aid to the reader.

p8, l17: "Make can be configured"; if you are referring to the "-j N" switch, then it is simpler to say that "Make can automatically identify. . . " as there is no extra configuration needed.

>> We have made this change to the text. Thanks for pointing it out.

p11 l9: reference 53 at the end of the sentence is not needed, as you refer to it at the start of the
sentence.

>> We have removed this citation at the end of the sentence.

p13, l3-9: you should probably mention http://mybinder.org in this section. It provides a transparent method for interacting with Jupyter documents over the web (Rosenberg and Horn 2016).

>> Thanks for this suggestion. We have updated the second sentence of the Discussion section. We mention Binder (and Everware, a similar service) and cite the paper by Rosenberg and Horn.

p14, l2: I disagree slightly here with the view re: long-running jobs. knitr at least can cache
intermediate computations transparently which helps enormously.

>> We have modified this paragraph to indicate that long-running notebooks can be executed at the command line.

Powered by Editorial Manager® and ProduXion Manager® from Aries Systems Corporation

	p14, l8: Do you have examples of Dexy in use within this field? Asking this also in a more general way, it might be worth making a table listing examples explicitly of each of the seven approaches, so that they are easy to find.<br><br>>> We haven't found any substantive examples where Dexy is used in a biology-focused study. We have included it more as an indicator that alternative literate programming environments may be useful in cases where Juptyer and knitr are not suitable.<br><br>>> Although it may be useful to add a table that lists examples where each of the seven methods is used, we have cited various such studies throughout the text. We hope these references will provide useful examples, but we wish to avoid putting too much emphasis on any individual study.<br><br>p18, l18: I do not see why the VMs are "black boxes". Surely to create the VM all the relevant<br>code must be provided, so that you can at least examine what is done, or extend the analysis (as mentioned on l20). Can you clarify what you mean here.<br><br>>> We have rewritten this paragraph to clarify our points. Our goal was to emphasize the importance of providing easy access to scripts and code that are used within a virtual machine for an analysis. Although it would be possible for other scientists to examine the full contents of a virtual machine, it is less convenient (and perhaps less transparent). So we encourage the idea of storing these scripts and code in a public repository, separate from the virtual machine. C. Titus Brown (reviewer #1) has also emphasized this point on his blog, which we cite: http://ivory.idyll.org/blog/vms-considered-harmful.html.<br><br>p22, l1: as well as capturing software in Docker, we have used it recently to capture the entire<br>environment to write our research papers in knitr<br>(https://hub.docker.com/r/sje30/eglen2015/<br>and https://hub.docker.com/r/sje30/waverepo/).<br><br>>> Thanks for pointing to these. We now cite these papers as examples of enabling others to execute analyses described in research papers.<br><br>p22, l14-19: I found the section about other operating systems (Windows/mac) rather clumsy.<br>From the user's perspective, the modern docker toolbox seems to work smoothly enough (at least on macs) that the details at the end of p22 seem irrelevant. It might instead be worth mentioning that docker builds can be automatically triggered, e.g. upon new commits to github.<br><br>>> Thanks for this suggestion. Indeed, the Docker toolbox has been updated recently so that it is much more convenient to install and execute Docker on non-Linux machines. We have simplified the text accordingly. We have also clarified that the overhead of using a virtual machine to execute Docker containers on Windows or Mac operating systems offsets some of the performance benefits of containers.<br><br>p 23, l6: "Scientific advancement requires trust." I think trust is the wrong word here. e.g. The Royal Society's motto 'Nullius in verba' is taken to mean 'take nobody's word for it'<br>(https://royalsociety.org/about-us/history/). Rather, what these tools do is promote transparency<br>to reduce the barriers for others to repeat prior work. With this in mind, I'd suggest the authors<br>re-read this first paragraph of the discussion to see whether they think "trust" is what they are<br>promoting here.<br><br>>> Thanks for this insight. We have revised the first paragraph of the Discussion section. It no longer uses the word "trust" but instead focuses on the ideas of explicitly documenting research steps and being transparent about the way those steps are

Powered by Editorial Manager® and ProduXion Manager® from Aries Systems Corporation

shared.

Figures

Figure 1: is this really needed in such a review? I don't think it adds anything.

>> After considering this feedback, we agree that this figure is not needed, and we have removed it from the manuscript.

Figure 2: I think should be provided as a text file so that people can run it for themselves.

>> We now provide a text version of this script as a supplementary file.

Figure 3: Text file definitely needed so people can run it for themselves. But also I'd consider
making the targets a bit more specific. All of the targets in this example Makefile are PHONY and perhaps it could be rewritten in the more canonical Makefile style? The way it is currently written, it is hard to see how this differs from a shell script. (Each time make all is run, won't the files be downloaded again?)

>> Thanks for pointing this out. We have modified the example Makefile so that it follows a more conventional style. Now it will only download the files (and execute the other steps) one time (if the steps executed successfully). In addition, we now provide a text version as a supplementary file.

Figure 4: can you show something that is a bit more bio-relevant, e.g. some genomic analysis?

>> We have updated this figure so that it shows a plot of simulated gene-expression data.

Figure 5: as figure 4. Can you design Figure 4 and 5 carefully to highlight the differences between knitr and jupyter?

>> We have updated this figure so it also shows a plot simulated-gene expression data. We are unsure which differences between knitr and Jupyter that the reviewer would like us to point out. However, we believe the updated figures show a more distinct difference between the two.

Figure 6-8: I would suggest you drop these. They show the notion of stacks and containers, but
most bioscientists won't get much value from them.

>> We respect the reviewer's point of view; however, given feedback from scientists who have read our preprint, we feel that some scientists will benefit from these figures. Thus we prefer to retain them.

References

Eglen, Stephen, Ben Marwick, Yaroslav Halchenko, Michael Hanke, Shoaib Sufi, Padraig Gleeson, R Angus Silver, et al. 2016. "Towards Standard Practices for Sharing Computer Code and Programs in Neuroscience." BioRxiv. doi:10.1101/045104.

Gronenschild, Ed H B M, Petra Habets, Heidi I L Jacobs, Ron Mengelers, Nico Rozendaal, Jim van Os, and Machteld Marcelis. 2012. "The Effects of FreeSurfer Version, Workstation Type, and Macintosh Operating System Version on Anatomical Volume and Cortical Thickness Measurements." PLoS One 7 (6): e38234. doi:10.1371/journal.pone.0038234.

Rosenberg, David M, and Charles C Horn. 2016. "Neurophysiological Analytics for All! Free OpenSource Software Tools for Documenting, Analyzing, Visualizing, and Sharing Using Electronic Notebooks." J. Neurophysiol., 20~apr, jn.00137.2016. doi:10.1152/jn.00137.2016.

	Wilson, Greg. 2016. "Software Carpentry: Lessons Learned." F1000Res. 3 (28~jan). doi:10.12688/f1000research.3-62.v2.
**Additional Information:**	
**Question**	**Response**
**Experimental design and statistics**<br><br>Full details of the experimental design and statistical methods used should be given in the Methods section, as detailed in our Minimum Standards Reporting Checklist. Information essential to interpreting the data presented should be made available in the figure legends.<br><br>Have you included all the information requested in your manuscript?	No
If not, please give reasons for any omissions below.<br><br>as follow-up to "**Experimental design and statistics**<br><br>Full details of the experimental design and statistical methods used should be given in the Methods section, as detailed in our Minimum Standards Reporting Checklist. Information essential to interpreting the data presented should be made available in the figure legends.<br><br>Have you included all the information requested in your manuscript?<br><br>"	It is a review article.
**Resources**<br><br>A description of all resources used, including antibodies, cell lines, animals and software tools, with enough information to allow them to be uniquely identified, should be included in the Methods section. Authors are strongly encouraged to cite Research Resource Identifiers (RRIDs) for antibodies, model organisms and tools, where possible.<br><br>Have you included the information requested as detailed in our Minimum Standards Reporting Checklist?	No

Powered by Editorial Manager® and ProduXion Manager® from Aries Systems Corporation

If not, please give reasons for any omissions below.<br><br>as follow-up to "**Resources**<br><br>A description of all resources used, including antibodies, cell lines, animals and software tools, with enough information to allow them to be uniquely identified, should be included in the Methods section. Authors are strongly encouraged to cite Research Resource Identifiers (RRIDs) for antibodies, model organisms and tools, where possible.<br><br>Have you included the information requested as detailed in our Minimum Standards Reporting Checklist?<br><br>"	It is a review article.
**Availability of data and materials**<br><br>All datasets and code on which the conclusions of the paper rely must be either included in your submission or deposited in publicly available repositories (where available and ethically appropriate), referencing such data using a unique identifier in the references and in the "Availability of Data and Materials" section of your manuscript.<br><br>Have you have met the above requirement as detailed in our Minimum Standards Reporting Checklist?	No
If not, please give reasons for any omissions below.<br><br>as follow-up to "**Availability of data and materials**<br><br>All datasets and code on which the conclusions of the paper rely must be either included in your submission or deposited in publicly available repositories (where available and ethically appropriate), referencing such data using a unique identifier in the references and in the "Availability of Data and Materials" section of your manuscript.<br><br>Have you have met the above requirement as detailed in our Minimum	It is a review article.

Powered by Editorial Manager® and ProduXion Manager® from Aries Systems Corporation

Standards Reporting Checklist?<br><br>"	

Powered by Editorial Manager® and ProduXion Manager® from Aries Systems Corporation

Revised Manuscript
Click here to view linked References
Click here to download Manuscript Piccolo_Manuscript_Gigascience_v2_edit_sp.docx

Tools and techniques for computational reproducibility

Stephen R. Piccolo[1*], Michael B. Frampton[2]

[1]Department of Biology, Brigham Young University, Provo, UT, USA

[2]Department of Computer Science, Brigham Young University, Provo, UT, USA

* Corresponding author

4102 LSB

Brigham Young University

Provo

UT 84602

USA

Telephone: +1 801-422-7116

Email: stephen_piccolo@byu.edu

Twitter: @stevepiccolo

ORCID: 0000-0003-2001-5640

Abstract

When reporting research findings, scientists document the steps they followed so that others can verify and build upon the research. When those steps have been described in sufficient detail that others can retrace the steps and obtain similar results, the research is said to be reproducible. Computers play a vital role in many research disciplines and present both opportunities and challenges for reproducibility. Computers can be programmed to execute analysis tasks, and those programs can be repeated and shared with others. The deterministic nature of most computer programs means that the same analysis tasks, applied to the same data, will often produce the same outputs. However, in practice, computational findings often cannot be reproduced because of complexities in how software is packaged, installed, and executed—and because of limitations associated with how scientists document analysis steps. Many tools and techniques are available to help overcome these challenges; here we describe seven such strategies. With a broad scientific audience in mind, we describe the strengths and limitations of each approach, as well as the circumstances under which each might be applied. No single strategy is sufficient for every scenario; thus we emphasize that it is often useful to combine approaches.

Keywords: Computational reproducibility; Practice of science; Literate programming; Virtualization; Software containers; Software frameworks

Introduction

When reporting research, scientists document the steps they followed to obtain their results. If the description is comprehensive enough that they and others can repeat the procedures and obtain semantically consistent results, the findings are considered to be "reproducible" [1–6]. Reproducible research forms the basic building blocks of science, insofar as it allows researchers to verify and build on each other's work with confidence.

Computers play an increasingly important role in many scientific disciplines [7–10]. For example, in the United Kingdom, 92% of academic scientists use some type of software in their research, and 69% of scientists say their research is feasible only with software tools [11]. Thus efforts to increase scientific reproducibility should consider the ubiquity of computers in research.

Computers present both opportunities and challenges for scientific reproducibility. On one hand, the deterministic nature of most computer programs means that identical results can be obtained from many computational analyses applied to the same input data [12]. Accordingly, computational research can be held to a high reproducibility standard. On the other hand, even when no technical barrier prevents reproducibility, scientists often cannot reproduce computational findings because of complexities in how software is packaged, installed, and executed—and because of limitations associated with how scientists document these steps [13]. This problem is acute in many disciplines, including genomics, signal processing, and ecological modeling [14–16], which have large data sets and rapidly evolving computational tools. However, the same problem can affect any

scientific discipline requiring computers for research. Seemingly minor differences in computational approaches can have major influences on analytical outputs [12, 17–22], and the effects of these differences may exceed those resulting from experimental factors [23].

Journal editors, funding agencies, governmental institutions, and individual scientists have increasingly made calls for the scientific community to embrace practices to support computational reproducibility [24–31]. This movement has been motivated, in part, by scientists' failed efforts to reproduce previously published analyses. For example, Ioannidis et al. evaluated 18 published research studies that used computational methods to evaluate gene expression data, but they were able to reproduce only two of those studies [32]. In many cases, the culprit was a failure to share the study's data; however, incomplete descriptions of software-based analyses were also common. Nekrutenko and Taylor examined 50 papers that analyzed next-generation sequencing data and observed that fewer than half provided any details about software versions or parameters [33]. Recreating analyses that lack such details can require hundreds of hours of effort [34] and may be impossible, even after consulting the original authors. Failure to reproduce research may also lead to careerist effects, including retractions [35].

Noting such concerns, some journals have emphasized the value of placing computer code in open access repositories. It is most useful when scientists provide direct access to an archived version of the code via a uniform resource locator (URL). For example, Zenodo.org and figshare.com provide permanent digital object identifiers (DOI) that can link to

software code (and other digital objects) used in publications. In addition, some journals have extended requirements for “Methods” sections, now asking researchers to provide detailed descriptions of 1) how to install software and its dependencies, and 2) what parameters and data preprocessing steps are used in analyses [10, 24]. A 2012 Institute of Medicine report emphasized that, in addition to computer code and research data, “fully specified computational procedures” should be made available to the scientific community [25]. The report’s authors elaborated that such procedures should include “all of the steps of computational analysis”, and that “all aspects of the analysis need to be transparently reported” [25]. Such policies represent important progress. However, it is ultimately the responsibility of individual scientists to ensure that others can verify and build upon their analyses.

Describing a computational analysis sufficiently—such that others can re-execute, validate, and refine it—requires more than simply stating what software was used, what commands were executed, and where to find the source code [13, 27, 36–38]. Software is executed within the context of an operating system (for example, Windows, Mac OS, or Linux), which enables the software to interface with computer hardware. In addition, most software relies on a hierarchy of software dependencies, which perform complementary functions and must be installed alongside the main software tool. One version of a given software tool or dependency may behave differently or have a different interface than another version of the same software. In addition, most analytical software offers a range of parameters (or settings) that the user can specify. If any of these variables differs from those used by the original experimenter, the software may not execute properly or

analytical outputs may differ considerably from those observed by the original experimenter.

Scientists can use various tools and techniques to overcome these challenges and to increase the likelihood that their computational analyses will be reproducible. These techniques range in complexity from simple (e.g., providing written documentation) to advanced (e.g., providing a virtual environment that includes an operating system and all the software necessary to execute the analysis). This review describes seven strategies across this spectrum. We describe many of the strengths and limitations of each approach, as well as the circumstances under which each might be applied. No single strategy will be sufficient for every scenario; therefore, in many cases, it will be most practical to combine multiple approaches. This review focuses primarily on the computational aspects of reproducibility. The related topics of empirical reproducibility, statistical reproducibility, data sharing, and education about reproducibility have been described elsewhere [39–46]. We believe that with greater awareness and understanding of computational reproducibility techniques, scientists—including those with limited computational experience—will be more apt to perform computational research in a reproducible manner.

1. Narrative descriptions are a simple but valuable way to support computational reproducibility

The most fundamental strategy for enabling others to reproduce a computational analysis is to provide a detailed, written description of the process. For example, when reporting computational results in a research article, authors customarily provide a narrative that describes the software they used and the analytical steps they followed. Such narratives can be invaluable in enabling others to evaluate the scientific approach and to reproduce the findings. In many situations—for example, when software execution requires user interaction or when proprietary software is used—narratives are the only feasible option for documenting such steps. However, even when a computational analysis uses open-source software and can be fully automated, narratives help others understand how to re-execute an analysis.

Although most articles about research that uses computational methods provide some type of narrative, these descriptions often lack sufficient detail to enable others to retrace those steps [32, 33]. Narrative descriptions should indicate the operating system(s), software dependencies, and analytical software that were used, and how to obtain them. In addition, narratives should indicate the exact software versions used, the order in which they were executed, and all non-default parameters that were specified. Such descriptions should account for the fact that computer configurations can differ vastly, even for computers with the same operating system. Because it can be difficult for scientists to remember such

details after the fact, it is best to record this information throughout the research process, rather than at the time of manuscript preparation [8].

The following sections describe techniques for automating computational analyses. These techniques can diminish the need for scientists to write narratives. However, because it is often impractical to automate all computational steps, we expect that, for the foreseeable future, narratives will play a vital role in enabling computational reproducibility.

2. Custom scripts and code can automate research analysis

Scientific software can often be executed in an automated manner via text-based commands. Using such commands—via a command-line interface—scientists can indicate the software program(s) to be executed and which parameter(s) should be used. When multiple commands must be executed, they can be compiled into scripts specifying the order in which the commands should be executed (Figure 1; Additional file 1). In many cases, scripts also include commands for installing and configuring software. Such scripts serve as valuable documentation not only for individuals who wish to re-execute the analysis, but also for the researcher who performed the original analysis [47]. In these cases, no amount of narrative is an adequate substitute for providing the actual commands that were used.

When writing command-line scripts, it is essential to explicitly document any software dependencies and input data that are required for each step in the analysis. The Make utility [114] provides one way to specify such requirements [36]. Before any command is

executed, Make verifies that each documented dependency is available. Accordingly, researchers can use Make files (scripts) to specify a full hierarchy of operating system components and dependent software that must be present to perform the analysis (Figure 2; Additional file 2). In addition, Make can automatically identify any commands that can be executed in parallel, potentially reducing the amount of time required for the analysis. Although Make was originally designed for UNIX-based operating systems (such as Mac OS or Linux), similar utilities have since been developed for Windows operating systems [116]. Box 1 lists various utilities that can be used to automate software execution.

Box 1. Utilities that can be used to automate software execution

- GNU Make and Make for Windows: tools for building software from source files and for ensuring that the software's dependencies are met.
- Snakemake [48]: an extension of Make that provides a more flexible syntax and makes it easier to execute tasks in parallel.
- BPipe [49]: a tool that provides a flexible syntax for users to specify commands to be executed; it maintains an audit trail of all commands that have been executed.
- GNU Parallel [50]: a tool for executing commands in parallel across one or more computers.
- Makeflow [51]: a tool that can execute commands simultaneously on various

types of computer architectures, including computer clusters and cloud environments.

- SCONS [52]: an alternative to GNU Make that enables users to customize the process of building and executing software using scripts written in the Python programming language.
- CMAKE.org: a tool that enables users to execute Make scripts more easily on multiple operating systems.

As well as creating scripts to execute existing software, many researchers also create new software by writing computer code in a programming language such as Python, C++, Java, or R. Such code may perform relatively simple tasks, such as reformatting data files or invoking third-party software. In other cases, computer code may constitute a manuscript's key intellectual contribution.

Whether analysis steps are encoded in scripts or as computer code, scientists can support reproducibility by publishing these artifacts alongside research papers. By doing so, authors enable readers to evaluate the analytical approach in full detail and to extend the analysis more readily [53]. Although scripts and code may be included alongside a manuscript as supplementary material, a better alternative is to store them in a public repository with a permanent URL. It is often also useful to store code in a version control system (VCS) [8, 9, 47], and to share it via Web-based services like GitHub.com or Bitbucket.org [54]. With such a VCS repository, scientists can track the different versions of

scripts and code that have been developed throughout the evolution of the research project. In addition, outside observers can see the full version history, contribute revisions to the code, and reuse the code for their own purposes [55]. When submitting a manuscript, the authors may "tag" a specific version of the repository that was used for the final analysis described in the manuscript.

3. Software frameworks enable easier handling of software dependencies

Virtually all computer scripts and code relies on external software dependencies and operating system components. For example, suppose a research study required a scientist to apply Student's t-test. Rather than write code to implement this statistical test, the scientist would likely find an existing software library that implements the test and then invoke that library from their code. Much time can be saved with this approach, and a wide range of software libraries are freely available. However, software libraries change frequently; invoking the wrong version of a library may result in an error or an unexpected output. Thus, to enable others to reproduce an analysis, it is critical to indicate which dependencies (and versions thereof) must be installed.

One way to address this challenge is to build on a pre-existing software framework, which makes it easier to access software libraries that are commonly used to perform specific types of analysis task. Typically, such frameworks also make it easier to download and install software dependencies, and to ensure that the versions of software libraries and

their dependencies are compatible with each other. For example, Bioconductor [56], created for the R statistical programming language [57], is a popular framework that contains hundreds of software packages for analyzing biological data. The Bioconductor framework facilitates versioning, documenting, and distributing code. Once a software library has been incorporated into Bioconductor, other researchers can find, download, install, and configure it on most operating systems with relative ease. In addition, Bioconductor installs software dependencies automatically. These features ease the process of performing an analysis, and can help with reproducibility. Various software frameworks exist for other scientific disciplines [58–63]. General purpose tools for managing software dependencies also exist, for example, Apache Ivy [116] and Puppet [117].

To best support reproducibility, software frameworks should make it easy for scientists to download and install previous versions of a software tool, as well as previous versions of dependencies. Such a design enables other scientists to reproduce analyses that were conducted with previous versions of a software framework. In the case of Bioconductor, considerable extra work may be required to install specific versions of Bioconductor software and their dependencies. To overcome these limitations, scientists may use a software container or virtual machine to package together the specific versions they used in an analysis. Alternatively, they might use third-party solutions such as the aRchive project [118].

4. Literate programming combines narratives with code

Although narratives, scripts, and computer code individually support reproducibility, there is additional value in combining these entities. Even though a researcher may provide computer code alongside a research paper, other scientists may have difficulty interpreting how the code accomplishes specific tasks. A longstanding way to address this problem is via code comments: human-readable annotations interspersed throughout computer code. However, code comments and other types of documentation often become outdated as code evolves throughout the analysis process [64]. One way to overcome this problem is to use a technique called literate programming [65]. In this approach, the scientist writes a narrative of the scientific analysis and intermingles code directly within the narrative. As the code is executed, a document is generated that includes the code, narratives, and any outputs (e.g., figures, tables) of the code. Accordingly, literate programming helps ensure that readers understand exactly how a particular research result was obtained. In addition, this approach motivates the scientist to keep the target audience in mind when performing a computational analysis, rather than simply to write code that a computer can parse [65]. Consequently, by reducing barriers of understanding among scientists, literate programming can help to engender greater trust in computational findings.

One popular literate programming tool is Jupyter [66]. Using Jupyter.org's Web-based interface, scientists can create interactive "notebooks" that combine code, data, mathematical equations, plots, and rich media [67]. Originally known as IPython, and previously designed exclusively for the Python programming language, Jupyter now makes

it possible to execute code in many different programming languages. Such functionality may be important to scientists who prefer to combine the strengths of different programming languages.

knitr [68] has also gained considerable popularity as a literate programming tool. It is written in the R programming language, and thus can be integrated seamlessly with the array of statistical and plotting tools available in that environment. However, like Jupyter, knitr can execute code written in multiple programming languages. Commonly, knitr is applied to documents that have been authored using RStudio [69], an open-source tool with advanced editing and package management features.

Jupyter notebooks and knitr reports can be saved in various output formats, including hypertext markup language (HTML) and portable document format (PDF; see examples in Figures 3 and 4; Additional files 3 and 4). Increasingly, scientists include such documents as supplementary materials to journal manuscripts, enabling others to repeat analysis steps and recreate manuscript figures [70–73].

Scientists typically use literate programming tools for data analysis tasks that can be executed interactively, in a modest amount of time (e.g., minutes or hours). However, it is possible to execute Jupyter or knitr at the command line; thus longer running tasks can be executed on high-performance computers.

Literate programming notebooks are suitable for research analyses that require a modest amount of computer code. For analyses needing larger amounts of code, more advanced

programming environments may be more suitable, perhaps in combination with a “literate documentation” tool such as Dexy.it.

5. Workflow management systems enable software to be executed via a graphical user interface

Writing computer scripts and code seems daunting to many researchers. Although various courses and tutorials are helping to make this task less formidable [46, 74–76], many scientists use “workflow management systems” to facilitate the execution of scientific software [77]. Typically managed via a graphical user interface, workflow management systems enable scientists to upload data and process them using existing tools. For multistep analyses, the output from one tool can be used as input to additional tools, resulting in a series of commands known as a workflow.

Galaxy [78, 79] has gained considerable popularity within the bioinformatics community, especially for performing next-generation sequencing analysis. As users construct workflows, Galaxy provides descriptions of how software parameters should be used, examples of how input files should be formatted, and links to relevant discussion forums. To help with processing large data sets and computationally complex algorithms, Galaxy also provides an option to execute workflows on cloud-computing services [80]. In addition, researchers can share workflows with each other at UseGalaxy.org; this feature has enabled the Galaxy team to build a community that encourages reproducibility, helps define best practices, and reduce the time required for novices to get started.

Various other workflow systems are freely available to the research community (see Box 2). For example, VisTrails.org is used by researchers from many disciplines, including climate science, microbial ecology, and quantum mechanics [81]. It enables scientists to design visual workflows, and connect data inputs with analytical modules and the resulting outputs. In addition, VisTrails tracks a full history of how each workflow was created. This capability, referred to as “retrospective provenance”, makes it possible for others to not only reproduce the final version of an analysis, but also to examine previous incarnations of the workflow and how each change influenced the analytical outputs [82].

Box 2: Workflow management tools freely available to the research community.

- Galaxy [78, 79]
- VisTrails [81]
- Kepler-project.org [83]
- CyVerse.org (formerly known as The iPlant Collaborative) [84]
- GenePattern [85, 86, 119]
- Taverna.org.uk [87]
- LONI Pipeline [88, 120]

Although workflow management systems offer many advantages, users must accept tradeoffs. For example, although the teams that develop these tools often provide public servers where users can execute workflows, many scientists share these resources, limiting the computational power or storage space available to execute large-scale analyses in a

timely manner. As an alternative, many scientists install these systems on their own computers; however, configuring and supporting them requires time and expertise. In addition, if a workflow tool does not yet provide a module to support a given analysis, the scientist must create one. This task constitutes additional overheads; however, utilities such as the Galaxy Tool Shed [89] are helping to facilitate this process.

6. Virtual machines encapsulate an entire operating system and software dependencies

Whether within a literate programming notebook, or via a workflow management system, an operating system and relevant software dependencies must be installed before an analysis is executed. The process of identifying, installing, and configuring such dependencies consumes a considerable amount of scientists' time. Different operating systems (and versions thereof) may require different installation and configuration steps. Furthermore, earlier versions of software dependencies, which may currently be installed on a given computer, may be incompatible with—or produce different outputs than—newer versions.

One solution is to use virtual machines, which can encapsulate an entire operating system and all software, scripts, code, and data necessary to execute a computational analysis [90, 91] (Figure 5). Using virtualization software such as VirtualBox or VMWare (see Box 3), a virtual machine can be executed on practically any desktop, laptop, or server, irrespective of the main ("host") operating system on the computer. For example, even though a

scientist's computer may be running a Windows operating system, they may perform an analysis on a Linux operating system that is running concurrently—within a virtual machine—on the same computer. The scientist has full control over the virtual ("guest") operating system, and thus can install software and modify configuration settings as necessary. In addition, a virtual machine can be constrained to use specific amounts of computational resources (e.g., computer memory, processing power), thus enabling system administrators to ensure that multiple virtual machines can be executed simultaneously on the same computer without impacting each other's performance. After executing an analysis, the scientist can export the entire virtual machine to a single, binary file. Other scientists can then use this file to reconstitute the same computational environment that was used for the original analysis. With a few exceptions (see Discussion), these scientists will obtain exactly the same results as the original scientist. This process provides the added benefits that 1) the scientist must only document the installation and configuration steps for a single operating system, 2) other scientists need only install the virtualization software and not individual software components, and 3) analyses can be re-executed indefinitely, so long as the virtualization software remains compatible with current computer systems [92]. The fact that a team of scientists can employ virtual machines to ensure that each team member has the same computational environment is also useful because team members may have different configurations on their host operating systems.

One criticism of using virtual machines to support computational reproducibility is that virtual machine files are large (typically multiple gigabytes), especially if they include raw data files. This imposes a barrier for researchers to share virtual machines with the

research community. One option is to use cloud-computing services (see Box 4). Scientists can execute an analysis in the cloud, take a “snapshot” of their virtual machine, and share it with others in that environment [90, 93]. Cloud-based services typically provide repositories where virtual machine files can easily be stored and shared among users. Despite these advantages, some researchers may prefer their data to reside on local computers, rather than in the cloud—at least while the research is being performed. In addition, cloud-based services may use proprietary software, so virtual machines may only be executable within each provider’s infrastructure. Furthermore, to use a cloud service provider, scientists may need to activate a fee-based account.

When using virtual machines to support reproducibility, it is important that other scientists can not only re-execute the analysis, but also examine the scripts and code used within the virtual machine [94]. Although it is possible for others to examine the contents of a virtual machine directly, it is preferable to store the scripts and code in public repositories—separately from the virtual machine—so others can examine and extend the analysis more easily [95]. In addition, scientists can use a virtual machine that has been prepackaged for a particular research discipline. For example, CloudBioLinux contains a variety of bioinformatics tools commonly used by genomics researchers [96]. The scripts for building this virtual machine are stored in a public repository [121].

Scientists can automate the process of building and configuring virtual machines using tools such as Vagrant or Vortex (see Box 3). For either tool, users can write text-based configuration files that provide instructions for building virtual machines and allocating

computational resources to them. In addition, these configuration files can be used to specify analysis steps [95]. Because these files are text based and relatively small (usually a few kilobytes), scientists can share them easily and track different versions of the files via source control repositories. This approach also mitigates problems that might arise during the analysis stage. For example, even when a computer's host operating system must be reinstalled because of a computer hardware failure, the virtual machine can be recreated with relative ease.

Box 3: Virtual machine software.

Virtualization hypervisors:

- VirtualBox.org (open source)
- XenProject.org (open source)
- VMWare.com (partially open source)

Virtual machine management tools:

- VagrantUP.com (open source)
- Vortex (open source) [122]

Box 4: Commercial cloud-service providers.

- Amazon Web Services [123]
- Rackspace.com/Cloud
- Google Cloud Platform [124]
- Windows Azure [125]

7. Software containers ease the process of installing and configuring dependencies

Software containers are a lighter weight alternative to virtual machines. Like virtual machines, containers encapsulate operating system components, scripts, code, and data into a single package that can be shared with others. Thus, as with virtual machines, analyses executed within a software container should produce identical outputs, irrespective of the underlying operating system or the software that may be installed outside the container (see Discussion for caveats). As is true for virtual machines, multiple containers can be executed simultaneously on a single computer, and each container may contain different software versions and configurations. However, whereas virtual machines include an entire operating system, software containers interface directly with the computer's main operating system and extend it as needed (Figure 6). This design provides less flexibility than virtual machines because containers are specific to a given type of

operating system; however, containers require considerably less computational overhead than virtual machines, and can be initialized much more quickly [97].

The open source Docker.com utility, which has gained popularity among informaticians since its release in 2013, provides the ability to build, execute, and share software containers for Linux-based operating systems. Users specify a Docker container's contents using text-based commands. These instructions can be placed in a "Dockerfile", which other scientists can use to rebuild the container. As with virtual machine configuration files, Dockerfiles are text based, so they can be shared easily, and can be tracked and versioned in source control repositories. Once a Docker container has been built, its contents can be exported to a binary file; these files are generally smaller than virtual machine files, so they can be shared more easily—for example, via hub.Docker.com.

A key feature of Docker containers is that their contents can be stacked in distinct layers (or "images"). Each image includes software components to address a particular need. Within a given research lab, scientists might create general purpose images to support functionality for multiple projects, and specialized images to address the needs of specific projects. An advantage of Docker's modular design is that when images within a container are updated, Docker only needs to track the specific components that have changed; users who wish to update to a newer version must download a relatively small update. In contrast, even a minor change to a virtual machine would require users to export and reshare the entire virtual machine.

Scientists have begun to share Docker images that enable others to execute analyses described in research papers [98–100], and to facilitate benchmarking efforts among researchers in a given subdiscipline. For example, nucleotid.es is a catalog of genome-assembly tools that have been encapsulated in Docker images [101, 102]. Genome assembly tools differ considerably in the dependencies they require, and in the parameters they support. This project provides a means to standardize these assemblers, circumvent the need to install dependencies for each tool, and perform benchmarks across the tools. Such projects may help to reduce the reproducibility burden on individual scientists.

The use of Docker containers for reproducible research comes with caveats. Individual containers are stored and executed in isolation from other containers on the same computer; however, because all containers on a given machine share the same operating system, this isolation is not as complete as it is with virtual machines. This means, for example, that a given container is not guaranteed to have access to a specific amount of computer memory or processing power—multiple containers may have to compete for these resources [97]. In addition, containers may be more vulnerable to security breaches [97]. Because Docker containers can only be executed on Linux-based operating systems, they must be executed within a virtual machine on Windows and Mac operating systems. Docker provides installation packages to facilitate this integration; however, the overhead of using a virtual machine offsets some of the performance benefits of using containers.

Efforts are ongoing to develop and refine software container technologies. Box 5 lists various tools that are currently available. In the coming years, these technologies promise to play an influential role within the scientific community.

Box 5: Open-source containerization software.

- Docker.com
- LinuxContainers.org
- lmctfy [126]
- OpenVZ.org
- Warden [127]

Conclusions

Scientific advancement requires researchers to explicitly document the research steps they performed and to transparently share those steps with other researchers. This review provides a comprehensive, though not exhaustive, list of techniques that can help meet these requirements for computational analyses. Science philosopher Karl Popper contended that, "[w]e do not take even our own observations quite seriously, or accept them as scientific observations, until we have repeated and tested them" [2]. Indeed, in many cases, the individuals who benefit most from computational reproducibility are those

who performed the original analysis, but reproducible and transparent practices can also increase the level at which a scientist's work is accepted by other scientists [47, 103]. When other scientists can reproduce an analysis and determine exactly how its conclusions were drawn, they may be more apt to cite and build upon the work. In contrast, when others fail to reproduce research findings, it can derail scientific progress and may lead to embarrassment, accusations, and retractions.

We have described seven tools and techniques for facilitating computational reproducibility. None of these approaches is sufficient for every scenario in isolation; rather, scientists will often find value in combining approaches. For example, a researcher who uses a literate programming notebook (that combines narratives with code) might incorporate the notebook into a software container so that others can execute it without needing to install specific software dependencies. The container might also include a workflow management system to ease the process of integrating multiple tools and incorporating best practices for the analysis. This container could be packaged within a virtual machine or cloud-computing environment to ensure that it can be executed consistently (see Figure 7). Binder [104] and Everware [128] are two services that allow researchers to execute Jupyter notebooks within a Web browser, using a Docker container to package the underlying software, and a cloud-computing environment to execute it. Although still under active development, such services may be harbingers of the future for computationally reproducible science.

The call for computational reproducibility relies on the premise that reproducible science will bolster the efficiency of the overall scientific enterprise [105]. Although reproducible practices may require additional time and effort, these practices provide ancillary benefits that help offset those expenditures [47]. Primarily, scientists may experience increased efficiency in their research [47]. For example, before and after a manuscript is submitted for publication, it faces scrutiny from co-authors and peer reviewers who may suggest alterations to the analysis. Having a complete record of all the analysis steps, and being able to retrace those steps precisely, makes it faster and easier to implement the requested alterations [47,106]. Reproducible practices can also improve the efficiency of team science because colleagues can more easily communicate their research protocols and inspect each other's work; one type of relationship where this is critical is that between academic advisors and mentees [106]. Finally, when research protocols are shared transparently with the broader community, scientific advancement increases because scientists can learn more easily from each other's work and there is less duplication of effort [106].

Reproducible practices do not necessarily ensure that others can obtain identical results to those obtained by the original scientists. Indeed, this objective may be infeasible for some types of computational analysis, including those that use randomization procedures, floating-point operations, or specialized computer hardware [91, 107]. In such cases, the goal may shift to ensuring that others can obtain results that are semantically consistent with the original findings [5,6]. In addition, in studies where vast computational resources are needed to perform an analysis, or where data sets are distributed geographically [108–110], full reproducibility may be infeasible. Alternatively, it may be infeasible to reallocate

computational resources for highly computationally intensive analyses [8]. In these cases, researchers can provide relatively simple examples to demonstrate the methodology [8]. When legal restrictions prevent researchers from publicly sharing software or data, or when software is available only via a Web interface, researchers should document the analysis steps as well as possible and describe why such components cannot be shared [25].

Computational reproducibility does not guarantee against analytical biases, or ensure that software produces scientifically valid results [111]. As with any research, a poor study design, confounding effects, or improper use of analytical software may plague even the most reproducible analyses [111, 112]. On one hand, increased transparency puts scientists at a greater risk that such problems will be exposed. On the other hand, scientists who are fully transparent about their scientific approach may be more likely to avoid such pitfalls, knowing that they will be more vulnerable to such criticisms. Either way, the scientific community benefits.

Lastly, we emphasize that some reproducibility is better than none. Although some of the practices described in this review require more technical expertise than others, they are freely accessible to all scientists, and provide long-term benefits to the researcher and to the scientific community. Indeed, as scientists act in good faith to perform these practices, where feasible, the pace of scientific progress will surely increase.

List of abbreviations

DOI: Digital object identifier

HTML: Hypertext markup language

PDF: Portable document format

URL: Uniform resource locator

VCS: Version control system

Acknowledgements

SRP acknowledges startup funds provided by Brigham Young University. We thank research-community members and reviewers who provided valuable feedback on this manuscript.

Additional files

Additional file 1: This script is supporting material for Figure 1. It can be used to align DNA sequence data to a reference genome. First, it downloads the software and data files necessary for the analysis. Then, it extracts ("unzips") these files, and aligns the data to a reference genome for Ebola virus. Finally, it converts, sorts, and indexes the aligned data.

Additional file 2: This Make file is supporting material for Figure 2. It performs the same function as Additional file 1, except that it is formatted for the Make utility. Accordingly, it is structured so that specific tasks must be executed before other tasks, in a hierarchical manner.

Additional file 3: This Jupyter notebook is supporting material for Figure 3. It contains code (in the Python programming language) for generating random numbers and plotting them in a graph.

Additional file 4: This document contains code (in the R language) for generating random numbers and plotting them on a graph. This document is in R Markdown format and can be compiled using knitr.

Competing Interests

Neither of the authors of this manuscript have any competing interests to declare.

Authors' contributions

SRP wrote the manuscript and created figures. MBF created figures and helped to revise the manuscript.

References

1. Fisher RA. The Design of Experiments. New York: Hafner Press; 1935.

2. Popper KR. 2002. The logic of scientific discovery. London Routledge; 1959.

3. Peng RD. Reproducible research in computational science. Science. 2011;334:1226–7.

4. Russell JF. If a job is worth doing, it is worth doing twice. Nature. 2013;496:7.

5. Feynman RP, Leighton RB, Sands M. Six Easy Pieces: Essentials of Physics Explained by Its Most Brilliant Teacher. Perseus Books; 1994. p. 34–5.

6. Murray-Rust P, Murray-Rust D. Reproducible Physical Science and the Declaratron. In: Stodden VC, Leisch F, Peng RD, editors. Implementing Reproducible Research. CRC Press; 2014. p. 113.

7. Hey AJG, Tansley S, Tolle KM, Others. The fourth paradigm: data-intensive scientific discovery. Microsoft Research Redmond, WA; 2009.

8. Millman KJ, Pérez F. Developing Open-Source Scientific Practice. Implementing Reproducible Research. CRC Press; 2014;149.

9. Wilson G, Aruliah DA, Brown CT, Chue Hong NP, Davis M, Guy RT, et al. Best practices for scientific computing. PLoS Biol. 2014;12:e1001745.

10. Software with impact. Nat. Methods. 2014;11:211.

11. Hong NC. We are the 92% [Internet]. Figshare; 2014. Available from: http://dx.doi.org/10.6084/M9.FIGSHARE.1243288

12. Sacks J, Welch WJ, Mitchell TJ, Wynn HP. Design and Analysis of Computer Experiments. Stat. Sci. Institute of Mathematical Statistics; 1989;4:409–23.

13. Garijo D, Kinnings S, Xie L, Xie L, Zhang Y, Bourne PE, et al. Quantifying reproducibility in computational biology: the case of the tuberculosis drugome. PLoS One. 2013;8:e80278.

14. Error prone. Nature. 2012;487:406.

15. Vandewalle P, Barrenetxea G, Jovanovic I, Ridolfi A, Vetterli M. Experiences with Reproducible Research in Various Facets of Signal Processing Research. 2007 IEEE International Conference on Acoustics, Speech and Signal Processing - ICASSP '07. IEEE; 2007. p. IV – 1253 – IV – 1256.

16. Cassey P, Cassey P, Blackburn T, Blackburn T. Reproducibility and Repeatability in Ecology. Bioscience. 2006;56:958–9.

17. Murphy JM, Sexton DMH, Barnett DN, Jones GS, Webb MJ, Collins M, et al. Quantification of modelling uncertainties in a large ensemble of climate change simulations. Nature. 2004;430:768–72.

18. McCarthy DJ, Humburg P, Kanapin A, Rivas MA, Gaulton K, Cazier J-B, et al. Choice of transcripts and software has a large effect on variant annotation. Genome Med. 2014;6:26.

19. Neuman JA, Isakov O, Shomron N. Analysis of insertion-deletion from deep-sequencing

data: Software evaluation for optimal detection. Brief. Bioinform. 2013;14:46–55.

20. Bradnam KR, Fass JN, Alexandrov A, Baranay P, Bechner M, Birol I, et al. Assemblathon 2: evaluating de novo methods of genome assembly in three vertebrate species. Gigascience. 2013;2:10.

21. Bilal E, Dutkowski J, Guinney J, Jang IS, Logsdon BA, Pandey G, et al. Improving Breast Cancer Survival Analysis through Competition-Based Multidimensional Modeling. PLoS Comput. Biol. 2013;9:e1003047.

22. Gronenschild EHBM, Habets P, Jacobs HIL, Mengelers R, Rozendaal N, van Os J, et al. The effects of FreeSurfer version, workstation type, and Macintosh operating system version on anatomical volume and cortical thickness measurements. PLoS One. 2012;7:e38234.

23. Moskvin OV, McIlwain S, Ong IM. CAMDA 2014: Making sense of RNA-Seq data: From low-level processing to functional analysis. Systems Biomedicine. 2014;2:31–40.

24. Reducing our irreproducibility. Nature. 2013;496:398–398.

25. Michael CM, Nass SJ, Omenn GS, editors. Evolution of Translational Omics: Lessons Learned and the Path Forward. Washington, D.C.: The National Academies Press; 2012.

26. Collins FS, Tabak L a. Policy: NIH plans to enhance reproducibility. Nature. 2014;505:612–3.

27. Chambers JM. S as a Programming Environment for Data Analysis and Graphics. Problem Solving Environments for Scientific Computing, Proc. 17th Symp. on the Interface

of Stat. and Comp. North Holland; 1985. p. 211–4.

28. LeVeque RJ, Mitchell IM, Stodden V. Reproducible research for scientific computing: Tools and strategies for changing the culture. Computing in Science and Engineering. 2012;14:13.

29. Stodden V, Guo P, Ma Z. Toward Reproducible Computational Research: An Empirical Analysis of Data and Code Policy Adoption by Journals. PLoS One. 2013;8:2–9.

30. Morin A, Urban J, Adams PD, Foster I, Sali A, Baker D, et al. Research priorities. Shining light into black boxes. Science. 2012;336:159–60.

31. Rebooting review. Nat. Biotechnol. 2015;33:319.

32. Ioannidis JP a., Allison DB, Ball C a., Coulibaly I, Cui X, Culhane AC, et al. Repeatability of published microarray gene expression analyses. Nat. Genet. 2009;41:149–55.

33. Nekrutenko A, Taylor J. Next-generation sequencing data interpretation: enhancing reproducibility and accessibility. Nat. Rev. Genet. Nature Publishing Group; 2012;13:667–72.

34. Baggerly K a., Coombes KR. Deriving chemosensitivity from cell lines: Forensic bioinformatics and reproducible research in high-throughput biology. Ann. Appl. Stat. 2009;3:1309–34.

35. Decullier E, Huot L, Samson G, Maisonneuve H. Visibility of retractions: a cross-sectional one-year study. BMC Res. Notes. 2013;6:238.

36. Claerbout JF, Karrenbach M. Electronic Documents Give Reproducible Research a New Meaning. Meeting of the Society of Exploration Geophysics. New Orleans, LA; 1992.

37. Stodden V, Miguez S. Best Practices for Computational Science: Software Infrastructure and Environments for Reproducible and Extensible Research. Journal of Open Research Software. 2014;2:21.

38. Ravel J, Wommack KE. All hail reproducibility in microbiome research. Microbiome. 2014;2:8.

39. Stodden V. 2014: What scientific idea is ready for retirement? [Internet]. http://edge.org/response-detail/25340. 2014. Available from: http://edge.org/response-detail/25340

40. Birney E, Hudson TJ, Green ED, Gunter C, Eddy S, Rogers J, et al. Prepublication data sharing. Nature. 2009;461:168–70.

41. Hothorn T, Leisch F. Case studies in reproducibility. Brief. Bioinform. 2011;12:288–300.

42. Schofield PN, Bubela T, Weaver T, Portilla L, Brown SD, Hancock JM, et al. Post-publication sharing of data and tools. Nature. 2009;461:171–3.

43. Piwowar H a., Day RS, Fridsma DB. Sharing detailed research data is associated with increased citation rate. PLoS One. 2007;2. http://dx.doi.org/10.1371/journal.pone.0000308

44. Johnson VE. Revised standards for statistical evidence. Proc. Natl. Acad. Sci. U. S. A.

2013;110:19313–7.

45. Halsey LG, Curran-everett D, Vowler SL, Drummond GB. The fickle P value generates irreproducible results. Nat. Methods. 2015;12:179–85.

46. Wilson G. Software Carpentry: lessons learned. F1000Res. 2016;3:62.

47. Sandve GK, Nekrutenko A, Taylor J, Hovig E. Ten Simple Rules for Reproducible Computational Research. PLoS Comput. Biol. 2013;9:1–4.

48. Köster J, Rahmann S. Snakemake--a scalable bioinformatics workflow engine. Bioinformatics. 2012;28:2520–2.

49. Sadedin SP, Pope B, Oshlack A. Bpipe : A Tool for Running and Managing Bioinformatics Pipelines. Bioinformatics. 2012;28:1525–6.

50. Tange O. GNU Parallel - The Command-Line Power Tool. ;login: The USENIX Magazine. Frederiksberg, Denmark; 2011;36:42–7.

51. Albrecht M, Donnelly P, Bui P, Thain D. Makeflow: A portable abstraction for data intensive computing on clusters, clouds, and grids. Proceedings of the 1st ACM SIGMOD Workshop on Scalable Workflow Execution Engines and Technologies. 2012.

52. Knight S, Austin C, Crain C, Leblanc S, Roach A. Scons software construction tool [Internet]. 2011. Available from: http://www.scons.org

53. Code share. Nature. 2014;514:536.

54. Blischak JD, Davenport ER, Wilson G. A Quick Introduction to Version Control with Git and GitHub. PLoS Comput. Biol. 2016;12:e1004668.

55. Loeliger J, McCullough M. Version Control with Git: Powerful Tools and Techniques for Collaborative Software Development. "O'Reilly Media, Inc."; 2012. p. 456.

56. Huber W, Carey VJ, Gentleman R, Anders S, Carlson M, Carvalho BS, et al. Orchestrating high-throughput genomic analysis with Bioconductor. Nat. Methods. Nature Publishing Group; 2015;12:115–21.

57. R Core Team. R: A Language and Environment for Statistical Computing [Internet]. Vienna, Austria: R Foundation for Statistical Computing; 2014. Available from: http://www.r-project.org

58. Tóth G, Sokolov IV, Gombosi TI, Chesney DR, Clauer CR, De Zeeuw DL, et al. Space Weather Modeling Framework: A new tool for the space science community. J. Geophys. Res. 2005;110:A12226.

59. Tan E, Choi E, Thoutireddy P, Gurnis M, Aivazis M. GeoFramework: Coupling multiple models of mantle convection within a computational framework. Geochem. Geophys. Geosyst. [Internet]. 2006;7. Available from: http://doi.wiley.com/10.1029/2005GC001155

60. Heisen B, Boukhelef D, Esenov S, Hauf S, Kozlova I, Maia L, et al. Karabo: An Integrated Software Framework Combining Control, Data Management, and Scientific Computing Tasks. 14th International Conference on Accelerator & Large Experimental Physics Control Systems, ICALEPCS2013. San Francisco, CA; 2013.

61. Schneider CA, Rasband WS, Eliceiri KW. NIH Image to ImageJ: 25 years of image analysis. Nat. Methods. Nature Publishing Group; 2012;9:671–5.

62. Schindelin J, Arganda-Carreras I, Frise E, Kaynig V, Longair M, Pietzsch T, et al. Fiji: an open-source platform for biological-image analysis. Nat. Methods. Nature Publishing Group, a division of Macmillan Publishers Limited. All Rights Reserved.; 2012;9:676–82.

63. Biasini M, Schmidt T, Bienert S, Mariani V, Studer G, Haas J, et al. OpenStructure: an integrated software framework for computational structural biology. Acta Crystallogr. D Biol. Crystallogr. 2013;69:701–9.

64. Martin RC. Clean code: a handbook of agile software craftsmanship. Pearson Education; 2009.

65. Knuth DE. Literate Programming. Comput. J. 1984;27:97–111.

66. Pérez F, Granger BE. IPython: a System for Interactive Scientific Computing. Computing in Science and Engineering. IEEE Computer Society; 2007;9:21–9.

67. Shen H. Interactive notebooks: Sharing the code. Nature. 2014;515:151–2.

68. Xie Y. Dynamic Documents with R and knitr. CRC Press; 2013. p. 216.

69. RStudio Team. RStudio: Integrated Development for R [Internet]. [cited 2015 Nov 20]. Available from: http://www.rstudio.com

70. Gross AM, Orosco RK, Shen JP, Egloff AM, Carter H, Hofree M, et al. Multi-tiered genomic analysis of head and neck cancer ties TP53 mutation to 3p loss. Nat. Genet. Nature

Publishing Group; 2014;46:1–7.

71. Ding T, Schloss PD. Dynamics and associations of microbial community types across the human body. Nature. Nature Publishing Group, a division of Macmillan Publishers Limited. All Rights Reserved.; 2014;509:357–60.

72. Ram Y, Hadany L. The probability of improvement in Fisher's geometric model: A probabilistic approach. Theor. Popul. Biol. 2015;99:1–6.

73. Meadow JF, Altrichter AE, Kembel SW, Moriyama M, O'Connor TK, Womack AM, et al. Bacterial communities on classroom surfaces vary with human contact. Microbiome. 2014;2:7.

74. White E. Programming for Biologists [Internet]. Available from: http://www.programmingforbiologists.org

75. Peng RD. Coursera course: Computing for Data Analysis [Internet]. Available from: https://www.coursera.org/course/compdata

76. Bioconductor - Courses and Conferences [Internet]. [cited 2015 Nov 20]. Available from: http://master.bioconductor.org/help/course-materials

77. Gil Y, Deelman E, Ellisman M, Fahringer T, Fox G, Gannon D, et al. Examining the challenges of scientific workflows. Computer . 2007;40:24–32.

78. Giardine B, Riemer C, Hardison RC, Burhans R, Elnitski L, Shah P, et al. Galaxy: a platform for interactive large-scale genome analysis. Genome Res. 2005;15:1451–5.

79. Goecks J, Nekrutenko A, Taylor J. Galaxy: a comprehensive approach for supporting accessible, reproducible, and transparent computational research in the life sciences. Genome Biol. 2010;11:R86.

80. Afgan E, Baker D, Coraor N, Goto H, Paul IM, Makova KD, et al. Harnessing cloud computing with Galaxy Cloud. Nat. Biotechnol. 2011;29:972–4.

81. Callahan SP, Freire J, Santos E, Scheidegger CE, Silva CT, Vo HT. VisTrails: Visualization Meets Data Management. Proceedings of the 2006 ACM SIGMOD International Conference on Management of Data. New York, NY, USA: ACM; 2006. p. 745–7.

82. Davidson SB, Freire J. Provenance and scientific workflows. Proceedings of the 2008 ACM SIGMOD international conference on Management of data - SIGMOD ’08. 2008. p. 1345.

83. Altintas I, Berkley C, Jaeger E, Jones M, Ludascher B, Mock S. Kepler: an extensible system for design and execution of scientific workflows. Proceedings. 16th International Conference on Scientific and Statistical Database Management, 2004. IEEE; 2004. p. 423–4.

84. Goff SA, Vaughn M, McKay S, Lyons E, Stapleton AE, Gessler D, et al. The iPlant Collaborative: Cyberinfrastructure for Plant Biology. Front. Plant Sci. Frontiers; 2011;2:34.

85. Reich M, Liefeld T, Gould J, Lerner J, Tamayo P, Mesirov JP. GenePattern 2.0. Nat. Genet. 2006;38:500–1.

86. Reich M, Liefeld J, Thorvaldsdottir H, Ocana M, Polk E, Jang D, et al. GenomeSpace: An

environment for frictionless bioinformatics. Cancer Res. 2012;72:3966–3966.

87. Wolstencroft K, Haines R, Fellows D, Williams A, Withers D, Owen S, et al. The Taverna workflow suite: designing and executing workflows of Web Services on the desktop, web or in the cloud. Nucleic Acids Res. 2013;41:557–61.

88. Rex DE, Ma JQ, Toga AW. The LONI Pipeline Processing Environment. Neuroimage. 2003;19:1033–48.

89. Lazarus R, Kaspi A, Ziemann M. Creating re-usable tools from scripts: The Galaxy Tool Factory. Bioinformatics. 2012;28:3139–40.

90. Dudley JT, Butte AJ. In silico research in the era of cloud computing. Nat. Biotechnol. Nature Publishing Group; 2010;28:1181–5.

91. Hurley DG, Budden DM, Crampin EJ. Virtual Reference Environments: a simple way to make research reproducible. Brief. Bioinform. 2014;1–3.

92. Gent IP. The Recomputation Manifesto. arXiv [Internet]. 2013; Available from: http://arxiv.org/abs/1304.3674

93. Howe B. Virtual Appliances, Cloud Computing, and Reproducible Research. Comput. Sci. Eng. IEEE Computer Society; 2012;14:36–41.

94. Brown CT. Virtual machines considered harmful for reproducibility [Internet]. 2012. Available from: http://ivory.idyll.org/blog/vms-considered-harmful.html

95. Piccolo SR. Building portable analytical environments to improve sustainability of

computational-analysis pipelines in the sciences [Internet]. 2014. Available from: http://dx.doi.org/10.6084/m9.figshare.1112571

96. Krampis K, Booth T, Chapman B, Tiwari B, Bicak M, Field D, et al. Cloud BioLinux: pre-configured and on-demand bioinformatics computing for the genomics community. BMC Bioinformatics. 2012;13:42.

97. Felter W, Ferreira A, Rajamony R, Rubio J. An Updated Performance Comparison of Virtual Machines and Linux Containers [Internet]. IBM Research Division; 2014. Available from: http://domino.research.ibm.com/library/CyberDig.nsf/papers/0929052195DD819C85257D2300681E7B/$File/rc25482.pdf

98. Eglen SJ, Weeks M, Jessop M, Simonotto J, Jackson T, Sernagor E. A data repository and analysis framework for spontaneous neural activity recordings in developing retina. Gigascience. 2014;3:3.

99. Eglen SJ. Bivariate spatial point patterns in the retina: a reproducible review. Journal de la Société Française de Statistique. 2016;157:33–48.

100. Bremges A, Maus I, Belmann P, Eikmeyer F, Winkler A, Albersmeier A, et al. Deeply sequenced metagenome and metatranscriptome of a biogas-producing microbial community from an agricultural production-scale biogas plant. Gigascience. 2015;4:33.

101. Belmann P, Dröge J, Bremges A, McHardy AC, Sczyrba A, Barton MD. Bioboxes: standardised containers for interchangeable bioinformatics software. Gigascience.

2015;4:47.

102. Barton M. nucleotides · genome assembler benchmarking [Internet]. [cited 2015 Nov 20]. Available from: http://nucleotid.es

103. Hones MJ. Reproducibility as a Methodological Imperative in Experimental Research. PSA: Proceedings of the Biennial Meeting of the Philosophy of Science Association. Philosophy of Science Association; 1990. p. 585–99.

104. Rosenberg DM, Horn CC. Neurophysiological analytics for all! Free open-source software tools for documenting, analyzing, visualizing, and sharing using electronic notebooks. J. Neurophysiol. American Physiological Society; 2016;jn.00137.2016.

105. Crick T. " Share and Enjoy ": Publishing Useful and Usable Scientific Models. Available from: http://arxiv.org/abs/1409.0367v2

106. Donoho DL. An invitation to reproducible computational research. Biostatistics. 2010;11:385–8.

107. Goldberg D. What Every Computer Scientist Should Know About Floating-point Arithmetic. ACM Comput. Surv. New York, NY, USA: ACM; 1991;23:5–48.

108. Shirts M, Pande VS. COMPUTING: Screen Savers of the World Unite! Science. 2000;290:1903–4.

109. Bird I. Computing for the Large Hadron Collider. Annu. Rev. Nucl. Part. Sci. 2011;61:99–118.

110. Anderson DP. BOINC: A System for Public Resource Computing and Storage. Proceedings of the Fifth IEEE/ACM International Workshop on Grid Computing (GRID'04). 2004.

111. Ransohoff DF. Bias as a threat to the validity of cancer molecular-marker research. Nat. Rev. Cancer. 2005;5:142–9.

112. Bild AH, Chang JT, Johnson WE, Piccolo SR. A field guide to genomics research. PLoS Biol. 2014;12:e1001744.

113. GNU Make [Internet]. 2016. Available from https://www.gnu.org/software/make

114. Make for Windows [Internet]. 2016. Available from http://gnuwin32.sourceforge.net/packages/make.htm

115. Ivy, the agile dependency manager [Internet]. 2016. Available from http://ant.apache.org/ivy

116. Puppet [Internet]. 2016. Available from https://puppetlabs.com

117. aRchive: Enabling reproducibility of Bioconductor package versions (for Galaxy) [Internet]. 2016. Available from http://bioarchive.github.io

118. GenePattern: A platform for reproducible bioinformatics [Internet]. 2016. Available from http://www.broadinstitute.org/cancer/software/genepattern]

119. LONI Pipeline Processing Environment [Internet]. 2016. Available from http://www.loni.usc.edu/Software/Pipeline

120. CloudBioLinux: configure virtual (or real) machines with tools for biological analyses [Internet]. 2016. Available from https://github.com/chapmanb/cloudbiolinux

121. Vortex [Internet]. 2016. Available from https://github.com/websecurify/node-vortex

122. Amazon Web Services [Internet]. 2016. Available from http://aws.amazon.com

123. Google Cloud Platform [Internet]. 2016. Available from https://cloud.google.com/compute

124. Microsoft Azure [Internet]. 2016. Available from https://azure.microsoft.com

125. lmctfy - Let Me Contain That For You [Internet]. 2016. Available from https://github.com/google/lmctfy

126. Warden [Internet]. 2016. Available from http://docs.cloudfoundry.org/concepts/architecture/warden.html

127. everware [Internet]. 2016. Available from https://github.com/everware/everware

Figure legends

Figure 1. Example of a command line script. This script can be used to align DNA sequence data to a reference genome. First, it downloads the software and data files necessary for the analysis. Then, it extracts ("unzips") these files, and aligns the data to a reference genome for Ebola virus. Finally, it converts, sorts, and indexes the aligned data. See Additional file 1 for an executable version of this script.

Figure 2. Example of a Make file. This file performs the same function as the command line script shown in Figure 1, except that it is formatted for the Make utility. Accordingly, it is structured so that specific tasks must be executed before other tasks, in a hierarchical manner. See Additional file 2 for an executable version of this file.

Figure 3. Example of a Jupyter notebook. This example contains code (in the Python programming language) for generating random numbers and plotting them in a graph within a Jupyter notebook. Importantly, the code and output object (graph) are contained within the same document. See Additional file 3 for an executable version of the notebook.

Figure 4. Example of a document created using knitr. This example contains code (in the R language) for generating random numbers and plotting them on a graph. The knitr tool was used to generate the document, which combines the code and the output object (figure). See Additional file 4 for an executable version of this document.

Figure 5. Architecture of virtual machines. Virtual machines encapsulate analytical software and dependencies within a "guest" operating system, which may be different to

the main (“host”) operating system. A virtual machine executes in the context of virtualization software, which runs alongside other software installed on the computer.

Figure 6. Architecture of software containers. Software containers encapsulate analytical software and dependencies. In contrast to virtual machines, containers execute within the context of the computer’s main operating system.

Figure 7. Example of a Docker container for genomics research. This container would enable researchers to preprocess various types of molecular data, using tools from Bioconductor and Galaxy, and to analyze the resulting data within a Jupyter notebook. Each box within the container represents a distinct Docker image. These images are layered such that some images depend on others (for example, the Bioconductor image depends on R). At its base, the container includes operating system libraries, which may not be present (or may be configured differently) on the computer’s main operating system.

```
#!/bin/bash

# Download software, reference genome, and FASTQ files
wget http://downloads.sourceforge.net/project/bio-bwa/bwakit/bwakit-0.7.12_x64-
linux.tar.bz2
wget -O refGenomeFiles.zip https://ndownloader.figshare.com/files/4841809
wget -O FASTQ.zip https://ndownloader.figshare.com/articles/3114454/versions/1

# Extract software and reference genome files
tar -jxvf bwakit-0.7.12_x64-linux.tar.bz2
unzip refGenomeFiles.zip
unzip FASTQ.zip

# Align FASTQ data to reference genome and save to SAM file
bwa.kit/bwa mem  KJ660346.fa SRR1972917_1.fastq SRR1972917_1.fastq >
SRR1972917_aligned.sam

# Convert SAM file to BAM file
bwa.kit/samtools view -bS SRR1972917_aligned.sam > SRR1972917_aligned.bam

# Sort BAM file
bwa.kit/samtools sort SRR1972917_aligned.bam SRR1972917_aligned_sorted

# Index BAM file
bwa.kit/samtools index SRR1972917_aligned_sorted.bam
```

Figure 2

Click here to download Figure Figure_2.jpg

```
all: SRR1972917_aligned_sorted.bam

SRR1972917_aligned_sorted.bam: SRR1972917_aligned.sam
	# Convert SAM file to BAM file
	bwa.kit/samtools view -bS SRR1972917_aligned.sam > SRR1972917_aligned.bam

	# Sort BAM file
	bwa.kit/samtools sort SRR1972917_aligned.bam SRR1972917_aligned_sorted

	# Index BAM file
	bwa.kit/samtools index SRR1972917_aligned_sorted.bam

SRR1972917_aligned.sam: bwa.kit/bwa KJ660346.fa SRR1972917_1.fastq
	# Align FASTQ data to reference genome and save to SAM file
	bwa.kit/bwa mem  KJ660346.fa SRR1972917_1.fastq SRR1972917_1.fastq > SRR1972917_aligned.sam

bwa.kit/bwa:
	# Download and unpack BWA software
	wget http://downloads.sourceforge.net/project/bio-bwa/bwakit/bwakit-0.7.12_x64-linux.tar.bz2
	tar -jxvf bwakit-0.7.12_x64-linux.tar.bz2

KJ660346.fa:
	# Download and unzip reference genome
	wget -O refGenomeFiles.zip https://ndownloader.figshare.com/files/4841809
	unzip refGenomeFiles.zip

SRR1972917_1.fastq:
	# Download and unzip FASTQ files
	wget -O FASTQ.zip https://ndownloader.figshare.com/articles/3114454/versions/1
	unzip FASTQ.zip

clean:
	rm -rfv KJ660346.fa* FASTQ.zip refGenomeFiles.zip bwa.kit* bwakit-0.7.12_x64-linux.tar.bz2 SRR1972917*
```

Click here to download Figure Figure_3.jpg

Load Python libraries

In [1]:
```
get_ipython().magic('matplotlib inline')

import matplotlib.pyplot as plt
from numpy.random import normal
```

Generate random numbers simulating gene-expression values

In [2]:
```
geneA = normal(size=1000)
geneB = normal(size=1000)
```

Plot the values

In [4]:
```
plt.plot(geneA, geneB, "o", color="#99C1C2")
plt.xlabel("Gene A")
plt.ylabel("Gene B")
plt.show()
```

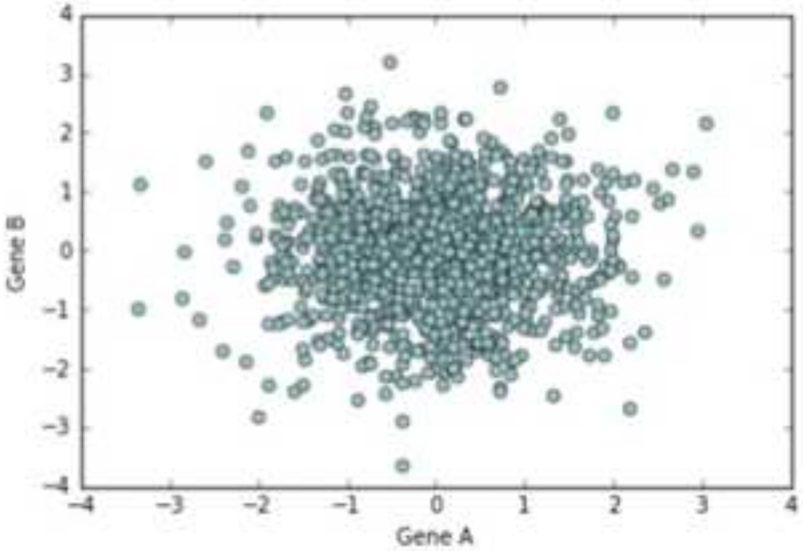

Figure 4

Click here to download Figure Figure_4.jpg

R Markdown Example

Generate random numbers simulating gene-expression values

```
geneA <- rnorm(1000)
geneB <- rnorm(1000)
```

Plot the numbers as a histogram

```
# Set the margins so there won't be too much white space
par(mar=c(4.1, 4.1, 0.1, 0.1))

plot(geneA, geneB, col="#99C1C2", xlab="Gene A", ylab="Gene B", main="")
```

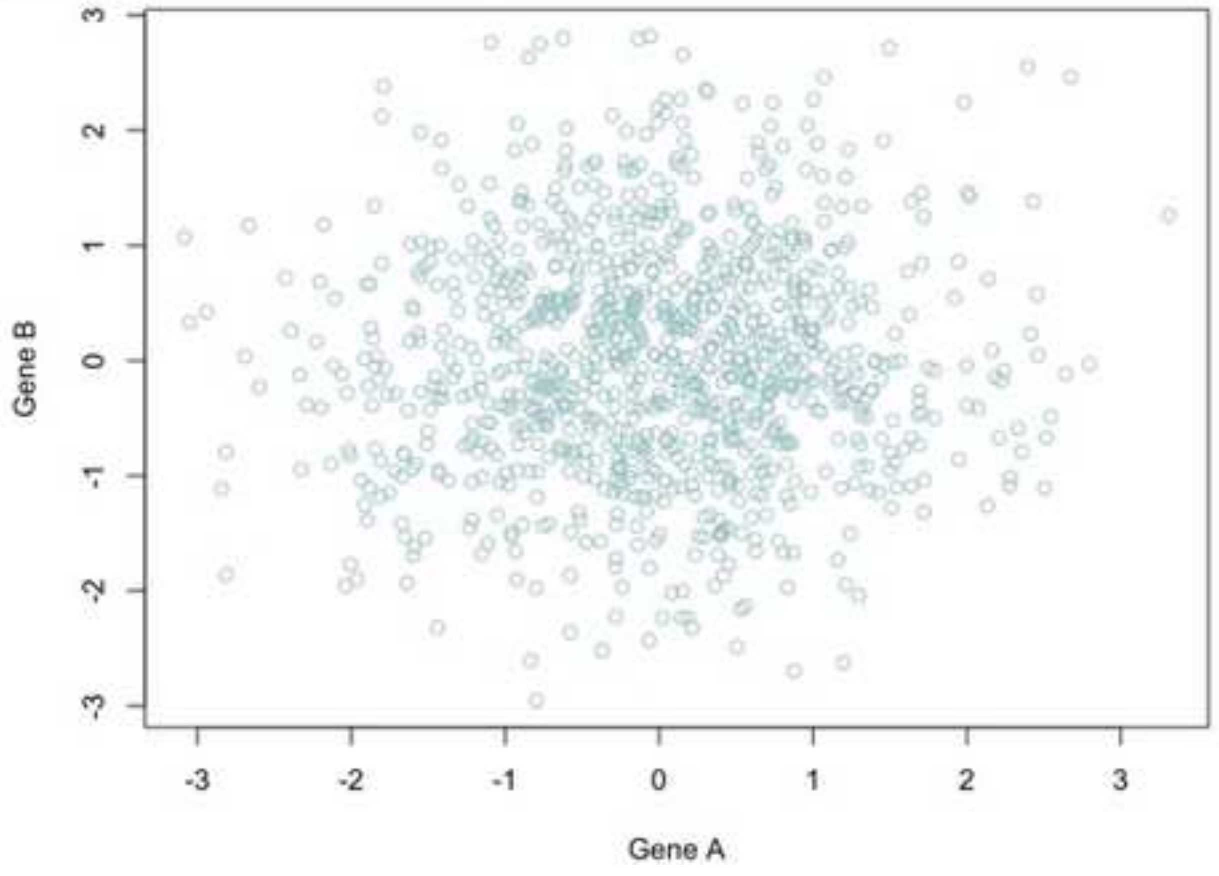

Figure 5

Click here to download Figure Figure_5.jpg

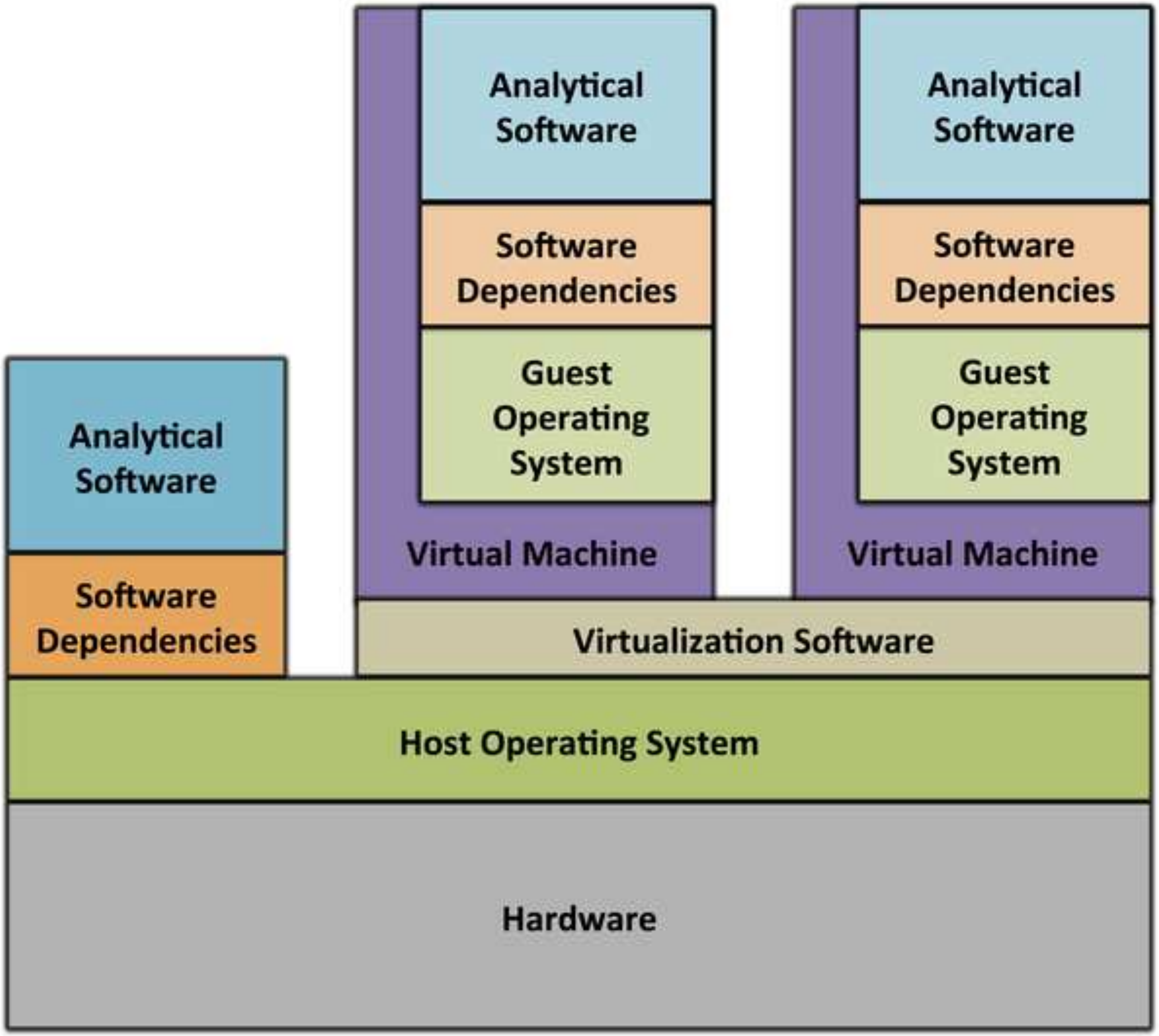

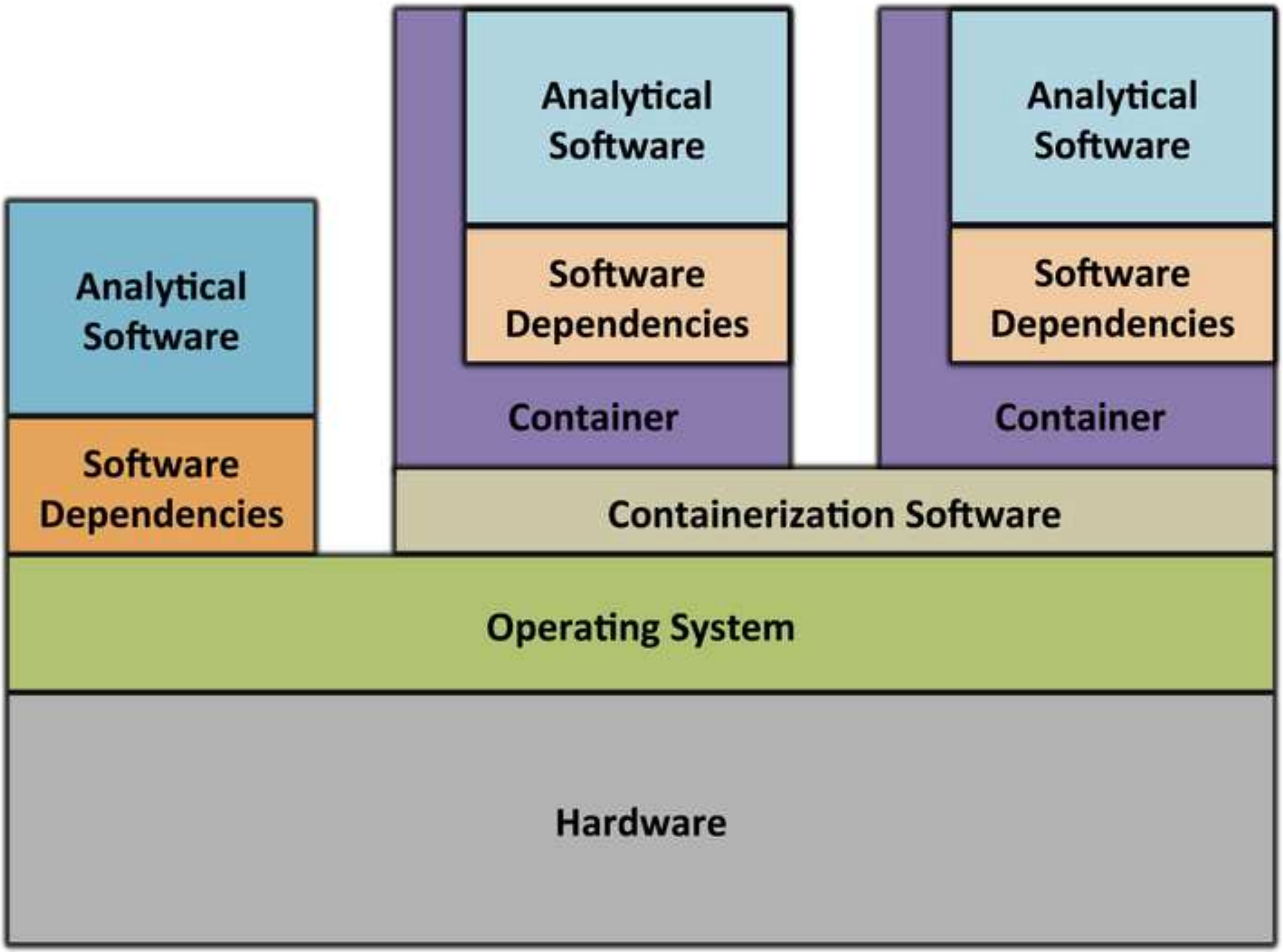
Analytical Software
Software Dependencies
Analytical Software
Software Dependencies
Container
Analytical Software
Software Dependencies
Container
Containerization Software
Operating System
Hardware

Figure 7 Click here to download Figure Figure_7.jpg

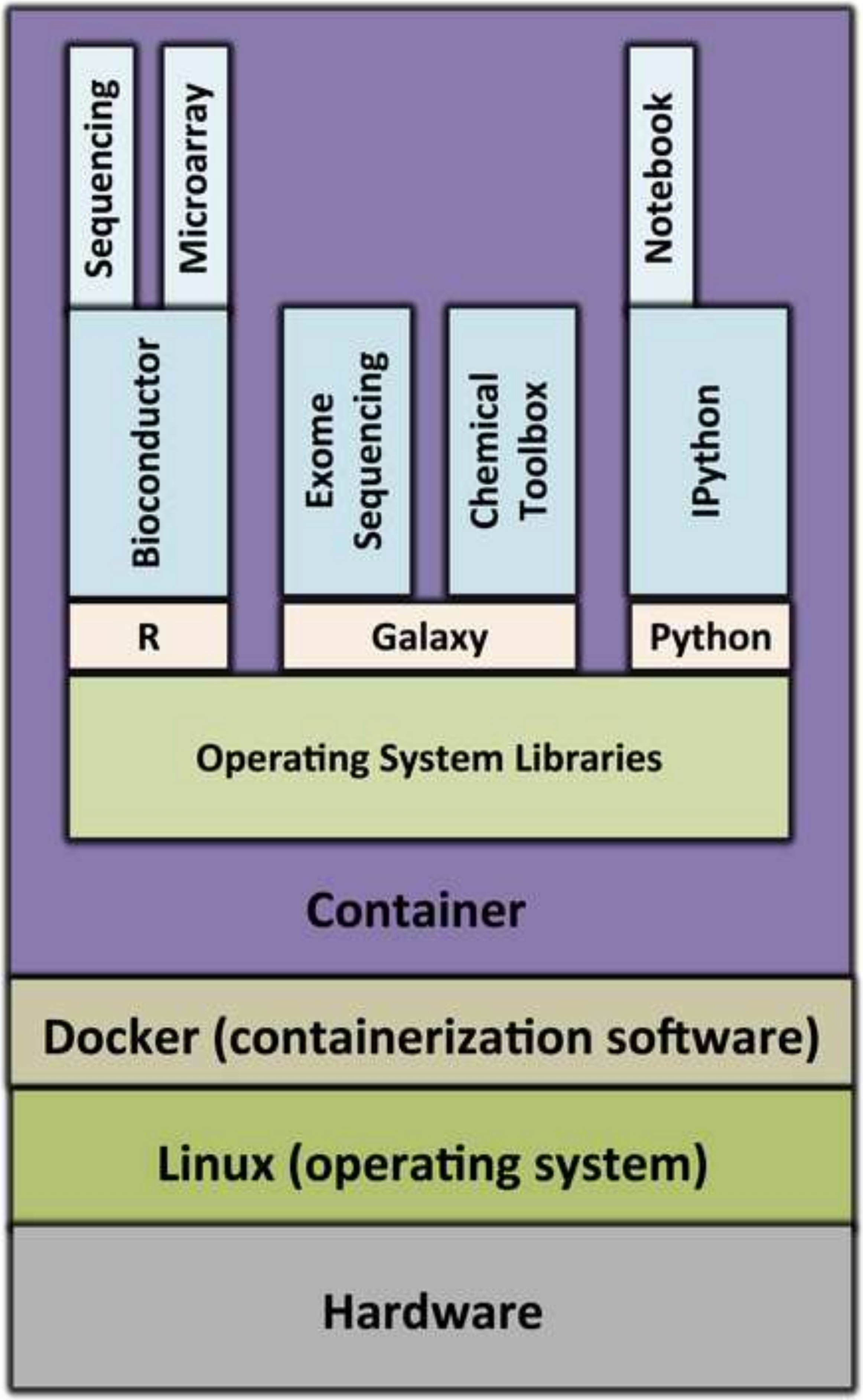

Supplementary File 1

Click here to access/download
Supplementary Material
Script_Example.sh

Supplementary File 2

Click here to access/download
Supplementary Material
Makefile_Example

Supplementary File 3

Click here to access/download
Supplementary Material
Jupyter_Example.ipynb

Supplementary File 4

Click here to access/download
Supplementary Material
Markdown_Example.Rmd
